# Gene Networks and Pathways Involved in *Escherichia coli* Response to Multiple Stressors

**DOI:** 10.3390/microorganisms10091793

**Published:** 2022-09-06

**Authors:** Eman K. Abdelwahed, Nahla A. Hussein, Ahmed Moustafa, Nayera A. Moneib, Ramy K. Aziz

**Affiliations:** 1 Department of Microbiology and Immunology, Faculty of Pharmacy, Cairo University, Cairo 11562, Egypt; 2 Molecular Biology Department, Biotechnology Research Institute, National Research Centre, Giza 12622, Egypt; 3 Department of Biology, and Bioinformatics and Integrative Genomics Lab, American University in Cairo, New Cairo 11835, Egypt; 4 The Center for Genome and Microbiome Research, Faculty of Pharmacy, Cairo University, Cairo 11562, Egypt; 5 Microbiology and Immunology Research Program, Children’s Cancer Hospital (Egypt 57357), Cairo 11617, Egypt

**Keywords:** gene networks, transcriptomics, oxidative stress, nitrosative stress, heat shock, cold shock, antibiotics

## Abstract

Stress response helps microorganisms survive extreme environmental conditions and host immunity, making them more virulent or drug resistant. Although both reductionist approaches investigating specific genes and systems approaches analyzing individual stress conditions are being used, less is known about gene networks involved in multiple stress responses. Here, using a systems biology approach, we mined hundreds of transcriptomic data sets for key genes and pathways involved in the tolerance of the model microorganism *Escherichia coli* to multiple stressors. Specifically, we investigated the *E. coli* K-12 MG1655 transcriptome under five stresses: heat, cold, oxidative stress, nitrosative stress, and antibiotic treatment. Overlaps of transcriptional changes between studies of each stress factor and between different stressors were determined: energy-requiring metabolic pathways, transport, and motility are typically downregulated to conserve energy, while genes related to survival, *bona fide* stress response, biofilm formation, and DNA repair are mainly upregulated. The transcription of 15 genes with uncharacterized functions is higher in response to multiple stressors, which suggests they may play pivotal roles in stress response. In conclusion, using rank normalization of transcriptomic data, we identified a set of *E. coli* stress response genes and pathways, which could be potential targets to overcome antibiotic tolerance or multidrug resistance.

## 1. Introduction

As a common human pathogen, *Escherichia coli* faces and survives numerous stresses, either in the environment or inside the host during infection. Bacterial stress response systems sense, respond, and enable bacterial adaptation to physical and chemical challenges, such as shifts in pH, temperature, antibiotics, nutrition limitation, and oxidative stress. In certain cases, an adaptive response toward one stress provides an acquired resistance to a second one, a phenomenon known as cross-protection. In *E. coli*, stress response systems are mainly centered around two major mechanisms: (i) protein degradation through different sigma factors and (ii) phosphorylation cascades, functioning through two-component regulatory systems. Regardless of the original mechanisms, activation of stress response systems results in a transcriptional modulation [1].

Gene expression reprogramming under different stress conditions has been explored in many studies [2,3,4]. Stress-induced response generates changes in efflux systems, virulence determinants [5], membrane integrity [6], motility, translation, and transcription [3]. It also induces biofilm formation and leads to the generation of more antibiotic-resistant bacteria [7]. Although there are shared features in all stress responses [1], several studies identified specific stress responses with their cognate protein machinery and tried to identify central stress response genes which might be linked to cross-protection [3].

The bacterial adaptive stress response is rapid and reproducible because it involves the induction of a common set of genes or pathways, which lead to a phenotypic plasticity [3]. Although genes involved in specific stress responses have been widely studied, underlying mechanisms in *E. coli* remain to be explored. Most studies describe the alteration of *E. coli* transcriptional profiles in response to specific stresses; however, many gaps still exist in our knowledge. For example, only a handful of studies explore stress response as a complex network, with central proteins controlling underlying mechanisms [8]. Network studies elucidate novel conclusions by summarizing big information into one picture [9]. In particular, a few comparative studies explored stress tolerance across multiple stress conditions for the commonly used *E. coli* strains [10].

In this work, we conducted a thorough investigation of transcriptomic data of the responses of *E. coli* K-12 MG1655 to five different stressors. Through an integrated systems biology approach, we identified common gene sets connecting and regulating important biochemical pathways and biological functions. Therefore, these gene sets can be considered part of the general stress response and candidates to improve tolerance towards multiple stressors (cross-protection) or factors promoting multidrug resistance. In addition to providing a comprehensive overview of the stress responses to understand the control of this complex system, this study highlights the genes and pathways that are common among different stressors and treatment with antibiotics. Therefore, pathways involved in bacterial stress response could be potential targets to overcome antibiotic tolerance or multidrug resistance.

## 2. Materials and Methods

### 2.1. Selection of Stressors, Growth Conditions, and Data Sets

We investigated the *E. coli* K-12 MG1655 transcriptome under five categories of different stresses: heat, cold, oxidative stress, nitrosative stress, and antibiotic treatment. Microarray data sets were obtained from the Gene Expression Omnibus [11]. All available data sets for stress conditions in *E. coli* K-12 MG1655 were evaluated, and 14 data sets were chosen to identify genes with altered transcription under the aforementioned stressors: GSE11041(heat, cold), GSE20305 (heat, cold, oxidative), GSE61736 (cold, oxidative), GSE56133 (oxidative, antibiotics), GSE15534 (heat), GSE40557 (heat), GSE42675 (heat), GSE60522 (nitrosative stress), GSE58176 (oxidative stress), GSE19370 (oxidative stress), GSE57084 (antibiotics), GSE47221(antibiotics), GSE37026 (antibiotics), and GSE10160 (antibiotics). Details of data sets accession numbers, stressor subtypes, and different conditions included in the study are listed in Table 1.

### 2.2. Identification of Differentially Expressed Genes under Selected Stressors

In a given data set, we defined differentially expressed genes (DEGs) as those genes whose transcript levels are statistically significantly higher or lower at a given condition. We used the GEO-embedded tool, GEO2R, which relies on the Benjamini-Hochberg procedure with a false discovery rate > 0.05 and generates the top 250 DEGs in each sample. To further verify the validity of the tool results, we conducted a two-tailed unpaired *t*-test to compare the normalized log_2_ expression value of treated (stress-induced) to untreated (control) samples. Genes with ratios with P values below 0.05 were considered as significantly differentially expressed.

To generate the heatmaps for each stress condition, we used the online heat mapper (http://www.heatmapper.ca/expression, accessed and used on 12 September 2021), which provided hierarchical clustering of top 250 DEGs based on the study/sample included through average linkage as a default setting. We subsequently combined the top 250 up- or downregulated genes in each sample for each stress condition to form a one list of putative significantly differentially expressed genes under each stress condition.

### 2.3. Identification of Genes That Are Differentially Expressed under Multiple Stressors

The Venn diagram online tool (http://bioinformatics.psb.ugent.be/webtools/Venn, accessed on 6 June 2021) was used for combining gene lists for each stressor to determine genes affected by more than one stress.

### 2.4. Generation of Protein–Protein Interaction Networks

The Search Tool for the Retrieval of Interacting Genes/Protein (STRING) database [12] was used for generating a protein–protein interaction network (PPINs) by integrating DEGs common to three or four stress conditions. The tool was used with medium (cutoff score: 0.4) confidence. Each of these networks was exported to Cytoscape 3.8.2. Highly connected nodes in the network representing major sub-clusters were identified using MCODE (Molecular Complex Detection) clustering algorithm [13].

### 2.5. Determination of Functional Categories from Gene Lists

To determine the functional categories of the shared DEG, we retrieved the Uniprot accession numbers of shared DEGs between at least four (as a restrictive threshold) or at least three stressors (as a permissive threshold), based on Venn diagram-based visualization. The corresponding list was used as the input list for the Database for Annotation, Visualization, and Integrated Discovery (DAVID) [14,15].

## 3. Results

### 3.1. Details of Data Sets Used in Our Analysis

During host infection, *E. coli* encounter different sources of stress, such as low gastric pH, sudden neutralization and relatively high intestinal pH, detergent effect of bile salts, nutritional restrictions, and oxidative stress when it passes to the environment. At some points of their life span, they face one or more of these stresses simultaneously and are adapted to change their transcriptional makeup based on the stressor(s) they face.

**Figure 1:**
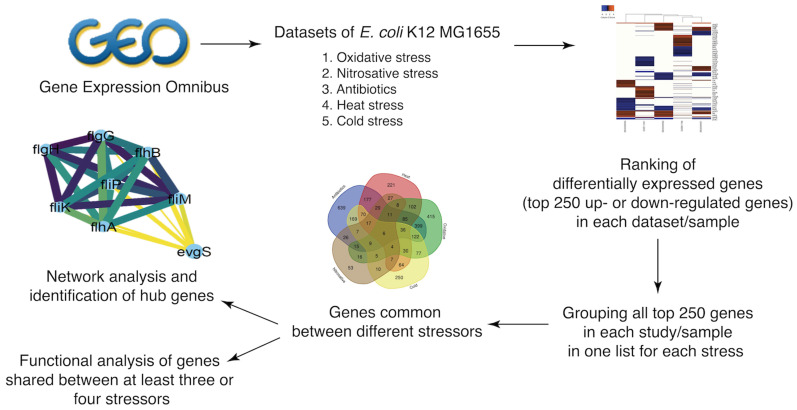
Workflow (flowchart).

Accordingly, we used a computational workflow (Figure 1) to investigate the *E. coli* K-12 MG1655 transcriptome under five different stress conditions, with integrated transcriptional data from multiple data sets (Table 1), as detailed in the Methods section.

### 3.2. Differentially Expressed Genes (DEGs) under Selected Stressors

A typical challenge in such high-throughput studies is how to shortlist transcriptionally altered genes without missing biologically important ones. The reverse challenge is to be overwhelmed with data or to include noise that may mask the signal. As a first step to prioritizing and shortlisting genes, we only included statistically significant DEGs. We then established an arbitrary, rather inclusive, cutoff of the top/bottom 250 DEGs based on their fold change. Through the above-mentioned criteria, we aimed to identify key genes affected by each stressor and common between many stressors. The list of 250 top upregulated and downregulated genes per stressor in each sample are listed in Tables S1–S5.

**Figure 2:**
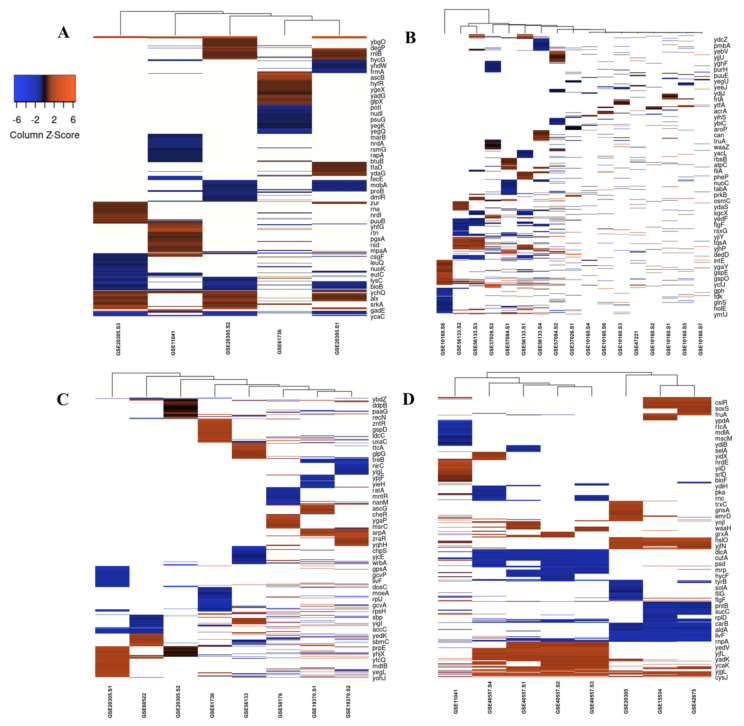
Heatmap and hierarchical clustering of transcriptomic data for all studies for (**A**) cold stress, (**B**) Antibiotics, (**C**) oxidative and nitrosative, and (**D**) heat stress. Only top 250 up- or downregulated genes are included. Shades of orange and blue denote high and low relative expression, respectively.

First, we compared the top 250 DEGs under each stress condition reported by individual studies; some studies involved different samples which we included as well. We observed a noticeable variation among the profiles of DEGs between studies describing the response to each stressor, probably as a result of experimental and technical differences (time of exposure, inoculum size, use of different stressor subtype, e.g., the oxidative response to tellurite and peroxynitrite vs. H_2_O_2_ exposure) (Figure 2A–D). When we combined the list of DEGs retrieved from all included studies (Tables S6 and S7), we identified 1817 upregulated and 1725 downregulated genes between all samples in antibiotics treatment, 883 upregulated and 878 downregulated genes between all samples in cold stress, 894 upregulated and 914 downregulated genes between all samples in heat stress, 250 upregulated and 250 downregulated genes in one sample in nitrosative stress, and 1340 upregulated and 1293 downregulated genes between all samples in oxidative stress.

### 3.3. Identification of Genes Affected by Multiple Stressors

Next, we examined the common DEGs when *E. coli* K-12 MG1655 were subjected to different stressors. The overlap between transcriptional responses to multiple stressors could provide better understanding of general stress response and suggest targets for decreased tolerance towards groups of stressors.

**Figure 3:**
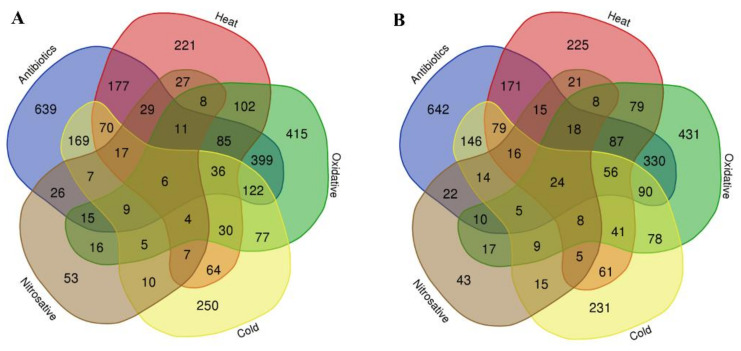
Venn diagram displaying the overlap of (**A**) upregulated and (**B**) downregulated genes within and between the five stress conditions.

We found a significant overlap of up- and downregulated genes within the five stressors. Within our list of upregulated genes, we identified 683 genes that are common between antibiotic treatment and oxidative stress, 431 between antibiotic treatment and heat stress, 436 between antibiotic treatment and cold stress, and 120 between nitrosative and antibiotic treatment stress. Regarding downregulated genes, we identified 620 common genes between antibiotic treatment and oxidative stress, 466 between antibiotic treatment and heat stress, 430 between antibiotic treatment and cold stress, and 124 common downregulated genes between the nitrosative and antibiotic treatment stress (Figure 3A,B).

To this end, we sought to analyze the functional potential of the DEGs shared by different stressors to unveil the functions and pathways playing pivotal roles in stress response. Identification of key functional categories is particularly important for understanding *E. coli* physiology, mechanism of adaptation to different ecological niches, and pathogenicity.

### 3.4. Functional Categories Involved in E. coli Response to Multiple Stressors

**Figure 4:**
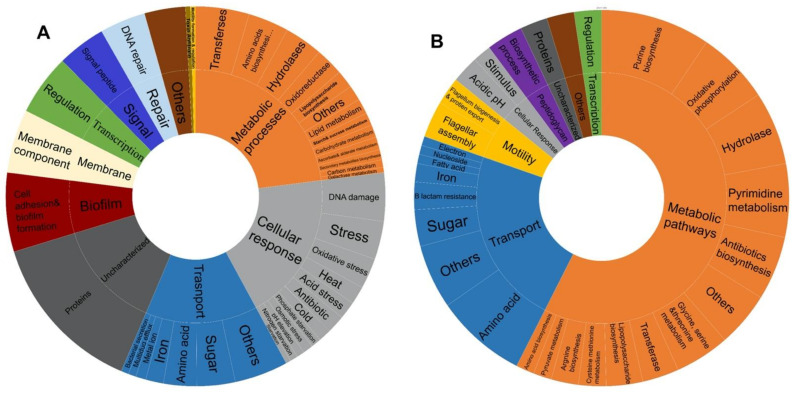
Pie chart displaying the distribution of functional categories among (**A**) upregulated genes, (**B**) downregulated gene shared between at least four stress conditions.

Functional categorization, using DAVID, of the DEGs that are shared between at least four stressors (Table 2 and Table 3) revealed that 26.5% of the identified upregulated genes are involved in survival, general stress response pathways, and response to DNA damage and various stimuli, 15.5% in transport, 12% in various metabolic processes, 12% in biofilm formation, 6 % DNA repair, 5% in transcription regulation, and 1% in motility (Figure 4A). Intriguingly, the analysis delineated 15 genes with uncharacterized functions that are upregulated by at least four out of the five stressors (representing 18% of the total number of upregulated genes). In parallel, the downregulation of various metabolic pathways is a shared feature between the response to all included stressors and constituted 57.5% of the 127 gene expression changes, 22.8% involved in transport, 5.5% in motility, 4% in response to various stimuli, 3% in peptidoglycan biosynthetic processes, and 2.4% in transcription regulation of cellular processes (Figure 4B). Three uncharacterized genes (out of 127), *ymfI*, *ydiJ*, and *yedE*, were found to be downregulated under all stressors except nitrosative stress. Of note, nitrosative stress had generally fewer represented functional categories than other stressors among transcriptionally altered genes (Table 2 and Table 3).

The functional categories of upregulated genes common between at least three stressors are presented in Table 4.

Branched-chain amino acid and phenylalanine ABC transport system (*LivJHMGF*) in *E. coli* K-12 are downregulated. *livM*, *livG*, and *livF* are downregulated in four out of five stressors. *livJ* and *livH* are downregulated in three out of five stressors. *artPIQMJ* genes of *E. coli* encoding a periplasmic arginine transport system are also downregulated. Five genes involved in sugar transport are downregulated in all the five stress conditions vs. one upregulated gene. This is consistent with the observation of the decreased transcription of energy-requiring processes related to growth.

Ten genes involved in biofilm formation and regulation are upregulated, suggesting that biofilm formation can be a consequence of bacterial stress response.

The transcription of the flagellar protein-encoding gene, *flgL*, was increased under heat, cold, oxidative, and antibiotic stress; in contrast, seven flagellar genes were downregulated by the same stressors to conserve resources for cell survival in sustained stress.

### 3.5. Network Analysis of DEGs and Identification of Hub Genes

**Figure 5:**
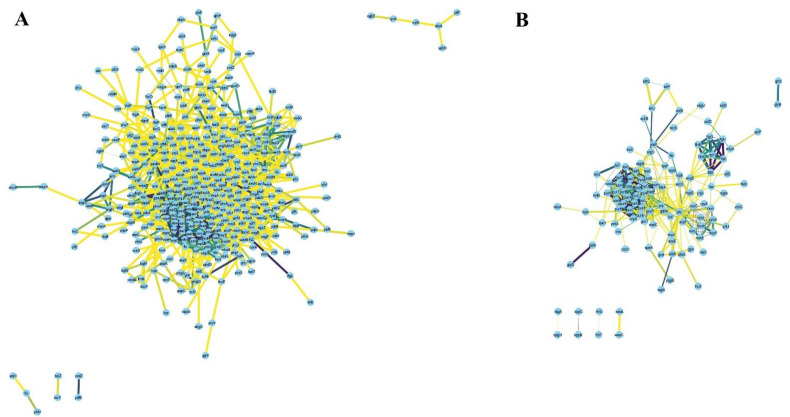
Network of (**A**) 461 upregulated genes in at least three stressors, and (**B**) 127 downregulated in at least four stressors with medium confidence (cut-off score: 0.4).

Based on the functional categories outlined in the previous section, we proceeded to pinpoint “hub genes” that might play key roles within each functional category as a part of *E. coli* response to multiple stressors. Accordingly, we computationally generated a protein–protein interaction network (PPINs) using the STRING database to highlight important nodes with numerous interaction partners and a few major sub-clusters. The network of upregulated genes in at least four stressors showed fewer connections (Supplementary Figure S1) and its main sub-clusters are represented in Supplementary Figure S2; consequently, we generated PPIN of upregulated genes common in at least three stressors (Figure 5A). PPIN of the downregulated genes common in at least four stressors are presented in Figure 5B.

**Figure 6:**
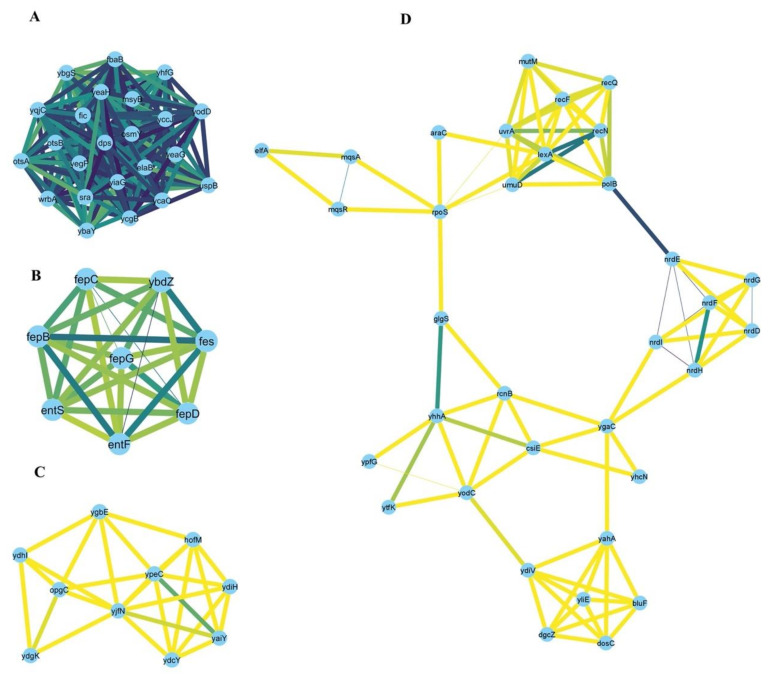
Four main sub-clusters (numbered **A**–**D**) were identified by the MCODE clustering algorithm from the network of the upregulated genes in at least three stressors. Examples of the hub genes are mentioned in the Discussion section.

Our network analysis highlighted the following stress response hubs: for upregulated genes, the following hubs were identified: *elaB*, *dps*, *fbaB*, *fic*, *msyB*, *ostA*, *ostB*, *osmY*, *sra*, *uspB*, *wrbA*, *ybaY*, *ybgS*, *ycaC*, *yccJ*, *ycgB*, *yeaG*, *yeaH*, *yegP*, *yhfG*, *yiaG*, *yodD*, *yqjC (*Figure 6A)*; entF*, *entS*, *fepB*, *fepC*, *fepD*, *fepG*, *fes*, *ybdZ* (Figure 6B)*; hofM*, *opgC*, *yaiY*, *ydcY*, *ydhI*, *ydiH*, *ydgK*, *ygbE*, *yjfN*, *ypeC (*Figure 6C)*; araC*, *bluf*, *csiE*, *dgcZ*, *dosC*, *elfA*, *glgS*, *lexA*, *mqsA*, *mqsR*, *mutM*, *nrdD*, *nrdE*, *nrdF*, *nrdG*, *nrdH*, *nrdI*, *polB*, *rcnB*, *recF*, *recN*, *recQ*, *rpoS*, *umuD*, *urvA*, *yahA*, *ydiV*, *ygaC*, *yhcN*, *yhhA*, *yliE*, *yodC*, *ypfG*, *ytfK* (Figure 6D).

**Figure 7:**
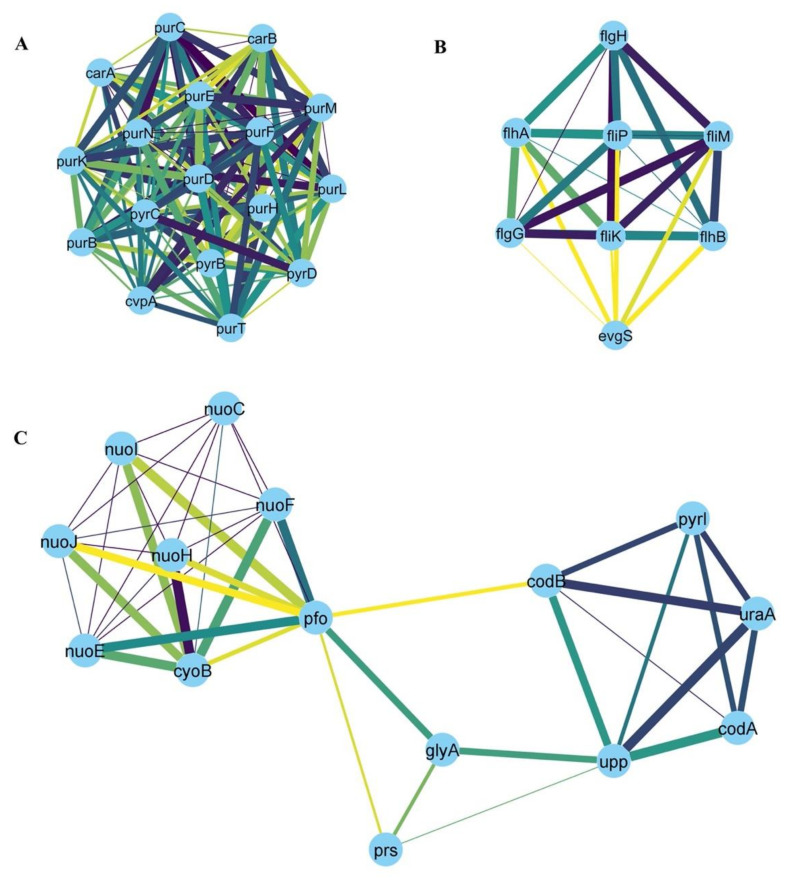
Three main sub-clusters (numbered from **A**–**C**) were identified by the MCODE clustering algorithm from the network of the downregulated genes in at least four stressors. Examples of the hub genes are briefly discussed in the Discussion section.

For downregulated genes, the following hubs were identified: *purF, purD, purN, purT, purL, purM, purK, purE, purC, purB, purH, pryB, pyrC, pyrD, carA, carB, cvpA* (Figure 7A); *flgH, flgG, fliK, fliM, flip, flhA, flhB, evgS* (Figure 7B); *nuoC, nuoE, nuoF, nuoH, nuoI, nuoJ, pfo, prs, codA, codB glyA, pyrI, upp, uraA, cyoB* (Figure 7C).

## 4. Discussion

In this study, we used a systems biology approach by analyzing hundreds of transcriptomic data sets to identify key genes and pathways involved in the stress tolerance of *E. coli* K-12 MG1655, as a model for bacterial response to multiple stressors. Although genes involved in specific stress responses have been frequently studied in *E. coli*, the underlying mechanisms behind global stress response remain to be explored, as only a handful of studies explore multiple stress responses as a complex network, with central proteins controlling underlying mechanisms.

Our approach offers a thorough investigation of transcriptomic data in response to five different stressors: heat, cold, oxidative, and nitrosative stress, along with antibiotic treatment—providing a comprehensive overview of *E. coli* stress response. Our work is a step toward understanding the control of stress response, and toward identifying common gene sets connecting and regulating important biochemical pathways. Consequently, these pathways could be potential drug targets for overcoming antibiotic tolerance or multidrug resistance.

While looking for DEGs under five different stress types, we observed a substantial set of common DEGs. In most cases, all or most genes of an operon clustered together or showed the same expression profile; however, it was also common that some genes within the same operon were not detected in every stress, while their chromosomal neighbors were. This is expected with the use of inclusion/exclusion thresholds for DEGs (e.g., statistical significance thresholds, or a certain percentile rank to include). Among the other reasons are batch effects, inter-experiment variabilities, and the different sensitivities of different probes or primers used for transcript quantification. The variability between media types (e.g., rich LB vs. defined M9 or MOPS media) had different effects on sample clustering depending on the applied stressor. For example, in the heat stress category, samples from the GSE42675, GSE15534, and GSE20305 studies were clustered in the right panel of the heat map. The first two studies shared the same type of medium (M9) and time of exposure (10 min) with a small difference in the applied temperature. Samples from GSE15534 and GSE2030 studies shared the same time of exposure (10 min) and temperature (45 °C), but used different media types (M9 and MOPS, respectively).

In the oxidative stress data sets, samples from studies GSE20305 and GSE61736 were clustered together in the left panel of the heatmap, as they shared the source of oxidative stress (H_2_O_2_) and the same culture medium (MOPS), while GSE56133 sample was clustered with them as a different culture medium was used (LB) with the same type of stress (H_2_O_2_). Among the sets of upregulated genes, six genes were upregulated under all the five studied conditions:

- ***osmB*** encodes an outer membrane lipoprotein, known to be upregulated by osmotic shock and stationary phase [16,17,18]. Although the function of *osmB* is not fully characterized [19], its transcriptional regulation was thoroughly studied and found to depend on the response regulator, RcsB, and its transcription was found to increase following the activation of the RcsCDB phosphorelay [20]. Our analysis indicated that *osmY*, *osmF*, and *osmC* were downregulated under three, two, and one stressor, respectively.
- ***nrdH* and *nrdI*:** NrdH, a gluatredoxin-like protein, serves as the electron donor for NrdEF and is reduced by thioredoxin reductase [21]. *nrdH* is part of the *nrdHIEF* operon, while *nrdEF* encodes class Ib ribonucleotide reductase (RNR), catalyzing the reduction of ribonucleotides (NTP) to deoxyribonucleotides (dNTP), a key reaction for DNA synthesis, replication, and repair. *nrdE* encodes the alpha 2 subunit, harboring the site for nucleotide reduction, and *nrfF* encodes the beta 2 subunit, which contains the metallocofactor required for catalysis at alpha 1 subunit [22]. Our analysis indicated *nrdE*, *nrdF* to be upregulated under at least four stressors. NrdI is a flavodoxin that mediates the generation of the tyrosyl radical cofactor of NrdF. Oxidative stress was reported to induce *nrdHIEF* expression (up to 23.4-fold), suggesting that *E. coli* overexpresses various reductases and electron donors to increase the cell’s free radical scavenging capacity to cope with oxidative stress [23]. Our analysis indicated that this operon was upregulated under oxidative stress, antibiotic treatment, cold, heat, and nitrosative stress.
- ***yqjI* (*nfeR*)** is part of the *yqjH*-*yqjI* operon. *yqjI* encodes a DNA-binding transcription repressor that regulates the expression of the NADPH-dependent ferric siderophore reductase yqjH [24]. YqjH is required for iron homeostasis in *E. coli* and it is also part of the Fur regulon [25]. Our analysis indicated that *yqjH* was upregulated under antibiotic and heat stress. In aerobic conditions, iron is present as insoluble iron hydroxides; consequently, bacteria produce siderophores, which are extracellular ferric chelators, to mobilize iron [26]. Once ferri-siderophores complexes are transferred through membranes to the cytoplasm, they are either degraded by esterase or reduced by ferric siderophore reductases to release ferrous ions [27].
- ***yhcN*** encodes a putative periplasmic protein involved in response to hydrogen peroxide and acid stress. A knockout strain lacking *yhcN* was found to be more sensitive to hydrogen peroxide and was more able to form biofilm than its parental strain [28]. YhcN was first reported to be upregulated in response to cytoplasmic acid stress by Kannan, et al. [29].
- ***ycfJ*** is similar to the *Proteus mirabilis umoD* gene, involved in the flagellar synthesis and cell elongation [30]. Its expression was reported to be induced in biofilm formation, while its null mutant is deficient in biofilm formation [31].

Both *yhcN* and *ycfJ* are upregulated under all the five stressors included in our analysis, including antibiotics. As their roles remain elusive, this observation may help in exploring their molecular functions, as it suggests leads to gene function and heavy involvement in global stress response pathways.

Based on the above findings, we conclude that all five stressors upregulate the transcription of various stress-responsive proteins, such as OsmB, and genes related to various reductases and electron donors, such as the *nrdHIEF* operon. More pathways and functions have been identified by functional analysis of upregulated genes common to at least three stressors (Figure 5A).

Among the sets of downregulated genes, 24 genes were downregulated under all the 5 conditions, 14 of which are involved in purine or pyrimidine biosynthesis. Interestingly, nine of the ten genes involved in the purine biosynthetic pathway (all but *purF*), were directly repressed by the regulator PurR [32,33,34,35,36,37,38,39,40].

The following are the genes involved in purine biosynthesis, specifically in IMP (inosine 5′-monophosphate) biosynthetic pathway from PRPP (5-phosphoribosyl-1-pyrophosphate):

- ***prs*** encodes ribose-phosphate diphosphokinase, which transfers a pyrophosphoryl group from ATP to ribose 5-phosphate, synthesizing PRPP that is utilized in the biosynthesis of purine and pyrimidine nucleotides [41].
- ***purF*** encodes an amidophosphoribosyl transferase, which catalyzes the pathway flux-controlling step in de novo purine biosynthesis. In this reaction, 5-phospho-β-D-ribosyl-amine (PRA) is formed from PRPP and glutamine. It is feedback-regulated by GMP and AMP [42].
- ***purD*** encodes a phosphoribosylamine glycine ligase, the second enzyme in the *de novo* purine biosynthesis pathway. It catalyzes the ligation of glycine PRA to produce 5-phospho-ribosyl-glycineamide (GAR) [43].
- ***purN* and *purT*** encode two different GAR transformylases, with no significant homology; each of them can catalyze the third step in de novo purine biosynthesis, producing 5-phospho-ribosyl-N-formylglycinamide (FGAR). PurN transfers a formyl group from 10-formyl-tetrahydrofolate, while PurT utilizes formate, after the hydrolysis of 10-formyl-tetrahydrofolate by *purU* [44].
- ***purL*** encodes a phosphoribosylformylglycinamide synthetase, which catalyzes the fourth step in the *E. coli* de novo purine biosynthesis pathway. In this reaction, 5-phosphoribosyl-N-formylglycineamidine (FGAM) is formed from FGAR, glutamine, and ATP [45].
- ***purM*** encodes phosphoribosylformylglycinamidine cyclo-ligase (AIR synthetase), which catalyzes the fifth step in the de novo purine biosynthesis pathway [46], in which 5-amino-1-(5-phospho-D-ribosyl)imidazole (AIR) is formed from FGAM and ATP [47].
- ***purC*** encodes a phosphoribosylaminoimidazole-succinocarboxamide synthase, which catalyzes the formation of 4-(N-succinylcarboxamide)-5-aminoimidazole ribonucleotide (SAICAR) from CAIR [48]. This is similar to another reaction catalyzed by PurA (adenylosuccinate synthetase) that also utilizes aspartate and a ribonucleoside triphosphate [49]. Our analysis indicated that *purA* was downregulated under antibiotic treatment and heat stress.
- ***purB*** encodes adenylosuccinate lyase, which catalyzes two reactions in de novo purine nucleotide biosynthesis. It converts 5-aminoimidazole-4-carboxamide SAICAR to ribonucleotide (AICAR) and catalyzes the breakdown of adenylosuccinate to AMP [38].
- ***purH*** encodes the bifunctional AICAR transformylase/IMP cyclohydrolase, which catalyzes the last two steps of the de novo purine biosynthetic pathway converting AICAR to IMP [50].

Two extra genes involved in the purine biosynthetic pathway, *purK* and *purE*, were upregulated under four stressors (heat, cold, nitrosative stress, and antibiotic treatment) but not under oxidative stress.

Additionally, two genes are indirectly related to purine metabolism as they encode the enzymes that catalyze the formation of 5,10- methylenetetrahydrofolate (5,10-mTHF), which acts as a methyl group donor in the biosynthesis of various cellular components, including purines and methionine:

- ***glyA*** encodes a serine hydroxymethyltransferase, which catalyzes the conversion of serine to glycine through forming 5,10-mTHF. It is activated by MetR, and repressed by MetR and PurR [51].
- ***gcvT*** is part of *gcvTHP* operon. It encodes the T-protein in the glycine cleavage system, which catalyzes glycine degradation and the formation of 5,10- methylenetetrahydrofolate 5,10-mTHF. The other two genes encode GcvH, or H-protein, and GcvP, or P protein [52,53,54]. Expression of the glycine cleavage enzyme system is induced by glycine, activated by GcvA, and repressed by PurR and GcvA [55,56]. Both *gcvH* and *gcvP* in our analysis were downregulated under three and four stressors, respectively, indicating the many-sided roles of GcvTHP beyond amino acid metabolism. GcvA is also the activator of *gcvB*. Both were reported to be related to stress response. The *hdeAB* operon, which encodes chaperone-like functions, was reported to be activated by GcvB and repressed by GcvA. Both HdeA *and* HdeB protect periplasmic proteins from aggregation by acid stress [57]. *gcvB* encodes a small regulatory RNA. *gcvB* knockout mutant was found to be more sensitive to oxidative stress and accumulate more endogenous reactive oxygen species than wild type. The role of *gcvB* in oxidative stress was also found to be conferred by increasing OxyR expression [58]. These findings suggest that *gcvB* and *gcvA* products allow *E. coli* to survive in presence of both oxidative stress and low pH. Our findings expand their regulatory functions to cold, heat, and antibiotic treatment.

Among downregulated genes, four are involved in the pyrimidine biosynthetic and salvage pathway:

- ***pyrC*** encodes a dihydroorotase, which catalyzes the third reaction in the de novo pyrimidine biosynthesis pathway. By looking for other genes involved in pyrimidine metabolism in our data, we found seven more genes to be downregulated: *pyrB*, *pyrD*, and *pyrI* were downregulated under four stressors while *pyrE*, *pyrF*, *pyrG*, and *pyrL* were downregulated under two stressors.
- ***carA*** is part of the *carAB* operon. It encodes the amidotransferase component, CarA, while the CarB subunit is the synthetase component of the carbamoylphosphate synthetase, involved in L- arginine synthetic pathway, along with the de novo uridine monophosphate (UMP) biosynthetic pathway (part of pyrimidine biosynthesis). We found *carB* to be downregulated under four stressors.
- ***codB*** and ***codA*** form the *codBA* operon. CodB is a cytosine permease, which brings cytosine into the cell [59], while CodA is a cytosine deaminase (CDA), which catalyzes cytosine deamination into uracil. It is one of the enzymes in the pyrimidine salvage pathway, permitting the cell to utilize cytosine for pyrimidine nucleotide synthesis [60,61].

The downregulation of purine and pyrimidine metabolism, which we repeatedly observed here, has previously been reported as a response to acid exposure (pH 3.6) in *E. coli* O26:H11, which induces the accumulation of certain metabolites and enzymes involved in purine metabolism [62]. Pyrimidine levels were reported to decrease to 59%, after 20 min of HOCl stress, relative to the reference levels of untreated cells [63]. The biosynthesis of nucleotides, such as cytidine and uridine, was decreased after the combined ultrasound and mild acidic electrolyzed water treatment or electrolyzed water treatment alone, as well as the pool concentration of pyrimidines in the planktonic *E. coli* cells. Simultaneously, ribose-5-phosphate, the precursor for nucleotide synthesis, was downregulated in planktonic *E. coli* cells, after electrolyzed water treatment, indicating a depression in nucleotide biosynthesis [64]. In addition, levels of adenosine monophosphate (AMP) and uridine monophosphate (UMP), which are products of nucleotide degradation, were elevated in pulsed light treated *E. coli*, indicating DNA damage as well as blockage of RNA synthesis [65].

In addition to nucleotide metabolism-related genes, two genes are part of electron transport chain complexes:

- ***cyoB*** encodes subunit I of the cytochrome bo_3_ complex. It is a part of the *cyoABCDE* operon. Our analysis indicated that *cyoA*, *cyoC*, *cyoD*, and *cyoE* were downregulated under two out of five stressors.
- ***nuoC*** is part of the *nuoABCEFGHIJKLMN* operon, representing the 13 subunits of NADH ubiquinone oxidoreductase. Our analysis detected the downregulation of *nuoE*, *nuoF*, *nuoH*, *nuoI*, and *nuoJ* under four stressors, and of *nuoB*, *nuoG*, *nuoK*, *nuoL*, *nuoM*, and *nuoN* under three stressors, while *nuoA* was detectably downregulated under two stressors.

*E. coli* was previously reported to adapt to stressors affecting cell envelope integrity through the Cpx-mediated repression of the *nuo* and *cyo* operons. The *nuo* and *cyo* transcriptional downregulation suggested that those proteins are toxic in the presence of envelope stress [66]. The membrane-bound systems for proton and electron transport Nuo (the NADH dehydrogenases I and II) and Cyo (cytochrome o oxidase) were downregulated at high pH [67]. Our analysis detected the downregulation of these systems, or part thereof, under all five investigated stressors.

Other genes that were downregulated under all studied stressors are:

- ***fadL*** encodes the component of a channel involved in the import of long-chain (C12-C18) fatty acids (LCFA) across the bacterial outer membrane [68]. It is part of the fatty acid-degrading (fad), regulon which includes eight more genes involved in fatty acids catabolism [69]. Our analysis indicated that *fadD*, *fadE*, *fadI*, and *fadR* were downregulated under at least two stressors, suggesting that, during stress, *E. coli* downregulates the uptake of exogenous fatty acids along with its metabolism and degradation which generates various reduced cofactors. As previously reported, LCFA degradation generates oxidative stress with high levels of reactive oxygen species [70,71].
- ***cvpA*** encodes colicin V production protein [72] and is located directly upstream of *purF* and repressed by PurR, the main repressor of purine synthetic pathway [73]. Its deletion mutant in EHEC was highly sensitive to bile and was deemed important in cell envelope homeostasis in response to stressors, such as deoxycholate bile salt [74].
- ***ybhC*** encodes an outer membrane lipoprotein [75]. Genome-wide screening of an ASKA library found that overexpression of *yhbC* leads to an increase in the minimum inhibitory concentration (MIC) of hydrogen peroxide by more than three folds [76].
- ***gtrB* (*yfdH*) and *gtrS* (*yfdI*)** are part of the *yfdGHI* operon. Our analysis indicated that *yfdG* was downregulated under two stressors. *yfdG*, *yfdH*, and *yfdI* are homologous to the type IV O antigen modification genes (*gtrAIV*, *gtrBIV*, and *gtrIV*) in the genome of *Shigella flexneri* NCTC 8296 [77].
- ***rnb*** encodes the ribonuclease II enzyme (RNase II), involved in the specific degradation of mRNA in the 3′ to 5′ direction [78].

Network analysis identified upregulated hub genes, which included seven genes involved in biofilm formation (*dosC*, *yhcN*, *yodD*, *dgcZ*, *mqsR*, *elfA*, and *glgS*), suggesting that stress response can lead to biofilm formation. Hub genes also include genes involved in ferri-enterobactin transport (*fepB*, *fepC*, *fepD*, and *fepG*) and utilization (*fes*). *entF* was upregulated under three stressors (antibiotics, heat, and oxidative stress). It is part of the *entABCDEF* operon. Our analysis indicated that *entA, entB, entC, entD,* and *entE* were upregulated in antibiotic and oxidative stress. This operon is involved in enterobactin biosynthesis, which is a well-known siderophore. This highlights the importance of iron availability for *E. coli* to cope with different stressors.

Downregulated hub genes include seven genes of flagellar biogenesis and assembly (*flgH*, *flgG*, *fliK*, *fliM*, *flip*, *flhA*, and *flhB*). A strong relation between motility suppression and high pH was previously reported [67]. Here, we believe that most of those genes are downregulated to preserve energy when *E. coli* is exposed to cold, heat, oxidative stress, or antibiotic treatment.

In general, the purpose of stress response to concentrate cellular resources on survival, as opposed to growth/multiplication. So, based on our analysis and several prior studies, we conclude that major stress responses involve maintaining or upregulating genes related to survival, biofilm formation, and stress response, while downregulating non-essential energy-requiring processes related to growth and replication. Overall, the globally upregulated pathways we identified for stress responses fall into three major categories: (i) cellular stress response, (ii) DNA repair, and (iii) cell adhesion and biofilm. On the other hand, the main downregulated pathways we identified for stress responses are: (i) de novo purine and pyrimidine biosynthesis pathways, salvage, and uptake, (ii) oxidative phosphorylation/aerobic respiration, and (iii) motility.

Of note, while this manuscript was in final preparation, a publication using a similar approach reported a set of genes and networks involved in a collection of stressors [8]. The studied stressors were nutrition limitations (rich M9 and poor M9), acidic stress (pH_5_), antibiotics (trimethoprim (TMP) and chloramphenicol (CAM)), and oxidative stress conferred by growth in low oxygen (LOX) environments, yet the main emphasis of the paper was on transcription factors and regulatory networks. Although different computational approaches were used, the work agrees with ours in the following: nutrition limitations in *E. coli* indicated by the poor M9 medium were enriched in downregulation of the flagellar genes and upregulation of CsgD, a biofilm master regulator. TMP induced SOS response and cellular response to various stimuli. The SOS response inhibits cell division upon exposure to TMP by upregulating *sulA*. *sulA* was also upregulated in all five stressors in our analysis. TMP also induced upregulation of genes *csgD*, *csgE,* and *csgF* of the *csgDEFG* operon as well as *csgB* of the *csgBA* operon. In our study *csgF* and *csgB* were upregulated in three stressors, *csgG* was upregulated in two stressors, and heat stress upregulated *csgA, csgD,* and *csgE.* Those two operons involved in biofilm formation confer protection to bacteria against antibiotics and stressful conditions. Genes in the main five sub-networks generated in that study belonged to flagellar assembly, energy metabolism, SOS response and DNA repair, RNA binding proteins, and biosynthesis of amino acids and secondary metabolites. This is consistent with our finding as biofilm formation, DNA repair, and the bacterial response to stress and to various stimuli were enriched in upregulated genes. Functions such as energy-requiring metabolism, purine and pyrimidine biosynthesis, flagellar assembly, biogenesis, and export were enriched in downregulated genes. Individual genes in those main sub-networks were represented in our hub up or downregulated genes: nineteen genes of energy metabolism (*elaB*, *dps*, *fbaB*, *fic*, *msyB*, *osmY*, *sra*, *wrbA*, *ybaY*, *ybgS*, *ycaC*, *yccJ*, *ycgB*, *yeaG*, *yeaH*, *yegP*, *yiaG*, *yodD*, and *yqjC*; Figure 6A), four genes of DNA repair (*lexA*, *recF*, *recN*, and *recQ*; Figure 6D) and four genes of amino acids and secondary metabolites biosynthesis (*entF*, *fepC*, *fepG*, and *fes;* Figure 6B) were in hub upregulated genes; six flagellar genes (*flgH*, *flgG*, *fliK*, *fliM*, *flip*, and *flhB;* Figure 7B) and one gene of amino acids and secondary metabolites biosynthesis (*cyoB;* Figure 7C) were in hub downregulated genes.

Intriguingly, PurR was identified as a major regulator that is related to several of the genes and pathways detected in our analysis. PurR is considered one of the general transcription factors (TFs) as it controls the expression of genes related to connecting reactions as well as genes with diverse functional categories [79]. PurR function and size are considered limited when compared to other general TFs in *E. coli*, such as Crp [80], Fnr [81], and Lrp [82]. In *Bacillus* subtilis, the nucleotide messengers ppGpp and pppGpp (collectively (p)ppGpp) were reported to bind to PurR, therefore increasing its DNA binding and enhancing the downregulation of PurR-regulated genes involved in purine biosynthesis during nutrient starvation. This suggests that purine biosynthesis is repressed by PurR to cope with unfavorable conditions during starvation [83]. PurR has not been previously reported to be related to stress in *E. coli*, yet in our analysis, most of the downregulated genes are known to be repressed by PurR, and the purine and pyrimidine biosynthesis, salvage, and uptake pathways were highly represented among downregulated data. Taken together, these findings suggest that PurR has a critical role in balancing the metabolism *E. coli* under different types of stress.

## 5. Conclusions

In summary, this study aimed at using a systems approach, through integrating several data sets, to determine genes that are involved in the response to multiple stresses. Because multiple array platforms were used, we adopted a percentile rank-based approach to pick significantly altered genes, and we gave emphasis to gene sets or pathways rather than individual genes, as gene-level analysis is sensitive to noise, batch effects, and experimental variations.

The strengths of our approach are that it is unbiased and statistically robust and that it relies on network enrichment to overcome the false positive inclusion or false negative exclusion of individual genes. Most of the statistically significant results were also biologically relevant, or at least biologically explainable. For example, the downregulation of non-essential functions under stress and the upregulation of genes necessary for survival are consistent with an “emergency response” in which the cell manages its resources carefully. Meanwhile, a few genes remain unassigned with any functions and will be subject to further experimental validation. Like all computational approaches, the strategy followed here remains hypothesis generating rather than confirmatory and certainly requires future validation via experiments relying, for example, on gene deletion and insertion (loss and gain of function, respectively). In addition, like many high-throughput analyses, the study cannot establish causation, and some of the observed variations might be consequential while some may be collateral. In addition, the approach may overlook genes that may be biologically relevant but fall out of the statistical thresholds for one reason or another. This is exemplified by the absence of some operon members even when most of the operon is selected among stress response genes.

## Figures and Tables

**Table 1:** Details of stress conditions used in each data set.

Stressor	Subtype	Media
**Heat**	GSE15534 (45 °C 10 min)	M9 complete medium
	GSE40557 (58 °C f = 2, 58 °C f = 3, 60 °C f = 3, 71 °C)	Brain Heart Infusion (BHI)
	GSE42675 (42 °C 10 min)	M9 minimal medium
	GSE11041 (46 °C till 1 × 10^7^ CFU/mL)	Tryptone soya broth (TSB)
	GSE20305 (45 °C 10 min)	Modified morpholinopropane sulfonate (MOPS) minimal medium
**Oxidative stress**	GSE20305 (H_2_O_2_ 10 min and 90 min)	Modified MOPS minimal medium
	GSE61736 (H_2_O_2_ till OD600 0.3–0.4)	MOPS medium
	GSE56133 (10 uM H_2_O_2_ 60 min)	Luria-Bertani (LB) broth
	GSE58176 (Tellurite 0.5 µg/mL 15 min)	LB broth
	GSE19370 (300 μM peroxynitrite 5 min, 300 μM H_2_O_2_ 5 min)	Defined media containing 2 mM glycerol
**Cold**	GSE 11041 (15 °C till 1 × 10^7^ CFU/mL)	TSB
	GSE61736 (15 °C 4 h)	LB broth
	GSE20305 (16 °C 10 min, 30 min and 90 min)	Modified MOPS minimal medium
**Nitrosative**	GSE60522 (Dipropylenetriamine (DPTA) 10 min)	MOPS minimal medium
**Antibiotics**	GSE56133 (Ampicillin 1 h, Gentamycin 1 h, Kanamycin 1 h, Norfloxacin 1 h)	LB broth
	GSE57084 (Enorfloxacin, Tetracycline)	Fresh Evans medium
	GSE47221 (Amoxicillin)	Fresh Evans medium
	GSE37026 (Colicin 30 min and 60 min)	LB broth
	GSE10160 (Cefsulodin 10 μg/mL 5 min, 20 min and 40 min, Cefsulodin 60 μg/mL 10 min, Mecillinam 0.03 μg/mL 5 min, 20 min and 40 min, Mecillinam 0.3 μg/mL 60 min)	LB broth

**Table 2:** Upregulated gene functions in multiple conditions (at least four stressors).

Functional Category	Function Type	N Genes	Names of Genes	Stress				
				Antibiotic	Oxidative	Cold	Heat	Nitrosative
**Metabolic processes**	Transferses	2	*tusb*, *rlmB*	×	×	×	×	
	Hydrolases	2	*ycaC*, *yliE*	×	×	×	×	
	Amino acids biosynthesis and metabolism	2	*ilvM*, *fbaB*	×	×	×	×	×
	Oxidoreductase	1	*Ndh*	×	×	×		×
	Lipid metabolism	1	*pgpC*	×		×	×	×
	Starch and sucrose metabolism	1	*treF*	×	×	×	×	
	Secondary metabolites biosynthesis	1	*cadA*	×	×	×	×	
**Cellular response**	DNA damage	8	*ycgB*, *blc*, *gadX*, *gadW*, *yqjI*, *iraD*, *sulA*, *ybaV*	×	×	×	×	×
	Stress	4	*rmf*, *uspG*, *mqsA*, *bolA*	×	×	×	×	×
	Oxidative stress	1	*grxA*	×	×		×	×
	Acid stress	5	*slp*, *ydeP*, *ygaC*, *ycgZ*, *mgrB*	×	×	×	×	
	Osmotic stress	1	*osmB*	×	×	×	×	×
	Phosphate starvation	1	*psiE*	×		×	×	×
	Heat	1	*ldhA*	×	×		×	×
	Nitrogen starvation	1	*yeaG*	×		×	×	×
**Trasnport**	Amino acid	3	*leuE*, *gltP*, *alaE*	×	×	×	×	×
	Sugar	1	*bglF*	×	×	×	×	
	Iron	2	*fecI*, *fepD*	×	×	×	×	×
	Metal ion	2	*corA*, *zntA*	×	×	×	×	×
	Bacterial secretion	1	*gspO*	×	×	×		×
	Multidug efflux	1	*emrD*	×	×		×	×
	Others	3	*tehA*, *ybhS*, *ytfL*	×	×	×		×
**Cell adhesion and biofilm formation**		10	*ychH*, *yhcN*, *yodD*, *bssS*, *ycfJ*, *ymgA*, *dgcZ*, *cnu*, *tomB*, *ybfG*	×	×	×	×	×
**Transcription regulation**		4	*eutR*, *yddm*, *zntR*, *dsdC*	×	×	×	×	×
**DNA repair**		5	*recF*, *nrdE*, *nrdF*, *nrdH*, *nrdI*	×	×	×	×	
**Motility**	Formation and regulation	1	*flgL*	×	×	×	×	
**Others**		3	*bluf*, *sra*, *essQ*	×	×	×	×	
**Uncharacterized proteins**		15	*yqfA*, *yhfG*, *yhhA*, *ybgS*, *arpA*, *yqaE*, *yfdY*, *yaiY*, *yebE*, *ydiE*, *yjcB*, *yiiX*, *ycjF*, *yihF*, *yidX*	×	×	×	×	×

An × denotes that half or more of the genes within a functional category are differentially expressed.

**Table 3:** Downregulated gene functions in multiple conditions.

Functional Category	Function Type	N Genes	Names of Genes	Stress				
				Antibiotic	Oxidative	Cold	Heat	Nitrosative
**Transport**	B lactam resistance	3	*oppB*, *oppC*, *ampG*	×	×		×	×
	Electron	1	*rsxC*	×	×		×	×
	Iron	2	*fecC*, *efeo*	×	×	×	×	×
	Nucleoside	1	*tsx*	×		×	×	×
	Fatty acid	1	*fadL*	×	×	×	×	×
	Sugar	4	*gatA*, *fruB*, *ptsG*, *mglA*	×	×	×	×	×
	Amino acid	10	*livM*, *livG*, *livF*, *artQ*, *artp*, *artJ*, *lysP*, *plaP*, *pheP*, *codB*	×	×	×	×	
	Others	7	*xanP*, *uraA*, *yeiB*, *thiQ*, *thiP*, *potB*, *potD*	×	^a^ ×	×	×	×
**Metabolic pathways**	Purine biosynthesis	12	*purD*, *purL*, *purB*, *purH*, *purM*, *purN*, *prs*, *purE*, *purT*, *purK*, *purF*, *purC*	×	×	×	×	×
	Antibiotics biosynthesis	7	*accC*, *gph*, *aceE*, *icd*, *ilvC*, *gcd*, *dapB*	×	×	×	×	
	Pyrimidine metabolism	8	*pyrB*, *pyrD*, *pyrI*, *carB*, *carA*, *upp*, *pyrC*, *coda*	×	×	×	×	×
	Argnine biosynthesis	3	*argD*, *gdhA*, *alaA*	×	×	×	×	
	Glycine, serine and threonine metabolism	6	*thrA*, *thrB*, *gcvT*, *gcvP*, *lysC*, *glyA*	×	×	×	×	
	Cysteine methionine metabolism	3	*ynjE*, *metE*, *metC*	×		×	×	×
	Amino acid biosynthesis	2	*trpE*, *cysJ*	×	×	×	×	×
	Lipopolysaccharide biosynthesis	4	*eptC*, *waaL*, *waaC*, *lpxH*	×	×		×	×
	Pyruvate metabolism	2	*pfo*, *aldA*	×	×	×	×	×
	Transferase	4	*hsdM*, *gtrB*, *lipB*, *opgH*	×	×	×	×	×
	Hydrolase	8	*mgtA*, *frmB*, *ydcP*, *hypB*, *ybhC*, *yliE*, *ravA*, *rnb*	×	×	×	×	×
	Oxidative phosphorylation	8	*atpF*, *nuoJ*, *nuoC*, *nuoE*, *nuoF*, *nuoI*, *nuoH*, *cyoB*	×	×	×	×	
	Others	6	*speA*, *bioD*, *pntA*, *gatD*, *ycaO*, *hypD*	×	×	×	×	
**Cellular Response**	Stimulus	2	*tsgA*, *borD*	×	×	×	×	×
	Acidic pH	3	*evgS*, *yagU*, *yqgB*	×	×	×	×	
**Motility**	Flagellum biogenesis and protien export	3	*fliP*, *flhA*, *flhB*	×	×	×	×	
	Flagellar assembly	4	*fliM*, *flgH*, *flgG*, *fliK*	×	×	×	×	
**Peptidoglycan**	Biosynthetic process	4	*dacA*, *mipA*, *lpoA*, *murI*	×	×	×	×	×
**Transcription**	Regulation	3	*fis*, *mprA*, *suhB*	×	×	×	×	×
**Others**		3	*cvpA*, *gtrS*, *yeiP*	×	×	×	×	×
**Uncharacterized**	Proteins	3	*ymfI*, *ydiJ*, *yedE*	×	×	×	×	

An × denotes that half or more of the genes within a functional category are differentially expressed. ^a^ × For oxidative 3 out of 7 genes are upregulated.

**Table 4:** Upregulated gene functions in multiple conditions (at least three stressors).

Function	Function Type	N Genes	Name of Genes
**Metabolic processes**	Transferses	21	*tusb*, *rlmB*, *yjgX*, *ydiU*, *elaA*, *yafK*, *tusE*, *ldtc*, *alaC*, *trmN*, *opgE*, *tdcD*, *wecH*, *opgC*, *yjaB*, *fic*, *lnt*, *maa*, *yafE*, *rlmE*, *rlmG*
	Hydrolases	15	*ycaC*, *yliE*, *yfcI*, *yadD*, *sixA*, *casE*, *ygbF*, *glpG*, *rnd*, *yhjJ*, *yahA*, *cdd*, *dbpA*, *phoA*, *fes*
	Amino acids biosynthesis and metabolism	16	*ilvG*, *metA*, *gltA*, *asnA*, *ilvC*, *tdcG*, *argI*, *argF*, *argH*, *ilvM*, *acnA*, *fbaB*, *puuB*, *puuA*, *eutQ*, *yhfx*
	Oxidoreductase	11	*nrdG*, *nrdD*, *dadA*, *mhpB*, *dmsC*, *torZ*, *dusC*, *nirB*, *qorA*, *ndh*, *hcr*
	Lipopolysaccharide biosynthesis	7	*wcaA*, *waaZ*, *wcaF*, *wzxC*, *wzzB*, *wcaD*, *wcaE*
	Lipid metabolism	6	*yihG*, *pgpC*, *clsC*, *yegS*, *fadD*, *yiiD*
	Ascorbate and aldarate metabolism	5	*garD*, *gudD*, *lgoD*, *sgbE*, *lyxK*
	Carbohydrate metabolism	5	*fsaB*, *araC*, *fucR*, *mlc*, *glmS*
	Starch and sucrose metabolism	5	*glgA*, *amyA*, *bglB*, *treF*, *pgm*
	Secondary metabolites biosynthesis	4	*cadA*, *entF*, *cysN*, *ubiX*
	Carbon metabolism	3	*acs*, *gntK*, *mqo*
	Galactose metabolism	2	*dgoK*, *ebgC*
	Other pathways	6	*ybdZ*, *thiC*, *pyrF*, *atpC*, *torY*, *hofM*,
**Cellular response**	DNA damage	19	*ycgB*, *blc*, *gadX*, *gadW*, *yqjI*, *iraD*, *sulA*, *ydjM*, *sbmC*, *yidQ*, *yqiJ*, *yedV*, *betT*, *elaB*, *yhcF*, *rcnB*, *dinD*, *yedk*, *dinF*
	Stress	15	*rmf*, *uspG*, *mqsA*, *bolA*, *cbpA*, *cbpM*, *yjbJ*, *uspB*, *nemR*, *rclR*, *rclB*, *cpxP*, *pspG*, *rpoS*, *srkA*
	Oxidative stress	12	*grxA*, *msrA*, *wrbA*, *degP*, *sufA*, *clpA*, *yhbO*, *rseC*, *soxR*, *sufE*, *yfcG*, *ytfK*
	Antibiotic	7	*bcr*, *entS*, *mdtO*, *ydaC*, *yibA*, *yojI*, *ymdB*
	Acid stress	7	*slp*, *ydeP*, *ygaC*, *iraM*, *frc*, *ycgZ*, *mgrB*
	Heat	7	*ybeD*, *rpoH*, *htpG*, *hspQ*, *eutD*, *hslJ*, *ldhA*
	Cold	5	*ydfK*, *ynaE*, *cspF*, *cspD*, *cspG*
	Osmotic stress	4	*otsB*, *otsA*, *osmB*, *osmY*
	Phosphate starvation	4	*psiE*, *waaH*, *appY*, *appA*
	Nitrogen starvation	3	*ycjX*, *yeaH*, *yeaG*
	pH elevation	3	*gadB*, *adiC*, *gadA*
	Starvation	2	*gpp*, *dps*
**Trasnport**	Amino acid	13	*leuE*, *gltP*, *alaE*, *ydgI*, *yehX*, *yifK*, *potF*, *proY*, *proX*, *artJ*, *ydjN*, *proP*, *yhdW*
	Sugar	15	*bglF*, *lgoT*, *nanT*, *kdgT*, *srlA*, *ascF*, *fruA*, *araF*, *xylH*, *frvB*, *gntP*, *alsA*, *yicJ*, *ytfT*, *glvC*
	Iron	7	*fecI*, *fecR*, *fiu*, *fepC*, *fepB*, *fepG*, *fepD*
	Metal ion	4	*corA*, *zntA*, *mntH*, *mgtA*
	Bacterial secretion	3	*yidC*, *gspK*, *gspO*
	Multidug efflux	3	*emrD*, *mdtJ*, *mdtI*
	Others	21	*tehA*, *ybhS*, *mlaE*, *yhbE*, *yhjD*, *yjhF*, *yfdV*, *ygaY*, *ycgH*, *araJ*, *yphD*, *hsrA*, *garP*, *nepI*, *cusB*, *adeP*, *yidK*, *yhhQ*, *mscM*, *ytfL*, *phoE*
**Membrane Component**		25	*ypjA*, *ppdD*, *nfrB*, *yaiO*, *wzyE*, *yedR*, *yfjD*, *ygbE*, *yhdU*, *fliR*, *yfbV*, *fxsA*, *yqiK*, *yqjE*, *alx*, *ycdZ*, *ydhI*, *ybhL*, *yeiH*, *yohJ*, *yhfL*, *yjiJ*, *creD*, *ychE*, *ydgK*
**Cell adhesion and biofilm formation**		31	*dosC*, *ychH*, *yhcN*, *yodD*, *bssS*, *ycfJ*, *ymgA*, *dgcZ*, *cnu*, *tomB*, *ybfG*, *tqsA*, *csgF*, *ymgC*, *ariR*, *mqsR*, *csgB*, *bhsA*, *sdiA*, *yjaA*, *yghO*, *fimG*, *elfA*, *sfmD*, *ydeR*, *glgS*, *yjcZ*, *yfcU*, *ydeT*, *fimD*, *ybgQ*
**Transcription regulation**		24	*csiE*, *dctR*, *nsrR*, *hcaR*, *stpA*, *arsR*, *soxS*, *sxy*, *hyfR*, *yidL*, *norR*, *rof*, *yhfY*, *yieP*, *greA*, *chaB*, *ydjF*, *yiaG*, *eutR*, *yddm*, *zntR*, *dsdC*, *Crl*, *ykgA*
**Signal peptide**		19	*yibG*, *ycbK*, *rzpQ*, *yncD*, *ybbC*, *sslE*, *ydbL*, *yjfY*, *ymgD*, *ybaY*, *ygdI*, *ypfG*, *eco*, *ypeC*, *yqjC*, *ytfJ*, *yjfN*, *ykfB*, *ybcW*
**DNA repair**		18	*recF*, *yegP*, *umuD*, *mutM*, *uvrA*, *phr*, *lexA*, *recQ*, *recN*, *polB*, *alkA*, *yebG*, *nrdE*, *nrdF*, *nrdI*, *nrdH*, *yhcG*, *prlC*
**Motility**	Formation and regulation	2	*flgL*, *ydiV*
**Toxin-Antitoxin**		2	*hokD*, *higA*
**Others**		16	*infA*, *rdlD*, *rybB*, *ryhB*, *rttR*, *insZ*, *msyB*, *TfaS*, *ftsA*, *tfaD*, *bluf*, *sra*, *rsxA*, *ybaV*, *essQ*, *essD*
**Uncharacterized proteins**		64	*aroM*, *yqfA*, *yhfG*, *yhhA*, *ybgS*, *arpA*, *yqaE*, *yfdY*, *yaiY*, *yebE*, *ydiE*, *yjcB*, *yiiX*, *ycjF*, *yihF*, *yidX*, *yccJ*, *yifE*, *YheO*, *yffB*, *ydbA*, *ygeN*, *yfaH*, *yrhA*, *yacL*, *ygeQ*, *YifN*, *YjhE*, *YkiA*, *yehH*, *yehQ*, *ycgX*, *yjfK*, *yhiJ*, *yidB*, *ymfD*, *yddH*, *ydeJ*, *ynbE*, *yrbL*, *tpr*, *ycbJ*, *yhcO*, *sgcQ*, *yfbP*, *yaeH*, *ydiH*, *yecT*, *yfbN*, *ygbA*, *yodC*, *tfaP*, *ytfI*, *rem*, *yagN*, *yfdT*, *yffL*, *yggI*, *yfeS*, *ymgG*, *ydcy*, *ymjA*, *yqeB*, *yccM*

## Data Availability

Data tables are available in the Supplementary Material.

## Supplementary Materials

The following supporting information can be downloaded at: https://www.mdpi.com/article/10.3390/microorganisms10091793/s1, Figure S1: Network of (A) 83 upregulated genes in at least four stressors with medium confidence (cut-off score: 0.4); Figure S2: The main sub-clusters of the upregulated genes in at least four stressors network; Tables S1–S5: Top 250 up- or downregulated genes in each study/sample in heat stress, oxidative stress, cold stress, nitrosative stress, and antibiotic stress, respectively; Table S6: Combined list of upregulated in all samples of each stress; Table S7: Combined list of downregulated genes in all samples of each stress.
