# Supplementary material for: Gene Networks and Pathways Involved in Escherichia coli Response to Multiple Stressors
Source: Microorganisms. 2022 Sep 6;10(9):1793. doi: 10.3390/microorganisms10091793 (PMC9501238; doi:10.3390/microorganisms10091793)
Supplement: Supplementary file 1 [file microorganisms-10-01793-s001.zip › microorganisms-1877155-supplementary.pdf]

Supplementary Material

Gene Networks and Pathways Involved in *Escherichia coli* Response to Multiple Stressors: Supplementary Figures and Tables

Eman K. Abdelwahed [1], Nahla A. Hussein [2], Ahmed Moustafa [3], Nayera A. Moneib [1], Ramy K. Aziz [1,4,5] *

[1] Department of Microbiology and Immunology, Faculty of Pharmacy, Cairo University, Cairo 11562, Egypt
[2] Molecular Biology Department, Biotechnology Research Institute, National Research Centre, Giza 12622, Egypt.
[3] Department of Biology, and Bioinformatics and Integrative Genomics Lab, American University in Cairo, New Cairo 11835, Egypt
[4] The Center for Genome and Microbiome Research, Faculty of Pharmacy, Cairo University, Cairo 11562, Egypt
[5] Microbiology and Immunology Research Program, Children's Cancer Hospital (Egypt 57357), Cairo 11617, Egypt
* Correspondence: ramy.aziz@pharma.cu.edu.eg

Content:

Figure S1: Network of (A) 83 upregulated genes in at least four stressors with medium confidence (cut-off score: 0.4)
Figure S2: The main sub-clusters of the upregulated genes in at least four stressors network
Table S1: Top 250 up- or downregulated genes in each study/sample in heat stress
Table S2: Top 250 up or downregulated genes in each study/sample in oxidative stress
Table S3: Top 250 up or downregulated genes in each study/sample in cold stress
Table S4: Top 250 up or downregulated genes in each study/sample in nitrosative stress
Table S5: Top 250 up or downregulated genes in each study/sample in antibiotic stress
Table S6: Combined list of upregulated in all samples of each stress
Table S7: Combined list of downregulated genes in all samples of each stress

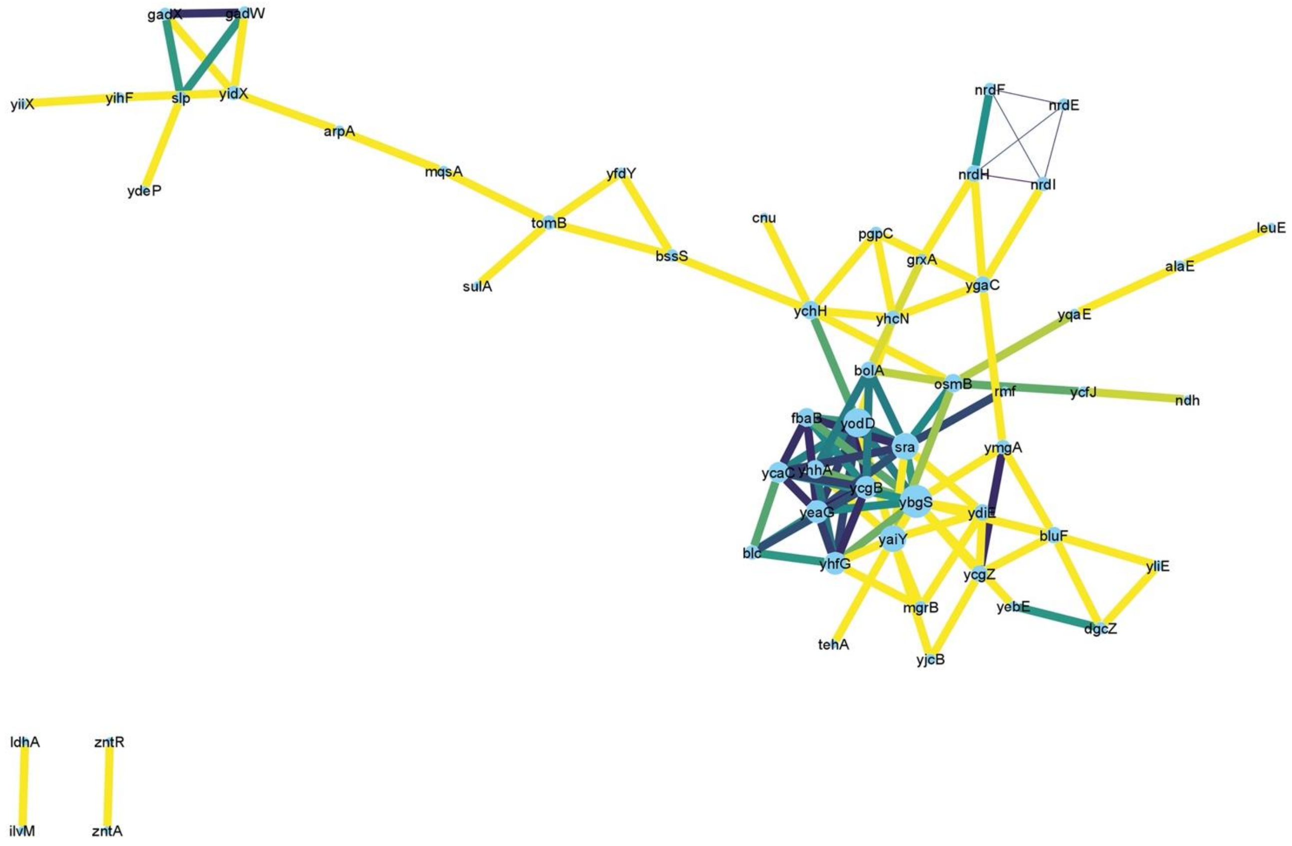

Figure S1: Network of (A) 83 upregulated genes in at least four stressors with medium confidence (cut-off score: 0.4)

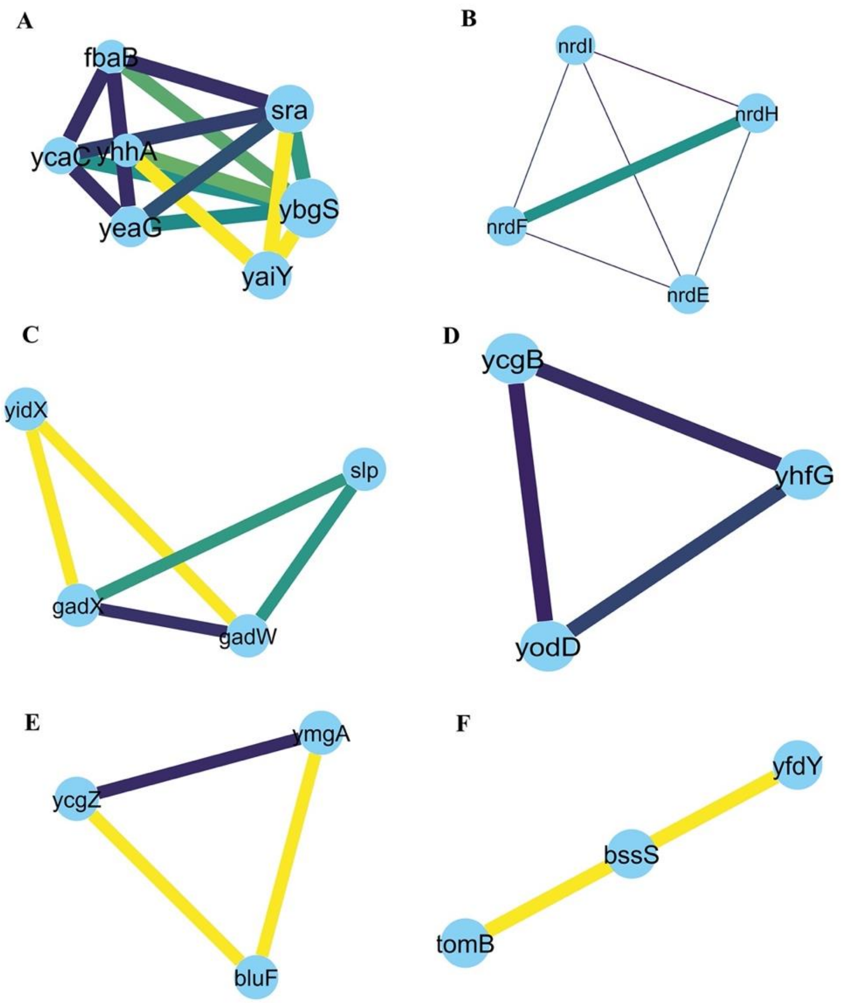

Figure S2: The main sub-clusters of the upregulated genes in at least four stressors network

Table S1: Top 250 up- or downregulated genes in each study/sample in heat stress

GEO study accession	Subtype	Top 250 upregulated	Top 250 downregulated
(1) GSE15534	45◦C	*ibpB, ibpA, alaE, bssS, sdaA, zntR, yhdN, ydfZ, ldhA, cpxP, yhcN, yibA, hslR, fruA, fxsA, clpB, gcvB, grcA, metR, fruK, ybfA, ychH, grpE, ynfK, hspQ, bssR, hslO, ycjX, htpX, yebE, raiA, pspB, ycjF, bhsA, argI, fruB, yncJ, pspA, ybeD, mqsR, nirB, phoB, yiiX, mmuP, mqsA, nirD, mutM, pgaD, mngR, yhbU, ybbN, gntK, dnaJ, dgcZ, yafE, hslV, yrhC, spy, uspB, yhfY, mgrB, yrdA, soxR, phoR, uxuA, ybdH, ompW, narP, htpG, ybdL, metB, pspC, mgtA, ydeP, arfA, rlmE, glgS, rpoD, yibT, pspD, yjfN, prlC, miaA, yddM, ydhQ, frmR, rpoH, uspG, yneM, holC, metF, yqjI, dnaK, yjdJ, ydeS, yjfY, yoaG, bolA, hcr, yffR, marA, ysaB, osmB, clpS, yciS, yhfZ, gntP, yibG, relE, chpS, ydaC, ymgE, yafD, yodC, dmsA, ybeL, metJ, argA, yciM, nrdD, pmrD, yfdT, malX, yneL, cnu, yhjY, uspD, relB, frmA, argF, malI, ldrC, uspF, ydfK, yhhA, ygdI, bdcA, uxuB, yfdY, pepT, argC, hslJ, yehD, rhsJ, yaiY, yibS, ycfJ, yffQ, chpB, yhfX, yjbJ, dacC, arsR, yjjJ, udp, yecH, insL1, clpP, tqsA, ybjX, uxaC, ilvY, groL, lon, insL2, gntT, fucO, artJ, prlF, pphA, yodD, torI, mmuM, metN, dmsC, ycbJ, yhcC, yphH, mdtK, insL3, yohJ, pspG, gltS, fucU, yqfA, otsB, nfuA, gspB, ytfH, frdD, aaeX, dmsB, csiR, ytfK, tomB, yfcZ, frdA, yeaR, ygdR, sbmC, torR, yccJ, argB, afuB, hchA, ldtC, ralR, yfgI, yhaV, wrbA, ydhV, yhfW, emrD, ytfJ, chaB, preT, ydeT, ynaE, yqjE, bsmA, hslU, yhbS, yhfG, clpA, yfiE, psiE, ygaC, garD, mcaS, groS, phoP, ybeZ, yjdI, yoeB, yfdK, rpmE, ghoS, ybbA, yqjA, yjcB, smrA, fucA, yibW, yjbT, ccp, ybjQ, mlc, fdhF, ypjA, degP*	*pyrB, cyoA, cyoB, nuoE, sdhC, pyrI, cysJ, sdhD, cysI, acnB, purE, sodA, cysD, sdhA, ndk, ilvC, purM, cyoC, thiE, sucA, nuoF, ydjN, pyrC, cyoD, aceA, nuoG, livJ, leuC, cysH, thiS, cysN, sucC, carB, codB, thiF, purF, atpG, purK, gltB, leuB, sucB, atpD, aroA, codA, trpE, guaB, cvpA, purB, thiH, ppc, aceE, purL, thrB, thiG, aceF, glyA, purH, hdeA, nuoL, nuoI, atpA, lpd, znuA, nuoH, thrC, gltA, tktA, nuoC, folE, sucD, trpB, nuoM, rnb, hdeB, nuoJ, fbaA, gatA, sdhB, glnA, guaA, trpD, thrA, speD, thiC, uraA, lysC, fhuF, gltD, trpA, gatC, pyrD, cysK, cysA, nuoK, purC, cysC, gcvH, purT, zinT, cysP, xanP, aceB, glgB, serC, gdhA, dppD, leuD, gatB, livG, cyoE, pntB, cysW, pykF, murE, hisD, nmpC, sthA, carA, potD, gpsA, hisC, ghxP, ydiJ, cysM, rnpA, asd, exbB, secA, aldA, nuoN, rplQ, rpoA, murF, purD, gnd, upp, icd, hisH, pntA, cysU, yciW, lysP, trpC, hisA, metH, yhgF, rne, prs, accC, plaP, speE, ybhC, dppA, atpH, deoD, dppB, ppsA, dppC, spoT, livF, hisF, livM, ilvI, purN, gcvP, adeP, rpmF, tsx, efeO, ybiC, ykgM, hdeD, gcvT, yidD, rpsA, atpC, glyV, pal, wbbJ, hisG, rpoB, rpoC, yceA, pdxA, nupC, pheA, gatD, yhbJ, ompF, pgk, slp, yncE, pcnB, borD, maeA, opgH, rplB, metG, ilvB, ilvH, fumA, xerC, pheT, aroH, purA, yhjE, ydcI, serA, rpsK, leuA, wbbI, metE, dppF, kgtP, dapB, hisB, yeiG, opgG, accB, rpsD, aroF, yhbY, mipA, rho, trmH, waaF, rpsS, atpF, aroP, gatZ, nlpA, glyS, rimM, amn, glgX, livK, aroL, fabG, katE, yidC, rplD, mqo, ybgF, gltT, pdxH, yigB, aspS, rpsP, pnp, yagU*
(2) GSE40557	58◦C f=2	*pgaC, pspB, zntR, phoR, clpS, rhsJ, pspA, phoB, entF, mutM, stpA, rhsA, xylG, yciM, mqsR, ycgH, ybeD, yafE, waaS, pgaD, yafU, aceK, yphD, mqsA, htpG, atoB, yibA, yahN, yaiP, yahA, ycjX, fepE, pspG, yiaB, ydaY, pyrF, pspD, corA, yraK, ftsH, ydfR, ydcC, wbbH, kdgT, tusB, ybhM, creB, creA, lon, yncD, yagK, yqiG, torY, ycjF, sfmH, yibG, pspC, yqiK, ydbD, bglB, yliF, tfaD, ykgG, bglG, fimE, ydjF, insH11, yfbN, waaQ, waaP, ymfD, yadM, yqiH, pphB, waaB, yiiE, xapR, hslV, higA, yiaA, ykgF, agaC, emrK, yegJ, bluF, yhaV, yjiT, yciH, ydfJ, wecH, yagM, ymgD, ybfG, yjfL, yfdF, yadK, phoE, csgB, ais, yjeJ, yjbJ, yfcV, nanC, yfdV, yjhI, ycdT, ybeX, yjaA, ygeN, yjhE,*	*hycA, cysJ, adiC, hycB, hyaA, citD, rcsD, mdtE, sufA, gapC, ydgH, hydN, cysP, hypB, prs, ispG, serS, yjiG, hypA, dtpA, mrp, gatZ, serC, pta, lolB, skp, glyA, cysI, ydjN, nlpI, gcvR, sugE, ansA, epd, sufB, gatY, purE, ygdH, yjjV, nuoB, aroG, sufC, ybgF, dicA, rnb, fruB, rstA, cyoA, citE, ymdB, focA, ptsH, yceF, ybgA, folD, grxB, nuoA, maeA, xanP, guaA, pdxY, ispE, gcvT, gatA, oppB, sapA, pnp, ybaB, opgH, yieP, nuoC, proP, xerC, nfi, dauA, glyQ, purK, kdsB, ymfI, artP, citC, rsxC, ispA, hypT, plsX, clsB, ackA, ydeA, hyaB, ydgA, rsmB, lysS, cmoA, yehY, nadK, ppsR, ybhC, nudB, mtlA, purA, lhgO, tpx, ydhS, cysH, hyaF, accB, artQ, yqcC, hycC, yfcG, npr, hypD,*

		frc, rem, rpoD, yjbI, rhsD, yffP, bolA, yidL, wcaD, ygbT, xylH, yeeL, mlc, yojI, ykgH, pppA, yfbK, hscC, yneL, alaE, setC, yhcE, fimD, yffM, waaR, alsA, waaZ, yphF, yqeH, yihF, yehA, ygaC, yghF, yicJ, hflC, ydeQ, yhdZ, ybeY, afuB, yfaQ, ydaQ, ycdU, nfuA, yjfJ, osmB, ydjH, ydeO, essQ, ydeM, ygbF, yhhH, ygeV, ybeZ, prlC, yqeK, frvB, htrE, melB, yghT, yihO, narP, fsaB, yihV, waaU, yhfL, yaiX, ydaC, yjiV, yjfM, yhhZ, ydeR, waaO, creC, yhdY, clpA, caiF, frvR, recN, lacY, bglF, frwB, insH8, tdcG, ilvC, soxR, ogrK, ygeQ, ybaV, ybfD, yabP, yphH, emrY, ihfA, yehH, hflX, yjeH, yihN, yadL, idnK, xylF, fimF, yjgX, yhcA, wbbI, frwD, yghO, yfcQ, yafD, hflK, yhcF, ydfC, glvC, ydeP, rusA, ybbN, csgA, yhjB, yedV, fucP, ttdT, polB, yqiI, mrr, psuG, oxc, fucK, yqeB, ompL, zapB, waaH, insF1, yagH, cbrA, ymgA, yffL, flxA, insB1, yjgL, ytfI, creD	*ybdK, nuoE, gadW, ybhP, yciI, galT, sufD, dhaL, osmF, cydD, msbA, ychJ, yacC, sufE, rodZ, ydcL, tsaB, nuoM, nuoF, dadA, hycF, menD, artI, yccA, ygiB, yhbJ, infA, yebK, udk, rsmA, ybiH, nuoG, cbdB, ycfL, bdm, nupC, tam, ydiK, aspC, pdhR, glnA, yedP, yedE, yhfG, yebV, cysD, csiD, mlrA, ada, rhlE, accC, yobA, cpdA, zitB, folE, rsxE, yhjX, opgG, moaA, artM, gpsA, ybgC, yddM, dam, yejL, bcp, fldA, fruK, galM, prmB, purU, dapD, epmC, yeaK, waaF, yeiP, asnC, serA, yeaH, ribA, yqaA, yfdC, ycfH, nhaB, racR, sufS, lysC, poxB, ymjA, crp, acrZ, ydhF, yddW, yeeA, uvrY, murF, tusC, potB, dhaM, nhoA, tktA, dbpA, hycG, ybhG, alaA, ydiH, menF, purM, fdx, oppC, hybO, typA, potA, purN, ggt, csiE, ydgK, speA, nanR, murE, ybiT, selA, cutA, yheO, pepE, tolC, sapB, map, fabG, cyoB, asd, yecN, hemB, moaB, blc, ycbL, pal, lolC*
	58◦C f=3	*pspB, pgaC, zntR, pspA, mutM, rhsA, phoR, ybeD, yafE, phoB, entF, yahA, wbbH, stpA, rhsJ, mqsR, clpS, ycgH, mqsA, yjeH, pgaD, yciM, waaS, pspG, pspD, yibA, pspC, ydaY, fepE, creA, atoB, pyrF, ydjF, htpG, yahN, yncD, corA, ydbD, tusB, ycjX, ftsH, lon, yfbN, yjbJ, yafU, ymfD, yiaA, yphD, xylG, yaiP, sfmH, yagK, ycjF, bglB, waaP, yfdF, waaB, glpQ, ymgD, ydjH, yciH, aceK, ydeR, waaZ, ydfJ, ydcC, pphB, bolA, creB, yjeJ, emrK, higA, ybhM, ais, ykgG, yqiH, yraK, narP, bluF, ybfA, yliF, yiaB, yfjD, yibG, oxc, yhcE, yqiG, yfbK, ykgH, yfbP, ydaC, wbbI, rem, yqiK, idnK, yqiI, yadM, ydbA, wecH, frwB, bglG, waaQ, kdgT, phoE, yjiT, smrA, wzxB, waaR, mlc, yhaV, ycdU, ydfR, yigF, ycdT, yphF, fimD, rfbC, xapR, ygaC, yneL, ydhZ, ydaQ, rhsD, ykgF, ushA, flxA, frc, glf, yegJ, nanC, yjhE, fimE, torY, yagM, osmB, ygbF, pspE, caiF, iraP, ihfA, htrE, ydeM, ybfG, yjfL, creD, tfaD, yeeL, yedV, recN, relE, wbbL, arpA, yhdW, yafS, alaE, yffM, zapB, hslV, appY, ynfK, ybeX, creC, yhfL, gudD, insG, ycaK, cohE, ompT, yjhI, agaC, yadD, yfdV, yhjB, prlC, matP, caiE, yffP, relB, hscC, ydjE, wzzB, tfaP, intK, yqeH, csgB, yabP, soxR, ybaV, yadL, yehB, ybeY, yqeK, yhdZ, yliE, waaO, clpA, mgtA, cadB, rhsH, ygeN, yadK, ygbT, yjiV, yafD, ybeZ, ybbN, pppA, ydeQ, ftsA, yhiJ, yiiE, yjfJ, yphH, afuB, cusB, yjfM, ygeF, yihN, yffL, yehA, yfaZ, alsA, yfeS, rpoH, ytfI, grxA, yghT, ogrK, rpiB, ygeV, yfcV, yaiX, ybjH, potE, flgL, mrr, rbsC, yidL, yjhC, ycgI, hflC, yhhH, rof, ydjG, frwD, yjaA, yccE, yffN, sgcQ, ykiA*	*adiC, cysJ, hycA, hypB, hycB, mdtE, hydN, citD, xanP, hyaA, cysP, ygdH, yjiG, sugE, ymdB, ybgA, gcvR, hypA, yjjV, gapC, gcvT, xerC, nuoB, prs, ackA, cyoA, gatZ, ydgH, rcsD, rstA, nfi, purE, rsmB, acpT, csiD, ansA, citC, artP, yieP, yacC, ispA, serA, nuoA, mlrA, folD, dtpA, lolB, fruB, yehY, pdhR, yqgE, guaA, cysI, sapA, citE, yqiC, gatA, lhgO, cmoA, epd, ybgF, pdxY, tam, aroG, gatY, menD, clsB, asnC, purK, rnb, nanR, sufA, dauA, glyQ, ybhP, nadK, mrp, pta, yhjX, murF, ybdK, mtlA, udk, ispE, yfcG, ydjN, yheO, pnp, avtA, oppB, dicA, pdxA, rsmA, yjiX, tsaB, tusD, cydD, ispG, hypT, rnpA, pcnB, nlpI, cyoB, phr, lysC, rhlE, ychJ, hypD, yigB, psd, ytfL, yobA, lpoA, yfdC, maeA, lysS, glyA, yciI, yebK, nuoC, ybhC, focA, phnP, rodZ, fetA, ybaO, dapF, npr, yqcC, ppsR, proP, nhoA, yiiQ, bcp, nupC, glnA, zitB, rsxE, waaF, artQ, nuoE, yebS, fhlA, ampD, yhfG, trmH, alaA, accB, nagC, hyaF, ydeA, opgH, rsxC, dbpA, sufB, purN, ygiB, ydhS, sufC, yejL, gmk, purA, folE, ybdF, pabA, moaA, menF, ybaB, spoT, csiE, hypE, serC, galT, potB, serS, gadW, rssB, rstB, uvrC, hycC, plsY, purU, kdsB, ycbL, osmF, nuoF, cutA, hycE, pepE, yejB, yhbW, rsxG, ydcN, menH, alkB, accC, yhhN, nuoM, allD, yeiR, dnaX, ybiH, secM, fdx, cra, grxB, yeiP, bcsF, ybgC, apaH, potA, dnaB, yafJ, prmB, ptsP, ydhF, kup, ibaG, purM, bcsG, dam, gpsA, hybO, yddW, citF, yghU, gph, fadI, yddM, hycF, wecA, yidC, yehW, skp, tatD, tusC, yjjG, msbA, rsuA, racR, sufS, blc, ravA, pspF, holB, ada, glnQ, alaC, nemA, yceF*
	60◦C	*pspB, pgaC, pspA, zntR, rhsA, phoR, ycgH, mqsR, waaS, phoB, rhsJ, pspG, yahA, clpS, entF, mutM, pspC, ydaY, stpA, pspD, wbbH, pgaD, yibA, ybeD, mqsA, corA, fepE, yibG, waaZ, ycjF, waaR, waaP, yafE,*	*hycA, adiC, hypB, cysJ, hydN, hycB, hypA, cysP, citD, xanP, lolB, sugE, gapC, ygdH, ybgA, gcvR, ispA, citC, yjiG, dtpA, aroG, rcsD, ymdB, acpT, cyoA, yqiC, yieP, csiD, asnC, fruB, rstA, pdhR, rsmB,*

		ydjF, yciM, waaB, pyrF, rfbC, yfdF, ycjX, wbbI, xylG, ydeR, creA, yadM, ymfD, yafU, tusB, appY, yiaA, yjaA, yiaB, yqiH, bglB, yphD, htpG, yqiI, wbbL, glf, ftsH, ybhM, yncD, ydcC, yahN, yagK, alaE, wzxB, lon, yfbN, waaQ, yraK, htrE, bglG, aceK, yqiG, sfmH, ydbD, yedV, emrK, yphF, waaO, yhhH, rhsD, yaiP, bolA, phoE, atoB, ydjH, ymgD, higA, kdgT, creB, ydbA, yjiT, ycdU, ydjE, yjeH, yciH, dkgB, yhaV, pspE, pphB, yhfL, intK, ydfJ, idnK, ykgH, yjgL, yfbK, waaU, yegJ, yjeJ, xapR, nanC, yjfL, yabP, yffN, ygeN, fimD, bluF, ftsA, yeeL, csgB, matP, yffM, ynfK, yneL, yjbJ, insH11, ydfR, ydaC, yjiV, rfbA, narP, yfbP, yadK, relE, ycdT, creD, ykgG, yliF, ybcW, glpQ, tfaD, yjhE, yagM, rfbB, caiF, rem, iraP, yqiK, rpoH, yehB, pppA, ydhZ, smrA, ais, yjgX, ompT, ykgF, yfdV, yadL, yffP, relB, yhdZ, wecH, rpiB, fimE, hslV, flxA, yiiE, yliE, creC, ybfG, mrr, yccE, mlc, yfcV, ydbL, yfcP, ygaC, casE, agaC, setC, ybeY, torY, yghF, wzzB, ypdI, yncJ, cadB, yhcA, yfaZ, clpA, ymgA, yjfJ, ygeQ, ynaK, alsA, ytfI, ushA, ybeX, cohE, yqeH, ycaK, ogrK, ydeQ, yjfM, ydeM, hflC, yehA, wcaD, yhjB, yhiJ, ftsZ, osmB, ydjJ, ihfA, oxc, ychE, prlC, melB, yihF, xylF, ybbN, emrY, yafS, fimF, ghoS, ygbT, yidL, ybeZ, rhsH, soxR, hscC, nfuA, yjgN, waaH, ygbF, gspB, yehH, ybjH, yeaJ, yhcE, ydfC, yjhI, csgE, ompL, yffO, psuG	*yacC, gatZ, pdxA, xerC, folE, cutA, mdtE, serA, purE, mlrA, hypT, dicA, zitB, yqgE, nfi, gatY, ackA, nanR, prs, yfcG, ybgF, artP, rsmA, ispG, ansA, bcsF, nagC, tusD, npr, hyaA, ybhP, yheO, phr, rodZ, ydgH, yiiQ, lhgO, yjiX, ydeA, ispE, menF, yfdC, nuoA, ybdK, yqcC, yjjV, ydcN, dauA, yhjX, yejL, clsB, sdhC, apaH, nuoB, rhlE, menD, yciI, serS, pnp, purK, psd, ytfL, rnpA, plsY, pcnB, avtA, recO, ydjN, udk, bamD, trmH, nadK, sufA, alaA, tsaB, ybaO, rstB, murF, mnaT, cydD, fetA, ycbL, mrp, rsxC, tam, rnb, dbpA, yobA, mtlA, spoT, ybhC, fhlA, alkB, hypD, rssB, yejB, guaA, yehY, folD, potB, rsxG, pepE, tusC, ygaH, citE, focA, epmB, nupC, pbpG, sapA, lysS, yfbR, ybaB, phnP, epd, folM, blc, fruK, artQ, yebS, fdx, waaF, kch, nlpI, nhoA, lpxP, yghU, potA, ychJ, lpoA, recX, rspR, yejF, dnaB, pspF, infA, gatA, yhfG, rssA, iscX, rsxD, mprA, wecA, dctR, tatD, bcp, serC, lysC, uvrC, cmoA, yidB, yeaK, menH, purU, pepB, yfcL, yeiP, arnT, queG, sdhD, glnQ, ppsR, glyQ, minC, era, kup, gluQ, yciC, rnc, kdsB, yeiR, pabA, yccA, yedK, moaA, ybaP, gmk, fadI, gcvT, yhbW, pdxY, csiE, fnr, yigL, ampD, ymfI, yghB, yjiY, ptsP, yhhN, yigB, accB, ebgR, ymjA, rluE, epmC, yceB, ydcP, yebV, sufB, allD, gatD, ycfH, yfgG, osmF, ptsH, yqaA, galT, opgH, purN, aroA, ybgC, maeA, glyA, ydgK, dapF, yjjG*
	71◦C	*pspB, pspA, pspG, yjaA, alaE, ycgH, rhsJ, pspD, yahA, mqsR, mqsA, pgaC, pspC, entF, rhsA, ydaY, corA, waaS, phoR, zntR, yafE, stpA, clpS, phoB, higA, yphD, ydjF, pgaD, yhfL, pppA, mutM, waaP, waaZ, ydbA, pyrF, tusB, ycjX, yiaA, yagK, ybhM, bolA, kdgT, wbbH, xapR, ytfP, cadB, fepE, rhsD, ycdU, ybeD, yciM, htpG, phoE, waaB, yibA, yfdY, yafU, glpQ, ydbD, ddlB, yjhE, ftsA, yadM, ynfK, gltP, yqeH, yfbP, ilvG, yqiI, rpoH, ykgG, lon, yahN, appY, mrr, waaU, yihO, yfaZ, pphB, sfmH, ftsQ, waaR, zapB, yliF, ycdT, ccmA, bglG, smrA, agaC, pspE, creD, yihV, ycjF, wbbI, xylG, ydfJ, yhjB, fucK, narI, ogrK, sugE, yncD, ftsH, potE, dusC, ygiZ, tamB, matP, yhhH, ydcC, yhdZ, rcnB, ompF, yjiV, yiaB, psiE, ilvM, yabP, ymgD, tfaP, dkgB, ykgF, yjfL, ftnB, waaQ, htrE, yicH, iraD, rfbA, yigF, wecH, wzxB, ilvC, melB, fimD, ybaV, ispH, yjbJ, yphF, mgtA, yfaQ, ycfZ, yciH, ykgH, yidX, xapB, aceK, ydhZ, yehH, nlpC, yffN, atoB, btuB, ycgJ, setA, waaO, idnK, frvR, ykiA, yeaJ, ynaK, rpoS, wbbL, ydfX, caiF, ydbL, glf, caiE, rfbB, yffM, intK, iraP, yhaV, creA, cobU, ybbN, emrK, hcaD, murC, secA, yaiP, lacY, yqeB, yjcF, trkG, insG, yjhG, yghF, ydeM, borD, rem, yifK, yihN, atpC, yegJ, glgC, ydeR, araC, crl, csgB, hflX, grxA, cbrC, rfbC, yadK, mrcA, yacL, yfhL, ynbE, aqpZ, yffL, ybfA, narP, yjeJ, hscC, yoaF, yjiT, ompT, cusB, cohE,*	*hypA, xanP, sugE, rstA, gapC, pdhR, ybgA, hypB, hycA, rhlE, cysP, adiC, lolB, ispA, yfdC, rssB, hydN, gcvR, nanR, asnC, ackA, yhjX, folE, mlrA, sdhC, ymdB, rsmB, aroG, yqgE, lpxP, nagC, ydcN, citC, ybaO, cysJ, yejL, dtpA, cutA, apaH, rcsD, yciI, yfcG, yjjG, hycB, acpT, hypT, fetA, fruB, dbpA, ybdK, ybhP, yacC, tusD, gatZ, yheO, queG, cyoA, bcsF, yfgG, yccA, rsmA, ygdH, gatY, rssA, nadK, yjiX, yqiC, rimL, dctR, zitB, rnpA, fnr, fdx, tatD, yiiQ, ycfH, ebgR, yobA, ansA, yfjL, dauA, tam, viaA, recO, sthA, citD, rspR, pbpG, pcnB, dicA, yfcL, ydgK, pgpC, ydgH, nfi, alaA, yafQ, udk, gmk, phr, ymjA, artP, nuoB, yieP, rnc, infA, yfjU, folM, pdxA, prs, rluE, tusC, yigL, iclR, csiD, rodZ, menF, guaA, nlpI, nudJ, tadA, ygaH, nudK, potB, zur, npr, sdhD, lit, rseB, nuoA, epmB, focA, plsY, psd, rsgA, alkB, ispG, yciC, ychJ, gluQ, allD, fadI, rsxE, xerC, yejB, yqgF, glnQ, ravA, ycbL, mtlR, ybgF, ispE, tsaC, gatD, bamD, spoT, xseB, ytfL, kup, folD, potA, lhgO, tatB, bcsE, tsaB, gntR, rbsD, msbA, prfC, yebS, sapA, yjaG, epmC, yihI, blc, purK, ycbK, yciT, ydiH, intZ, fetB, yjiN, pepE, ygjP, adeP, purE, yedK, era, iscX, yijO, rhtC, yhfG, yibF, ydeA, dosC, yrdD, nhoA, yagN, rstB, dnaT, clsC, melR, recX, nupC, fruK, bcsC, yqcC, avtA, nadC, serA, queF, lysS, yjiM, rsxG, nemR, pabA, lipB, ydcP, yjaH, sufA, yejF, ybjX, arnC, ydjN, sodA, argO,*

		ybjH, yagH, intB, yqhC, torZ, cobS, ymgE, yojI, yqiG, yadL, cdaR, ydcA, rhaT, yihF, slyX, yciU, ftsI, wzxE, rlmB, tfaD, ushA, yifE, yneL, yraK, setC, ftsW, rpiB, ccp, sixA, rpsO	*yidA, yghX, rnlB, fecI, clsB, yedP, yehY, lolC, ybgC, trmH, ydhL, yebW, arnT, pka, mnaT, uvrC, dnaB, ydhS, fldA, gatR, mepA, ybaP, hsdM, yjiG*
(3) GSE42675	50◦C 4hr	*ibpB, ibpA, bssS, alaE, sdaA, yhdN, zntR, ldhA, ydfZ, cpxP, hslR, fruA, yhcN, fxsA, clpB, fruK, metR, grcA, raiA, ybfA, hslO, pspB, ychH, ynfK, ycjX, ycjF, yebE, pspA, htpX, fruB, bssR, grpE, argI, hspQ, yncJ, phoB, yiiX, gcvB, spy, mmuP, mutM, mqsR, uspB, ybeD, mngR, yrdA, bhsA, ybbN, hslV, narP, clpS, yibA, dnaJ, yafE, mgrB, gntK, glgS, ybdH, dgcZ, bolA, uxuA, nirD, ydhQ, mgtA, yibT, mqsA, yneM, pspC, nirB, pgaD, arfA, htpG, ybdL, phoR, ompW, yqjI, uspG, soxR, rlmE, rpoH, holC, rpoD, ygdI, yciS, metB, marA, ymgE, yfdY, osmB, yhfY, uspD, ybeL, chpS, yciM, cnu, yddM, miaA, pmrD, frmR, prlF, metF, flxA, uspF, argA, yjdJ, dnaK, tomB, chpB, dacC, yafD, relB, metJ, frmA, relE, mmuM, fucU, recF, yhbS, yffR, bsmA, yjdI, prlC, torR, yqjA, yccX, torI, argF, pepT, clpP, yjfY, ybjX, yefM, gltS, nfuA, yfcZ, udp, hokD, yiiS, pspD, yecH, yjfN, yodC, lon, nrdD, yodD, hslJ, ytfH, malI, ilvY, clpX, yhaV, metN, ypfM, yaiY, argC, ybjQ, creA, araC, uxuB, ygdR, gntP, frdD, otsB, yqfA, sbmC, yhjY, yhfZ, artJ, ycfJ, gntT, csiR, clpA, degP, ldtC, ybgS, ryjA, mlc, yqjC, ydeP, uxaC, yjbJ, rdgB, tqsA, yffO, hicA, hcr, yhhA, malX, yjcB, pfkA, mdtK, ytfK, soxS, yjjJ, dinJ, ycbJ, yhbT, groS, ilvM, ydeI, chaB, yphH, yoeB, ygbF, dmsA, smrA, ftnA, yqjE, yjdP, slyX, zupT, frdA, groL, ygaC, phoP, pspG, yffS, yqhC, bdcR, mcaS, ygiW, bdcA, argB, higA, argH, blr, arsR, yhaH, pgpC, hchA, sugE, lrhA, csgA, yciZ, matP, ogrK, ybeZ, wrbA, nlpE, yoeG, yhfG, nsrR, ftsH, yhcO, yfdT, yqjK, acuI, dmsB, bfr, norR*	*pyrB, cyoA, cyoB, nuoE, acnB, sdhC, cysI, cysJ, purM, sdhD, sdhA, sodA, cyoC, pyrI, purE, sucA, cysD, nuoF, thiE, pyrC, nuoG, ydjN, ndk, codB, aroA, ilvC, cysH, purF, cyoD, nuoC, carB, ppc, tktA, thiF, aceA, thiS, purB, leuC, purH, gltB, guaB, purL, livJ, trpE, purK, cvpA, codA, nuoL, atpD, thiG, aceE, nuoH, nuoI, thiH, atpG, aceF, sucC, cysN, gltA, hdeB, thrB, glnA, pyrD, nuoM, rnb, leuB, fhuF, glyA, trpD, fbaA, thrA, sucB, cysC, glgB, trpA, uraA, nuoJ, lpd, sucD, hdeA, guaA, trpB, gdhA, folE, lysC, nuoK, znuA, atpA, thiC, sdhB, thrC, rne, cysW, sthA, gcvH, metH, dppD, xanP, purT, cysA, murE, hisD, speD, aceB, cysK, hisC, gatA, exbB, ghxP, gatC, purC, purD, livG, spoT, pykF, ydiJ, aldA, secA, cyoE, pheA, gatB, gltD, nuoN, plaP, yhgF, gpsA, murF, serC, cysP, leuD, pdxA, yciW, adeP, cysM, dppC, wbbJ, potD, cysU, yhbJ, gcvP, ybhC, yeiG, maeA, deoD, dppB, accC, slp, efeO, nupC, hisH, purN, trpC, ppsA, hisA, carA, exbD, nmpC, lysP, yncE, metG, opgH, borD, dppF, asd, ydcI, rpoA, pntB, tsx, hisF, speE, yceA, suhB, yidD, gnd, trmH, icd, waaF, ybiC, aroH, hdeD, amn, prs, gcvT, pntA, wbbI, katE, mqo, ilvI, ompF, rho, glgX, xerC, atpH, mraY, rnpA, dppA, ansA, glf, livF, rpmF, gsiA, fhuA, rbbA, rpoC, potB, pheT, metE, rpsD, yeeN, pta, rpsK, livM, serA, rpsA, yqgB, rpoB, glsA, upp, xseA, yigB, pcnB, rplQ, ilvH, gsiB, atpC, malE, mreC, fumA, gcd, opgG, secM, hsdR, plsX, pal, hisG, rplC, ygdH, fusA, ptsP, rplB, yhbY, leuA, bfd, nlpA, rimM, gatD, tyrB, ilvB, ygiQ, atpF*
(4) GSE11041	46◦C till 10^8cfu/ml	*ycaC, hchA, rmf, alaE, aceB, wrbA, yiaG, tktB, yccJ, yeaG, uspF, ycgZ, treB, fbaB, ymgA, gadX, aceK, clpB, osmY, yeaJ, yegP, intF, ybiC, gfcE, gadB, hdhA, ghrA, malX, osmB, mtfA, cbpM, yliE, ydbC, ygaM, aceA, copA, ynaI, rutB, marA, ymgC, eamB, cspD, ivy, add, nrdI, ykgJ, puuA, chaB, ybcH, sufA, idnR, ymgD, yijE, rcnB, mscS, nanM, ibpB, otsA, rof, yecH, glgA, bhsA, ybaY, degP, srlA, nrdE, rclB, dsdA, rpiB, htpG, pgrR, yphB, rpoS, potE, sad, mdh, iraD, gadW, nrdF, dsdC, bolA, elaB, mtlD, ydfD, ygaU, sra, raiA, ydaU, ybeL, higA, cho, yahA, marB, thiC, elaA, yhaV, yjhR, yfcG, relE, allR, csgF, gltA, yfeA, talA, dadX, xylR, nudL, yiiS, mqsA, yiiD, rpoH, yebW, lacY, dnaK, yhhA, glgP, ybbN, ygiW, yqjH, umuD, yhbO, treF, blc, ridA, yjiJ, sieB, amyA, csiE, aegA, nrdH, acnA, uspD, rbsD, ybbC, ycgM, yhaC, ibpA, yfcE, wecH, dcp, yqhC, glgB, yehX, tdcC, ykgF, maeB, mdaB, fimC, hicB, cysQ, gadA, pnuC, dgt,*	*flgB, ynjI, intZ, cspA, ymfA, mgtA, rhlE, fadL, yaeI, flgJ, gtrS, plaP, potC, cysH, speB, ypdA, fecA, sbp, yafW, lolC, cspE, rnk, nlpA, pdxY, fixC, yfcU, speD, deaD, emrB, fadD, oppF, rlmG, gtrB, yfcL, cusA, yddL, pfo, ycbZ, flgH, ynfG, yfdX, ffh, mdlA, dppF, dusB, sthA, potA, frmB, lolD, fdoI, thiQ, yaiI, tsgA, cysJ, yecC, btuB, ychJ, cysI, yfiM, yhbE, ybiU, yehP, codA, yiiX, fkpA, secD, yncD, yccT, alaA, yifK, bluR, dtpA, proY, ycjP, oppD, dicA, fau, uidA, rsxG, gspF, ruvC, ebgC, cyoA, wzxB, sanA, nohQ, yhdW, oppC, rffH, ydjN, waaC, murJ, bcsQ, oppB, cmtA, yjeH, fabD, fliL, nudB, uvrY, yigF, wecD, fecC, aer, rluA, yfdR, mreC, ybgP, rlmA, hyfR, speE, yeaY, rffG, galR, ftsX, fdoG, fdoH, ybiR, rimP, yeeE, yegD, livM, pgpB, pitA, rtcA, rnpA, phnN, eutJ, rhsO, wbbJ, fdnG, yidA, cbeA, yejL, gntR, yraJ, yafT, thrC, wecC, citE, mntP, rnd, slyD, menF, sppA, zapA, basS, ybfP, serS, xseA, fdhE, flgM, opgC, smpB, potD, nhaB, ygjH,*

		fucU, yieP, fimA, bepA, melB, yehQ, ydeP, yheO, srlD, yidQ, lysC, yicH, ydiQ, ybgJ, nlpD, pgpC, ppsR, galM, ychH, yhaI, msyB, fucR, yqaE, symE, qorA, ftnB, yedR, yhhQ, appY, can, cnu, uspB, fimG, creB, waaA, fxsA, amiC, cbpA, cfa, ycgB, ysaA, glgX, napB, fecI, deoB, katG, yqjE, creA, aldA, yfjU, mnmE, bioF, narP, pepT, rimJ, tag, mglC, ybfA, nfrB, lldD, mobA, plsB, yehW, eutR, nudF, treC, fabB, tehA, coaD, yfbU, grxB, yagN, uhpA, ycbK, ybdF, yqjK, hemF, higB, yhiI, yjjJ, yafS, cadA, ydgD, yhjX, ytfP, dacC, sxy, mrcA, lgoR, cysE, mdtK, metR, ybjP, yeaD, ypjD, yidB, ariR	*yeiE, trmJ, rsxC, gmk, rfbA, nfi, hyfG, secF, glf, baeS, ylaB, truB, mzrA, ubiI, racR, rsmG, yedF, hemE, murI, ygbA, yqcC, gspD, fabH, rhlB, codB, hpt, ackA, ansB, yrdB, thiP, insA8, astB, nrfD, putP, mscM, rspA, yjfN, uup, lysP, ydgA, prmC, torT, yidK, wbbL, ydiB, pheP, yejM, fliY, ypjA, nohD, ubiJ, nadC, gsk, tsaB, ygdQ, yhdE, mepA, mcrA, pheT, waaQ, ftsE, yccU, bamD, plsX, yjgA, rlmJ, ybcJ, ygjP, folD, infB, yjfP, rfbC, yqgF, cysP, hybA, phoQ, hyfA, pgaC, insA2, metC, ygbM, yebC, wecG, lpxB, ribB, nusA, ubiX, gspJ, prfA, mgrB, zitB, alsB, yjdC*
(5) GSE20305	50◦C 10min	*ibpB, ibpA, alaE, nrdH, bssS, ariR, ydeR, ymgC, pgaD, gltS, ybcW, ydeT, ydeS, mqsA, uxuA, dadA, mtn, metR, ybfA, csgB, yiiS, metB, yffO, ydiP, yfdT, hchA, hslR, marA, ybdH, yqhC, uspD, yhcN, hslO, eco, phoB, hokD, raiA, ycgZ, mmuP, yoaG, quuD, rclR, bhsA, uspG, grpE, nrdI, ybgS, nrdD, yfdS, kbl, metL, yfhH, pspA, uxuB, yaiY, dadX, yjjJ, yneO, eamB, clpB, pspC, pspB, yhjC, yfdK, uidC, metN, ymgA, intF, yffP, trxC, yhhA, gltP, ydeJ, ydeQ, potE, yibT, leuE, ryhB, yadM, yjfY, eutQ, slyX, ylcG, cpxP, spy, yiiX, yeaR, tfaP, ldhA, araC, chpS, ygiV, yhhQ, hlyE, yffR, metJ, yneL, bolA, yadL, ybjM, htpG, sdaA, psiE, ycjF, yncJ, appY, ypdI, kilR, ypeC, yjfN, mdtK, tfaE, ygiW, ychH, valV, emrD, yffN, sgbE, yggP, yeeW, dnaJ, higB, ycjX, yfeN, eutT, yfcO, ogrK, valW, ydaE, yjfM, rpoH, yhfY, ilvY, eutD, htpX, dnaK, yadK, feoA, yhfW, csgD, metA, yphD, kdgR, prlF, yhfX, ymgD, yaiO, bssR, bfd, narJ, glgS, gnsA, yfaH, sugE, higA, yhaV, ycfJ, yphE, mokC, hokC, hdhA, ecpR, narX, hslJ, sibB, yfdX, rusA, pptA, mngR, yohK, betI, tfaS, gadX, mntH, yfdL, uspB, zupT, norV, mmuM, yqjF, relE, grcA, corA, ybcN, iraD, ysaB, xylB, yjhF, csgA, djlC, wrbA, zntR, allB, apbE, yeaM, yggF, pfkB, araB, casE, appA, sufA, ymgG, yffM, dmsC, chaB, ycgI, xapR, pspD, ybdL, yfbV, rhaT, osmB, fdnI, yffQ, ybeL, ivy, cdd, yjaB, elfA, pgaC, yhjD, gldA, pepT, gfcC, yeaJ, ytfK, ydhY, ygaC, dacC, yhdN, torY, ykgE, ygbA, rem, yfdY, fxsA, kdpC, ypfG, cspD, ulaG, torR, aes, ynbB, relB, norR, yffB, aphA, yaiS, araF, yhjY*	*cysJ, flgE, gtrS, flgF, flgD, tap, flgH, carA, trpE, flgG, flgC, codB, gltB, oppC, livM, cheR, trpD, fliK, flgB, folK, gltD, leuD, livG, aroA, pyrI, dppD, gatZ, oppB, thiF, waaF, fliG, nuoI, oppD, pyrB, gcvP, dppB, fliH, nuoE, cheB, leuC, yciW, cysI, nuoH, yfjW, malE, flgJ, cysH, fliM, livF, cyoB, nuoF, sucA, pnp, dppC, cysN, thiE, thrB, cheY, serC, nuoL, thiH, murF, cysD, nuoJ, hsdR, thiS, argC, fliR, ilvC, glnA, nuoG, livH, tktA, argD, fliP, nuoM, thiG, cyoC, thrC, yhbJ, flgI, waaL, waaC, thrA, ridA, artJ, fliE, oppF, gatD, opgH, suhB, nmpC, dtpD, ansA, fliL, ygiQ, ymdA, gdhA, nuoK, pyrD, gatC, metE, livJ, carB, oppA, recG, glnL, guaA, dppA, cysC, bcsF, leuB, ptsG, gsiB, evgS, tsaA, asd, hisM, dppF, flgA, cheZ, potF, pykF, spoT, fliN, aceE, rho, flhB, ompF, fliO, potG, cysU, trpL, ygdH, hisC, prs, fliI, metH, acnB, aceF, aldA, ycaO, opgG, sdhA, yeiB, gltF, purB, dosC, purD, malP, pflB, yibQ, bluF, rsxD, glgB, murE, yidD, sdhB, pfo, hisD, adhE, mreC, epmC, cheW, sucB, ampG, trmH, ilvH, ymfA, nuoN, flhE, pyrG, hisQ, yfcA, gsiA, codA, adeP, cyoD, leuA, uraA, dosP, rnb, hisA, secA, cysP, flhA, ydjN, gsiD, hisH, trpB, atpA, yecR, ynjI, cysT, fbaA, hrpA, yeeR, ilvN, folE, envZ, pdxA, solA, eptC, pta, purL, gcd, gabT, rnpA, yjgL, flu, yceI, cyoE, truB, folP, mtlA, pyrC, potB, tyrT, mreB, ppx, tyrU, tyrV, argA, agp, cysM, rbbA, cysK, tyrB, pyrL, yjaH, hisG, malK, potH, plsX, cspC, lpxH, ribB, sbp, yeaD, trpA, argG, bcsG, gsiC, sdaB, sthA, cheA, atpF, wecB, ynjE, argH*

Table S2: Top 250 up or downregulated genes in each study/sample in oxidative stress

GEO study accession	Subtype	Top 250 upregulated	Top 250 downregulated
(1) GSE20305	H_2O_2 10 min	*oxyS, yaaU, yaiY, kdpB, yhfX, araB, atoA, ygcS, glgS, rclR, insM, nupX, mdtO, prpR, eaeH, rusA, arsB, ygfS, arnE, nikC, frlB, atoD, frlC, fepD, nirD, ryhB, phnK, gntP, ygjV, yjjW, yhjX, torY, yjiJ, leuO, ygaY, pitB, torA, prpE, tnaB, yjeO, hyaC, yiaK, ygjJ, hyfC, nanT, mhpA, rhaB, paaE, garD, yjhE, yehP, yhgA, araD, yhfT, eutM, prpC, yjfC, yacH, prpB, yqaE, rclC, yjgZ, idnT, yegT, oweS, narK, yicR, agaD, nirB, yfcQ, hycB, ygaC, cmtA, yccT, ygcQ, ycjO, dtpC, hyaB, yjdN, ybiA, yhbQ, rspA, yfdR, yegK, bglH, ygjI, yccM, recX, yjfN, citD, ppdD, ghxQ, sfmH, yfaQ, aes, ydjL, yhfS, ynfA, rdlD, prfH, nrdG, phnL, php, arnF, yeaV, rutG, caiT, yohJ, yqcE, araA, eutQ, ssuC, ygfK, xylA, yieL, fecD, yhhN, dcuD, yidK, arnT, ybeQ, gspL, prpD, xdhB, hsrA, wcaK, xapA, emrD, yifN, lsrC, uidC, yegL, bglF, aslA, tfaS, yaiP, ycaI, eutD, yfjX, eamB, tdcA, xylH, treR, yfaH, preT, yddH, hofQ, idnO, ydeT, ydhK, phnG, yjeM, hyfA, dgoA, ydhT, hyfB, argK, hycD, rclB, glxR, dmsC, aldB, gspC, fucP, yqjI, yfcC, idnD, nanA, gspO, mdtE, yigF, yhfW, yihP, blc, casE, yjbF, yeaX, ygfT, yibI, scpA, ldrD, sgcC, yfgF, yicN, yghD, ompN, ulaF, dinD, mdtB, gudP, yadS, dcuC, yfdQ, citG, cynX, yfeR, ycbU, fdnI, yfcJ, fepB, caiE, fhuE, scpC, yjfI, ygbJ, yijE, yehQ, elfA, tdcB, agaS, yegS, yiaM, agaW, yfcU, allB, nrfG, ydjM, ybgQ, ygjQ, rybB, yphH, qseB, dadA, treF, yphD, lacY, rhmR, hyaF, gspF, yfjQ, focB, ygaY, hdeD, rihB, nrfC, clcB, nohD, yfcU, kdgT, mdtC, zntA, eptA, slp, yeeW, yeaR, tnaA, yeeT, ybcI, ygcU, hyfR,phnH, mdtP*	*gtrS, gpsA, ymfA, flgJ, waaL, oppB, upp, intF, purN, mepS, glyA, purB, dusB, rplD, plsY, yagB, flgG, gtrB, pyrI, atpF, oppC, purH, yidD, rpsK, purC, yfjW, dksA, suhB, purM, rplW, fis, pnp, fliT, guaB, rnb, prs, ydaT, pyrC, epmC, holA, atpH, rpsD, rpsH, rplB, rplX, lit, carA, yqgB, yqeI, ybhC, hflC, gdhA, cheA, fadL, pyrB, aceE, glf, rplF, ycaO, accB, evgS, nuoF, plaP, livG, atpE, rpsS, yfjH, puuB, artQ, caiF, cheY, fliM, rplE, oppA, tgt, thrU, oppD, cspC, plsX, fliP, rpoA, rplN, rph, rfbC, wzxB, purD, folK, dppB, tap, leuU, rpsG, cvpA, gcvP, potB, fliK, sucA, envZ, waaC, guaA, csgE, damX, yeaP, livM, thiF, mcrA, parC, serC, pyrG, nuoE, fliS, nuoI, argQ, speA, cheW, rcsA, ribB, argD, evgA, ygiC, rne, fdx, appY, mlaB, yebY, flgH, argV, ppc, yccS, yeeN, purL, gcvH, rpsC, ubiI, hflD, secE, amyA, dosC, rpsN, yeeR, atpB, cheR, nuoJ, eptC, xseA, wbbI, bamC, argS, argZ, rsxB, yagU, yhbE, livF, ydiJ, purF, wecA, murA, argY, tolA, fkpA, prfC, nuoC, mppA, tyrT, mutY, pntA, atpA, fusA, hemY, emtA, rplP, gcvT, holE, tsaB, gtrA, truD, sapF, tatC, ybjC, dacA, yhbJ, dppD, tyrV, spoT, fabG, nuoH, rplV, uup, glnA, yagM, yjgL, ispF, ispD, wzzB, trpL, gph, potG, pheS, carB, ybgF, vsr, thrB, thiS, rpmG, rpsQ, codB, pal, ybjS, secY, ansA, menH, hisF, accC, yedL, trpB, atpG, mprA, prc, tyrS, tolB, hisS, trpC, mreB, proV, livH, rfbA, rplR, cyoB, ptsI, fabD, icd, iscX, thiE, pcnB, topA, thrT, nfsA, nusA, purT, pflB, hflK, rplA, rhlB, hemX, lpxD, opgH, malP, serT, lptD, rnpA, fold*
	H_2O_2 90 min	*ymfM, beeE, umuD, ymfR, umuC, jayE, yebG, recN, stpA, recA, insM, tfaP, dinD, fdnH, phnE, recX, glvC, ghoT, sgbH, nikC, phnC, stfP, yebF, hyfC, yiaY, ghxQ, sgcB, fecD, dgoR, mcaS, yiaN, yhfY, yafN, mdtM, paaE, fdnI, pppA, mdtO, yjdN, osmB, tnaB, phnK, intK, fucK, yhgA, paaB, sgbU, paaA, alsA, eaeH, glvG, yjjW, hycC, eptA, ybiA, agaD, yiaW, ygaY, slp, cirA, agaS, yjcS, mhpA, yghO, yafO, ydcV, yhfX, hyaC, adeD, ulaD, agaV, frvB, rhaM, mhpT, prpE, uhpT, creD, hycF, paaX, cusA, iraD, ycjO, kbaY, ygfS, yghT, yjgZ, ymiA, xanQ, eutM, ygaC, phnI, yidP, hycB,*	*yccJ, talA, yahK, amyA, csgE, clsB, csgF, yeaH, flgL, ydaM, otsA, ycgB, malP, otsB, dosC, yedL, aceE, dld, gdhA, gatB, astD, dksA, metF, nac, manX, yodD, gatA, gcd, yagU, gatZ, pfo*

		malG, waaH, yjeO, mdfA, yqjI, ygjV, rlmG, ecpC, yhhN, yidX, gntP, yhjX, adiC, lexA, atoD, pyrE, nohQ, ymgG, satP, mdtI, yibI, yahF, rdlD, nupX, yjfF, ybdH, yidB, kdpF, ycjP, hycD, clcB, zntA, yhbQ, ylcG, phnG, tpr, phnF, tnaA, yddH, ddpB, treR, yhcN, aaeX, garD, frdC, ydhI, hokE, ynfM, yaaJ, frwC, yicG, prpC, wcaK, entE, yhbP, bglB, ccp, ecpA, yfdQ, araJ, ydiK, uxaB, ybdZ, gpr, entS, lgoD, intQ, ymgD, citG, ybaV, yjhF, mhpB, ybhS, gltS, fecA, yegK, arnF, yicJ, ssuC, lacY, xylE, xdhC, argX, cmtA, yfcJ, yfdY, nepI, sgcC, betT, rhmT, yliF, ygaY, yjiK, cynX, fes, dicF, fimH, lgoT, mhpE, rttR, ydhK, uhpC, cusB, paaG, essQ, tynA, prfH, eutP, mhpR, garP, atoA, mscM, prpD, ybgP, rutG, yliE, tfaS, hyfJ, yebE, phnH, yicI, uvrA, focB, qseB, mutM, idnD, tdcA, nohD, recT, viaA, ydeR, yhaM, dinF, gudP, rspA, yfcU, dinB, scpC, ygaQ, lldP, xapA, rlmF, idnO, hydN, ykfJ, ydbL, ychN, queG, ryhB, arnT, ydeT, ygjJ, sfmD, hypF, arnE, yifE, fieF,trmA	
(2) GSE61736	7% H_2O_2 till OD_{600}=0.5	*nuoJ, gfcC, ybiR, flgC, ycaC, ybaT, ynbD, chbC, zapE, ykfB, fsaA, nanE, araC, emrK, yfjM, argD, phr, setB, lplA, xapB, yciY, yfdY, yffO, sulA, xseA, rnlA, insA6, yieL, yqfA, ybiB, talB, gspJ, hofM, leuB, rlmL, yjfF, eutA, sgcC, fadH, fimG, ycaD, ylbF, argF, iscA, wcaJ, elfG, nusA, hisQ, yigI, hycC, modB, yidE, yaaJ, yjhU, yjaB, thiH, yneE, phoP, iceT, potF, flgI, entE, ygjJ, dmsC, gshA, rplA, dgoD, amn, atpD, purM, tusB, ulaC, pdxH, gpp, yfbS, frwA, yidZ, zntR, fecD, yahI, yoeD, yaiO, alx, ycbX, yafJ, rhmT, metQ, yhfX, lldP, corA, kefG, yibQ, rseC, mdtO, ypjA, zwf, gspH, ydeP, ygjQ, rtcB, alkA, sxy, ushA, bcr, intA, yjgR, ypeA, speD, ccmD, ycfQ, tpiA, metE, fpr, ytjB, cysH, ccmF, frwB, potI, bglA, tag, nrfE, yedF, ytfL, nadC, pdxB, proX, glgA, paaJ, uhpC, mlaE, gspK, yjfY, rmf, sbp, rseA, dtpA, cpxA, yhhT, glmS, yegX, yafF, rzpQ, rrrD, lpoB, rssA, fldB, fadR, bisC, yqiK, rng, yqeB, murJ, proW, ybjJ, ccmE, yeeS, dauA, glcA, caiT, gspD, glnK, tsaA, aaeA, alaC, rhtC, argH, yfgO, tam, yhiM, acrE, tldD, yadE, ycbU, oppB, rsmB, higA, cbdX, yicH, ymdB, cirA, lgoD, yedZ, ybgK, rep, mlaA, gmhB, mhpT, arnT, cadA, xylA, chiA, birA, fadB, ghxP, pepD, ydiB, uxaC, cysG, elfC, narP, tatD, ldcC, panD, pqiB, malF, yfiF, hycG, ghxQ, frwD, eda, ssuA, yhfL, obgE, hcaE, sgcQ, satP, pgaC, yadD, cysB, hybB, acnB, ygeA, hflC, ycbJ, dtpB, srmB, ftsA, gspF, yicJ, aaeB, metB, ygcP, ytfT, dusA, yecM, iscR, ymfD, asnA, lptB, gspB, rhaS, arsB, accA, rcsC, yqiH, caiB, mutH, ycgV, yfgF, aaaE*	*ygcW, fbaB, frmB, sppA, ruvC, cspA, gtrS, ybhK, prs, rutE, folD, purB, mokC, ydeN, gadA, rcsA, damX, yciO, nemR, cusC, pcm, exbB, frr, acrR, yjfJ, yjdM, ycjG, menH, yecN, kup, rpsP, priC, hslO, gadX, agaR, fepD, rutD, rplJ, trmH, fadD, mprA, gsiB, gatD, yagE, ygcR, rpsT, yiaD, lptE, opgD, sfmA, fbaA, miaB, lsrD, yhcB, xylE, yibN, znuA, waaZ, xapA, lpp, ribC, gsiC, bioD, kdsB, evgA, gcvA, pykF, yfbV, glnG, hinT, nth, ftsY, sbcD, rpsH, holA, glyQ, rpsN, xdhA, cspE, ybgF, cysZ, dcuS, ydaM, topB, puuB, rplF, ymfE, fryA, glcG, gadB, cysS, trxB, gyrA, manA, yffN, casA, murR, rsmI, ycjR, yghA, cdh, waaR, yibA, cysJ, mhpF, ppiA, ligA, proV, ydcO, prfC, ydeJ, insP, hflX, recF, yjiA, tig, hypD, alaE, malY, phoE, yeiW, treR, ydgA, oweS, fdnH, yeaP, phnP, ulaE, trpD, nuoB, sucB, ydhB, fliC, rarA, ycjF, yegD, fepE, caiC, nagC, tolQ, ybfA, ydaU, sufA, entC, yfbT, napA, ydcP, pmbA, pyrG, zipA, cusS, eutK, fabI, pepA, ybiW, nuoC, ysaA, mglA, ptsI, ynfF, narQ, lepA, rnpA, ygeO, symE, trpE, fliA, yjjW, mpaA, yoaA, ybaY, citC, rihC, eaeH, phnH, glnS, yeiE, hemA, adhE, ugd, srlQ, hrpA, yqjI, yeiP, mscM, ybeZ, mutL, mfd, plsB, yciE, ileS, hscA, garK, citD, yegU, ybiJ, fixB, gltD, pfo, ybhD, frdD, yagI, mdtP, yjiR, dcd, ydhK, yafQ, thrA, cyoB, ynjH, prc, intD, ybgD, uxuR, ldhA, ompF, pepN, xseB, astB, moeA, yajQ, fiu, yqhD, gapA, luxS, yliE, ribA, epd, dgoK, hybO, rhmR, rhtA, ccmH, fucO, wcaG, yggN, fruB, paaF, rsxC, napG, flhB, opgH, pheS, galE, ybhH, napH, mhpD, moaA, pstB, coda*

(3) GSE56133	10 uM H2O2 60 min	*yeeE, cysP, yeeD, cysH, cysJ, cysD, ydjN, efeU, cysI, yojI, yciA, yceA, efeU, nrdH, cysA, entC, yddA, yedN, cysU, yciW, cysW, nrdI, opgC, cnu, emrE, ycgZ, lpxT, yliE, ydiY, murJ, yhdU, cysC, pyrD, cysN, ydiE, trmN, adeP, rluC, nrdF, yedN, sixA, holD, elyC, gnsB, cirA, rsxA, leuE, ydgI, gpt, ybjE, fiu, yihG, plaP, fes, ybfE, stpA, opgE, pabA, ycgX, yigI, rph, mgtA, folA, gtrA, ydgK, ampG, dusC, ybjG, yedN, queD, yafK, tolQ, mdfA, rsxB, ubiX, apt, rlmG, rlmA, cysM, pyrF, mdtJ, infA, iraM, tsaB, purE, ldtB, tyrP, pth, ybgC, xseA, holE, glsB, queA, fepC, nrdE, entS, ygbE, exbB, lipB, pheP, fhuD, dusB, fepB, entE, suhB, yggI, epmA, gluQ, rimI, rnpA, cdh, bluF, aroH, hcaR, ibaG, yliF, yghG, ygiM, ttcA, ybjO, ygdQ, panF, rfaH, tdk, yjjA, fecC, mreD, thiB, cusR, mioC, udk, proY, rnd, trmA, fecD, ybiV, ytjC, trmI, mltA, yedJ, rlmH, fepD, sufE, ydhJ, gsk, mutY, fliR, fis, mltD, yhdT, fliE, entH, potA, purK, entD, rnhB, rpsU, tsaC, rsmF, mepH, ygiQ, maa, emrD, rsmC, fhuE, ymjA, fepG, nudE, rep, rimO, entB, ftsB, entA, yhbY, rarD, mepS, lysP, tsaA, recQ, atpI, cysK, yqeG, secG, mnmA, yidB, rnt, yegQ, mltF, tusE, dbpA, rsmG, mdtI, cmoA, recF, yagI, rnc, ndk, lgt, purR, lnt, mreC, yebT, aroK, arsR, yeaY, essD, greA, yhjV, bcr, fhuB, wzzB, thiM, ybaK, nemR, yjjG, ycdZ, rlmC, glpG, yhjJ, rseC, fecR, entF, cusS, glpE, yicG, yncD, ydgC, fepA, rsfS, yhbE, corA, srkA, yahA, ratB, acpT, rlmB, arsB, amiB, yedV, yigB, emtA, rhtA, ytfL, efeO, aroM, menA, ariR, fecI, yeiR, apbE,fadD, acpH, dnaC*	*tdcA, ycbJ, gudP, garP, ansB, ydhY, garD, nrfA, ghoS, ysaA, napD, bssR, yjjI, narK, napF, gldA, yniA, abrB, dcuC, tdcB, yqeC, hybO, yahN, nikB, narG, ompW, hypB, ybaE, yehD, mocA, lgoR, yjiM, wrbA, dtpB, napA, srlA, frwA, ynfE, yjjP, melR, nirD, cdaR, garL, ravA, yqgA, ttdR, ccp, ynfK, atoS, yecH, ygjR, pepT, narI, yhbU, narJ, yfbS, narH, ydbC, ycgB, yjfN, uspE, preT, yehC, gpr, yqfA, sgcX, frdA, ydiL, ybeL, hypC, pka, yccM, yaiV, yccJ, uspC, yidE, yaaJ, adiY, hcp, nrfB, dmsB, tdcR, ydjY, zraS, yadI, napG, yidF, melA, srlR, ychH, ycaC, ftnB, sbmC, alaE, uxaB, ygdH, yfbM, glgS, dhaK, manX, araC, yhbV, gadE, aldB, yfaH, gpmM, yhbS, blc, dcuB, uspD, hybA, dctR, ybiH, ybiA, lgoD, ydcH, ygiZ, fruB, mmuM, dinD, rayT, ybgA, mlc, malK, hcr, uxaC, ydhV, feoA, malI, csiE, frdC, rhmR, dmlR, ygeK, yzgL, fsaA, glk, ygeV, yhbT, frdB, yhhY, nikA, cdd, napB, uspG, napH, chpB, qorA, lsrA, mrr, gudX, mtfA, malS, putA, ybjD, chpS, yejG, ydeN, yeaG, yieP, dgoK, yiiS, yhjX, cydA, hybB, yfeC, smg, feoB, yifN, napC, ampD, ykgE, yidL, ygcB, ldtE, uspA, torT, manY, fucP, arnF, acuI, yacL, yeeY, frdD, fadM, araB, yfcZ, yrhA, pfkA, nagB, hsrA, fpr, yjjQ, yqeB, ytfA, yhhX, yhaO, yeiE, yhhA, ilvC, aegA, cydB, yafV, yajO, idnK, ttdA, murQ, yahB, araF, yfeO, yagU, rof, fucA, frwC, lysU, uspB, melB, ycfP, poxB, rcdA, yfdY, yihO, yodD, yaiE, yaeH, hybC, prkB, yicH, ybdF, exuT, ymgE, bfr, fucO, msrA, yjcE, yhaK, pfo, yafZ, sra, yagJ, ybhQ, ilvE, sokC, ypdI, udp, gatD, rmuC, polB, malF, kefB*
(4) GSE58176	Tellurite	*yagG, ykfB, ydjG, lptB, appA, yffB, basR, cmoA, stpA, ycjD, mutH, acs, rclB, entS, cheR, rhmD, ygeH, ytfJ, alaE, frdB, manA, aas, chiP, cspF, adiY, pyrF, nrdE, uxaA, yphD, ygjQ, nudI, thiB, pfo, ynfN, ydiR, tehA, tatD, csgG, yhaB, sra, osmB, ydfO, yejH, ydiV, purU, yceM, flgB, panC, ybhB, asnC, emrK, cusS, ygeX, ycgZ, hyaB, yjgM, ymgA, ryfA, phoE, ampE, mokB, flhD, ydaT, cspG, arpB, pflD, yehH, yjiR, trpA, asnB, mtfA, ydcF, iscX, ymfL, yeiH, aldA, yeaJ, cyoD, proV, maeB, ycgL, cynR, kdpE, yfbR, yceA, uspG, cysA, yhhZ, gadW, gabP, ykgA, phnJ, araH, fepB, gspG, rstA, ppdA, nirB, gspK, argA, ydhT, yedK, gltI, nagA, hcr, wcaN, hokD, ydjX, ydfI, yfaH, ygaP, yebQ, ynjE, lomR, rlmA, evgS, ydaY, yaeF, mhpT, borD, ycjY, mntP, ynfE, yfcU, ygeQ, yghS, xdhD, yjiH, fbaB, yghO, yadE, aroC, cysD, waaZ, arnE, putP, sslE, yehT, rhsE, yhhA, opgG, mhpE, yohF, sgrR, ykfA, ascF, caiA, yjjP, yahJ, rhsC, ygbJ, yjjZ, grxA, ybjS, yeaP, gntK, yehY, phoA, yibA, glnB, racC, eutR, ycjF, gatD, paaZ, tdcE, yfjJ, paaX, yqeK, pflC,*	*tap, bcsQ, metR, ydiT, yegL, gldA, pspB, nanM, yeeL, pgaA, ylbG, pspA, yhfX, ycbJ, ydbD, metN, wza, casE, yhjR, yjfF, ygcP, ybhH, ybeQ, symE, ygeX, ylaC, scpC, yhhW, yncI, ynfM, ycfJ, narG, cdd, ydcH, yahF, ibpB, yhaJ, narH, yiaV, gadE, ubiX, ybgS, pspE, ydjL, yciX, kefF, potE, yiaW, garL, bglB, lexA, nhaA, lplT, hycG, ftnB, yjjB, intB, fdnG, yfjV, oxyR, ydcJ, eutP, emrY, mcrA, yfdV, tdcG, kilR, fdnH, wrbA, patD, dinJ, purT, alsE, ycaC, ydfC, yegU, tnaC, ydiL, yagA, cirA, amiC, yheO, manZ, rutR, yedI, yebF, yecS, atoD, ycdT, yaiY, hldE, leuE, ydiQ, nanS, ratA, glyA, rnlB, yneL, miaA, ybiA, rcsD, ribA, bhsA, ilvY, yqeC, marA, betI, ftsQ, ypfN, yjhB, ynfD, marB, ppdC, tusE, lhgO, puuP, chaB, ndh, ygcS, yaaW , ybiW, metI, allC, cheR, hydN, rlmE, glyS, gspH, hslU, cho, ibpA, insG, sgcC, ldtC, yaiW, fabR, rcsA, yfjD, hisL, groL, apbE, cbpA, ninE, atoC, yegP, edd, ybiV, ypdF, ydeM, ytfQ, wecD, yfbR, ggt, dmsC, yghB, paoA, kbaZ, hypE, yfcG, yjaZ, rimL, emtA, flgN, yhjE, yadK, mdtM, relE, nrdI, rlmG, rutG, yqgF, nrfB, sufC, yggC,*

		phnF, ydcC, ynbE, ppk, suhB, flgG, zinT, ygeF, cspC, yhbP, nikR, wzxC, wecH, yddG, wzyE, ompN, yajI, insP, yciA, yifL, xerD, yfdS, phnE, yfbP, paaH, yoaB, gltA, ldtA, yadC, ytfA, ndk, yjfP, gshB, ydeO, ydcY, rutD, pepN, ydbA, pyrC, yeeJ, yjhD, ytfH, dsbD, rsmF, gmhB, ybhL, ptsN, rtn, bcr, yhbX, yghG, yfaE, pinR, ycfH, yiiD, ychF, dapD, yhiJ, rlmN, yhdZ, araF, yegJ, pflB, mlc, moaB, napC, gluQ, gspE, msrC, yfcA, yhdX, fimG, yqgA, nudE, yagN, mlaE, yggX, ycaO, hyuA, yobD,	*rnr, yigE, yciM, nusB, ygaU, oxc, yijO, yedY, dgoR, yheT, yfiP, yibG, yjgX, abgT, garD, ydiM, narW, napD, yghE, ydcV, ccmC, prpB, mntR, ftsY, yjhE, yoaE, ybdL, pldB, mgtA, rcsC, ychQ, yhaV, gltJ, yifB, mtlR, lpxC, srlM, lsrD, xdhB, nadR, dosC, rhsH, yieL, tdcC, paaF, ftsA, dicB, yceJ, ycgH, rclC, yccT, cysH, ansB, yfjY, grpE, idnT, purR, ugpQ, artP, exuR, fumC, rpiA, setA, ybaL, solA, yihM, yfhM, ydeA, napH, citG, dnaK, yeiQ, sdiA, yobF, yfdT, phoB*
(5) GSE 19370	peroxynitrite	*glmM, yidZ, pqqL, yiiR, ydaC, paaF, yehB, rutD, yegX, ygeF, yfbT, gpmM, ydeT, rhsB, tdcD, mdtJ, hofN, yiaA, metA, torR, yeeS, coaA, deaD, gudD, ygaQ, tdcG, opgE, yejM, ydiS, yeaR, ybbW, pinR, yhhQ, yhgA, wzxC, serA, fryB, yqeK, pfo, fliD, sdiA, fhuE, ybiT, fdnG, ulaF, ybfG, tdcB, dgoK, yqcA, wcaA, ycfQ, hyi, feaR, ynfK, ydjI, yeeR, ygeW, ydfO, ebgC, gspG, napD, gatR, sapB, creD, yrdB, melR, pgm, alkB, narJ, gspJ, cmtA, ypdE, cusF, flxA, yrhB, csgB, frlB, menB, tnaB, emtA, gspK, yfbN, nirD, ascG, setB, hypE, glxR, torT, yeaP, ydiO, ndh, yhiS, fes, ldhA, pdxY, yhiL, ydiT, yhfU, dmsD, gntU, xseA, maeA, mqo, ydiU, dosC, yhaI, cusA, dgcZ, ygeK, gspL, yfcP, frlD, yidI, msrA, emrK, yfbL, ycgY, cadB, yfiB, rutC, yghX, nudI, ybgO, ddpF, pyrF, gspI, yfjD, fetA, tus, acrD, yiaJ, yajI, hyfI, torY, hydN, intG, yfcI, yddK, yeeP, ralR, yiiX, ygjI, rpsF, wcaH, ybgP, ycgH, ydfA, yqiH, yqeA, fumA, bcsZ, wcaE, yqjI, scpB, rhlE, kbaZ, dctR, ydjF, insZ, yidC, yjgN, sppA, yqjK, yedN, uhpB, cusC, clsC, yeaO, ytfI, emrD, hybA, yigG, wcaK, argP, ycfJ, cheA, yegR, yaeR, aslB, ldtB, bisC, yjbL, yrhA, flhB, alsC, ypjD, xylE, kilR, arpA, rcnA, ydiQ, mqsA, wcaF, yjeM, ilvN, hrpA, yqeH, cysS, xylF, ybjI, yiaV, pntB, fruB, rhsC, yehY, nth, tcdA, rnk, napH, dhaM, yjgL, argS, cbeA, dicB, yeaW, yhhZ, ada, yehM, yeeJ, ascF, nuoN, lyxK, yddW, arpB, yggM, narV, ygiV, yqcE, cheY, mtgA, puuB, yedS, gspC, rlmG, yfcC, yfeO, yqfE, abgR, ybiW, ydjK, yihF, nudL, hiuH, arsB, yjbB, yddL, fabF, ygdG, recR, gspB*	*paoA, yafL, yadM, kdpA, yifB, yceK, alkA, caiA, gltK, yraQ, hycG, deaD, ydhW, ykfA, yiiG, asmA, amtB, betT, yegE, opgC, yjeT, mdtH, quuQ, ydaW, rhaM, ydcN, ppdB, dauA, yjiP, pheP, atoE, fsaA, psuT, yafS, narU, yghR, ybiR, nemR, yjjB, yaiS, pqiA, dgt, yehT, phnG, panC, yjjJ, yhcC, nohQ, yfjP, yjiN, yagE, yfbM, hcr, yiaB, yfeZ, betI, afuB, ycjR, ybjO, clcB, cueO, mhpC, stfP, cynX, agaD, lgoR, bdcA, yafY, mngA, ygaY, rsmE, lpoA, ttdT, ybjM, eutK, cspF, mhpT, yadC, pgaC, galP, flgK, nadE, pyrE, oppF, garK, yhbQ, spy, yibB, cedA, ygfI, ydfZ, yahL, mhpR, flhA, fadL, ybjH, yedE, fhuC, yiaT, priA, yjiS, ydaV, yceQ, ligB, hycH, aaeR, yahB, rluF, ygiZ, ypjF, yaiX, dusA, ybeM, mhpF, rclB, yjgR, alsR, artJ, yraN, ftsN, rcdA, yebO, lafU, yaiP, ylbF, cusS, eaeH, yfgO, yphD, ybbC, ykgL, ddpD, yahN, acrZ, allA, ykgH, prpR, yegU, eutR, intD, ybaK, amiB, ygcW, yidP, recD, rlmB, yagU, yphG, nudJ, ravA, ymfR, hyaD, mdlA, rbsB, ygfM, hrpB, melA, cspB, folK, mqsR, rhtC, yieH, ynaK, yebS, polB, glcA, hicB, fliZ, ybaV, ssuC, yacL, pheL, yahD, yagK, yijO, yjcO, lpxB, thiL, modF, yncG, betA, fucP, tauD, rtcR, fliT, ptsG, ptsG, yneO, aes, ampG, ybiA, glpX, astE, rimO, rlmN, ycgR, yedS, pptA, yejB, intZ, ligT, ldcC, yagF, yjgH, sfsA, yjcC, quuD, wcaM, ecpC, phoE, folP, yqeF, prpD, fecC, ulaD, yagL, moeB, mdfA, yhdP, tesB, phnK, ycdY, gltD, yagP, macA, paaZ, modC, hsdM, yneE, ynjA, yidH, lpxK, ybeQ, gspA, yafW, thiP, potF, dmsB, dsrB, yidA, leuO, ygbI, adiY, yagJ, fliP, waaS, grcA, gntT, elfA, yjgA*
	H2O2	*glmM, ymgG, agaB, ybiW, torR, rhsB, ybfG, ynjI, gpmM, ygeF, pqqL, fdnG, ytfI, phnK, dmsA, speC, yhcH, ydfD, yhiL, yhhQ, ygaQ, pgm, yhfX, yiaD, frlD, yehR, yqcE, rutC, ribA, ygjV, dosC, paaH, yrhA, ypdE, yihF, ydfO, fabF, yqeK, arsB, ybbW, pinR, yiaA, emrK, yajL, yiiR, ydiO, ydaC, elaD, yfiP, cmtA, cheY, rhlE, lpp, ebgC, ycjY, hycI, gspC, torY, hiuH, yegJ, cbeA, ygeN, proV, arpA, fliD, cheB, fdhD, yfbL, cutC, pyrF, yqhH, rlmG, yidI, yhiS, cusA, frlC, yhbP, wcaK, ycgX, hemA, tdcD, yiaJ, pyrD, cusF, yhfY,*	*caiA, fryC, torC, rsxE, ydaV, yegU, allA, ybcJ, nadE, yceK, ygiZ, gudX, yibB, yeeT, ykgE, fumB, yehT, puuP, tfaD, yfgO, yfjR, yacC, mdfA, idnT, pheL, ybjE, lacA, leuO, hsrA, uvrA, eutK, thiL, yjgH, agaD, fepC, frmB, emrY, dinJ, rihC, ymfR, prpR, yhbQ, yffM, ycaI, fimH, polB, rsmC, leuE, kdpB, yfcJ, yidJ, cobC, cspF, gntT, yahD, yafX, yfbR, ymfI, ftnA, yqeI, cueO, rlmC, gsiB, yphC, fliS, ybdJ, acpS, allB, ispH, ykgL, nrdD, rcdA, wzzE, ydcN, ybaV, yneG, shiA, lacY, mokC, clcB, ppdB, ylbF, hcaF, proA, yafY, yneE, murI,*

		mdtO, sgbE, priC, torT, dsdC, yfbP, emtA, yciK, hyaA, ulaF, ydiN, dapE, ydeT, yqeA, narV, cadB, selA, wecE, hofN, nudI, ydiK, pfo, yedS, frlB, ygfK, yedN, yiaY, ycaD, metA, xseA, ygeO, yqcA, feaR, yggF, yjiC, ynjH, yhfU, ygeW, wcaH, nikA, zraR, apbE, yqiJ, ettA, rhtA, ycgL, yddK, yhdW, ybjI, flgL, scpB, sad, yfdT, elaA, racC, ydfA, apt, paaF, dgcZ, alkB, cdh, zinT, ralR, csgC, glvG, flgH, hybD, cbdA, dinG, yrdB, ydjY, wcaG, gatR, sgcC, chiP, ydjF, dicB, serB, kbaZ, yigF, thiB, recJ, yhcG, paoB, hypE, srlA, emrD, frc, yjfK, setB, ilvC, yfdL, yncI, paaD, nuoK, yehB, fetA, yiaN, flgK, purC, fimF, hcp, ygjI, gph, viaA, kdpE, yiiX, ybhK, ydiH, rhsC, yecT, yjfL, tmpR, yifN, yjfZ, mdtE, acpT, rplI, bluR, serA, mdtC, ydjE, yqiH, rcnA, atpB, creD, yiaW, yqiI, ygjH, cspD, yffN, citB, fldA, yghF, ydiL, yhfZ, yidK, yqeJ, ddpA, cusR, ycjV, torZ, flgN, sdiA, ygeG, yjhF, ycgH, frvB, gatY, yicG, nfrB, yedW, hybB, wcaF, serS, ydeP, ybgP, norR, yddL, sfmF, yiaV, glxK, nuoN, flxA, yraI	*wcaL, ymfJ, lysC, yedA, yehP, ycdU, rayT, fadK, metE, thiP, ybdN, yabP, fhuC, citC, nirC, ydaT, pphA, lafU, idnD, yaaX, paaG, dauA, waaQ, ypjK, ycjR, ybeQ, yahL, ybbY, flk, mdtI, ttdT, kptA, casB, elyC, yeiB, yfjQ, hsdS, zapE, ybcO, ykfG, yafZ, ycgR, ygfI, eutS, yebO, opgD, betT, norV, endA, garL, eaeH, rzpQ, mutT, rhsH, yebZ, ygbJ, ykfC, yadM, ydaW, ygbM, zur, narG, yfiL, yebQ, oppF, pqiA, ydcD, mmuM, moeB, yjiN, tyrP, lysR, paaE, ymfA, fimD, psiE, yiiS, clcA, ybhM, borD, panC, chbG, pagP, bdcA, ydfU, mutS, pcm, gstB, tatC, citX, metB, dapB, alaA, ycdT, emrE, yoeA, treB, yfaQ, yobD, rsmJ, yafL, nemR, dsbE, cmtB, rhsE, yfeX, dsbD, rnhA, yjiV, ycjS, mnmG, yigL, fliF, qseC, ampE, ytfE, aphA, ydeE, yegE, rlmM, fixC, sapC, yrbG, ligT, dinF, waaS, adeQ, ybcI, ulaG, yfaA, rhaM, menA, ppx, ylbG, modE, yfjJ, wcaJ, holB, metF, rcnR, ycbF, atoE, ygdB, yaaA, nadC, ydhK, hsdM, rhmA, recQ, frsA, prpC, ydbD, abgA, phoE, yehH, hypD, murG, chiQ, frmA, pstB, ybiO, hycA, gcvA*

Table S3: Top 250 up or downregulated genes in each study/sample in cold stress

GEO study accession	Subtype	Top 250 upregulated	Top 250 downregulated
(1) GSE 11041	15◦C till 1X10[7] CFU/mL	*cspI, ybfG, ydfR, safA, ydeJ, yfbP, yeaG, ycaC, cspB, ydfK, yiaG, ynaE, phoH, sra, gadE, dctR, yfjP, amyA, ycgZ, ybaY, ymgA, osmY, ygaM, ymgC, puuE, ycgB, ydbC, glsA, patA, fbaB, yeaH, nepI, pinQ, adhP, yeaQ, astE, trpC, ybgA, yzgL, ydiZ, gadX, cspG, tktB, lpxP, blc, yceI, ydaM, elaB, rmf, phr, pnuC, gadB, yehX, dgcZ, pinR, bluR, trpA, ydcJ, katE, bluF, yniA, mqo, yehW, betT, cspF, fiu, yedA, ybiX, poxB, acnA, gadW, yhfG, yceJ, ybbC, ggt, astA, yibF, clsC, nadA, narZ, yfcG, otsA, ygaY, metF, yidQ, gabD, yfjW, aroG, uspF, gltA, yodC, arnT, yaiT, ybgS, gmr, glgA, ytfK, gadC, ymdB, dosC, dld, gdhA, elaA, rpoS, fic, kptA, yjcB, rsd, bcsE, ydcI, potF, chaB, rcsA, ynaJ, tas, iraP, astC, lhgO, sdhC, csgF, yohD, tehA, hcaF, gabT, ansB, yjiJ, yehY, glgP, ydhP, osmF, yqfA, pgpB, curA, puuA, proP, rimL, ytfT, dbpA, slp, mdh, sufE, yghU, mdtJ, yedI, yecA, trpB, ydeE, feaB, sufA, shiA, ybiC, nlpD, nrfA, yccJ, gltD, proV, sucD, yhfX, yqjC, ychQ, puuP, yeeO, glxK, yegS, mpaA, glgB, yieE, yjbJ, rssA, bolA, yahO, sugE, yebS, nrdF, msyB, argT, tehB, dppB, serA, melB, trmN, ybdJ, yaeH, yegP, dps, yqjF, yejG, yddM, yjbM, gltB, wrbA, ybdK, gadA, yieP, ydiH, ydcZ, yqjE, emrY, rtn, yjbD, ydfV, talA, otsB, yodD, alaC, sdhA, gcd, malI, malZ, ghrA, arnA, ygdI, yqjD, yceK, pgpC, bcsF, ycbK, yjgH, cof, lsrG, yfgO, sdhD, yrbL, yoaC, insZ, pgsA, yhcO, zapC, ycjG, crl, csrD, yehE, xylE, wecH, slyA, ydjZ, yhbO, nrdE, bsmA, sthA, btuE, yfcF, mlaF, potG, marC, ychH, ydiV, pepT, allE, treF*	*nmpC, stpA, yghG, borD, yhjX, ompT, fimI, carB, carA, yjiY, yliE, sdaB, yliF, sdaC, gntT, uraA, fimA, yghF, yiaD, fecA, fimC, tsx, malE, alaA, pyrB, codB, lrhA, plaP, pqqL, codA, pyrI, cydA, feoB, fecE, sgbH, mgtA, grcA, asnA, mutS, sieB, purB, glnA, fecC, treB, fdnH, feoC, yifE, ansP, purL, glnQ, mdaB, tdcE, pliG, upp, hycE, ccmA, sanA, katG, hmp, fdnI, fbp, nohD, treC, emrB, yfcZ, araC, cysH, cmtA, yhgF, guaB, rluA, proY, pfo, glnP, napC, purD, napB, purH, hybC, nupX, yfcS, glnH, trxB, fecR, recG, thrA, cysD, cysN, pck, marA, eutK, hsdR, ratA, yaaA, pppA, nrdA, dut, yqhC, focA, yfjR, nohQ, thrB, htpG, ybjO, intS, rlmI, fimF, ptsG, emrA, yqgB, tdk, yacG, ulaC, rsmD, yjbG, purT, mog, mobB, yedF, fdnG, ccmB, thrC, rimO, srlB, murI, bacA, gatC, pstS, eutM, hycH, cydB, slmA, kduD, napG, eutC, glnL, yggM, lipB, hpt, zapD, hycG, trmH, pta, yihT, hybE, dfp, yfaE, ynfG, phoU, marB, tdcC, nikE, phnN, rfaH, yjhQ, napA, intR, fliN, ackA, trmJ, hda, ydfE, yhhL, galE, recN, bioD, purM, yjiX, proB, ccmC, groL, moaE, narH, fetB, tsgA, cysJ, ydjJ, cydD, frlC, tdcD, narP, rep, dicC, ypjA, lysP, ampC, rapA, galT, gpr, argS, ykgA, yihL, groS, hybF, ygbM, yjgZ, ravA, ddlA, hypB, ompR, ccmH, afuB, fadH, menA, ygaC, yigF, ycfZ, ebgR, aspA, btuB, fabI, ftnA, asr, agaA, rdgC, udp, flhE, rsmF, mltA, ulaB, gsk, dcuR, ydiB, ygaH, hchA, glcD, cynT, hslU, argB, znuC, cdd, yajQ, alsB, sbcD, metK, nagE, yrfF, hypE, yaiC, yhjB, prmA, flgH, ybhM, rhaT, fecI, trmA, nth, yfeH, basS, ridA*
(2) GSE 61736	15◦C 4hr	*rpsC, nhaR, rplA, rplV, trmD, mazG, thiC, glmS, rpoB, hemG, pgi, rlmM, panM, waaO, aceE, ppiC, eptB, glpK, tdcD, ascB, yidL, rpsO, melR, glpD, leuS, rnr, gspD, zntR, ygcB, ftsW, gpp, pepQ, dinD, alaE, fusA, arpA, yeaD, ebgC, cadC, acnB, purH, kptA, rpoE, infB, sfmD, garD, trkA, fxsA, glgP, katG, hofM, yidK, yifK, malS, rplF, pnp, nlpA, ubiB, polA, glnD, yhfK, hemD, yijO, htpG, osmY, bamA, panF, yhiJ, srkA, xylB,*	*yjfC, yfcD, murD, dhaR, holB, tynA, aaeR, yejK, yeiE, uvrC, efeO, ugd, iraD, yedE, yegQ, lsrR, mutS, clsC, abgR, fliM, phnF, wcaL, feaR, rcsB, mpaA, sodB, tyrS, yphH, roxA, mhpF, rluC, glpB, otsA, yeaK, entF, ydaM, paaZ, yagI, flk, topB, purT, ypfJ, fliK, pbpG, nuoN, narY, purR, paaB, yegE, puuB, pflA, thiM, kdpD, bglX, hisI, artJ, ddpD, galS, oweS, stfP, araA, kdgR, hisD, fdnG, yceM, yebE, paaX, ydcJ, yneO, citF,*

		bglF, degS, kefC, zraR, nfi, corA, gadW, sbcC, secD, ppx, lexA, yfeA, sslE, ykfB, glvC, yihF, btuB, dsdC, yadG, lpoA, lldR, gspC, yicJ, ftsE, ytfL, recQ, waaC, fsr, yghB, allS, yhfU, metI, zraS, rmuC, yidH, deoC, ykfC, dcuA, ilvA, ykgA, dcuR, yfiH, hsdR, creC, casB, rpmB, yhgE, crfC, tatC, mnmH, aaeB, ilvC, alsA, glpX, yajG, yhdU, accC, yidX, dcuS, yqeJ, metQ, waaA, yiiD, frvB, phoA, yjjQ, nanT, ycbF, fumB, yiaJ, ugpB, eutG, ygiD, yfdV, recF, lyxK, yibG, yqjI, bglG, ulaE, acrF, mtgA, acs, yheO, waaH, yehX, yidA, bcsG, oxyR, prlC, yacL, yoeF, yidD, atpC, ftsA, ptrB, rclR, tsaE, ydjN, yliE, allD, cysN, treB, murE, pepB, hyfR, ygbF, adeP, cbdB, yjaA, dnaE, yihM, fdhE, yhaV, cpdB, pepD, envC, yihG, acpH, insG, yjiV, mdtO, ybdZ, glmM, metA, citT, cadA, opgB, pgm, rarD, yiiG, crr, livH, mlaE, ahr, mglA, ubiA, yaiW, ygeX, yhaM, bisC, dacC, mnmG, glk, yhjK, yadH, viaA, ybhD, yggI, gsiA, sgcQ, hcaR, obgE, yjcC, msrA, yheS, yhfS, yhjH, barA, yidC, ydeP, endA, yraK, gcd, adiA, ylaB, gudD, yjfF, thrA, ysgA, cycA, acrA, yhjD, ppdB, phoE, yddB, murC, yrbG, caiT, yadE	*pyrD, yniC, exuT, kdpB, djlA, cfa, potE, ycjW, mfd, dsdA, gfcE, atoS, umuC, menD, nuoE, yejL, ypdF, yhaK, yebF, hyfE, ydgJ, yeiB, yehT, cho, potI, nac, flhA, yddA, tyrR, azoR, preA, flgG, ygbE, ydcT, yegH, yeeJ, lolC, nagE, dhaM, yphG, nuoL, rimO, ralR, ampE, yjeM, mrdA, ybjX, wcaK, hipA, opgH, yebQ, ydjE, atoD, tolB, macA, mipA, mngB, yedQ, ynbA, plaP, hyaB, nudI, ydhT, glpQ, dsbG, rutA, rzpR, wcaM, stfR, ruvB, yecE, rssB, nrfA, ychA, astD, wcaJ, uraA, sapA, ansP, yfhM, yiiQ, yneJ, nagZ, torZ, yfbS, adeD, paaH, ybhG, ttcA, ybaP, gdhA, ybdR, yahJ, yebT, yeiH, ynjE, psuG, prmC, yejM, yccJ, uidC, dicA, ycbJ, ddpF, mdtB, grxA, cof, metG, yehB, mdtA, selD, gtrB, yajR, pykA, ydhP, ybiY, dgt, yeaC, ynbC, hycI, ybiB, lolE, yahK, wcaG, ygbK, rcnA, rlmL, uxaB, umuD, ydcP, ydfI, lpxH, uof, tsgA, yegK, sanA, yjhY, rhmA, entS, fadR, ydcU, nfsB, moeB, ygbN, yfhG, flgD, ybaA, pepT, yccM, yfaS, hycC, dcp, cpxA, yoaD, narX, yfaL, hyaA, smrA, lsrC, oppC, gpr, glxR, yeiI, nei, ydiP, arnT, pykF, rpoD, hydN, hypD, sucD, potH, nuoJ, ltaE, citB, ydhK, flhB, panE, ygaH, dapA*
(3) GSE20305	16◦C 10 min	*ynfN, cspI, cspH, cspG, ynaE, ydfK, pinQ, cpxP, cspB, pinR, cspF, lpxP, hokA, ymcE, nsrR, bluF, opgE, fepD, gnsA, rhlE, yebE, serT, yjaA, sugE, ycgX, arpA, ppdD, fliR, yfaH, trpT, insJ, ydfR, gfcB, fliQ, bssS, tpr, gfcA, galS, yehR, rttR, yeeY, iclR, dinD, yfhR, yejG, dinG, ybaN, aslB, brnQ, spf, sibB, ydiE, yafU, yebS, rzpQ, essQ, rydB, fliI, ybfP, setB, mgrB, dgcZ, yecT, rrrD, maa, yebO, proM, yaiA, yeiW, tfaR, dkgB, yeiB, thrV, wcaD, yigM, argU, yneF, yabI, yafT, proP, ybaA, yejE, opgC, aroM, pheU, yqfA, pheV, flc, rrrQ, yedQ, valV, fliH, insK, ypdA, ppdA, infA, rsxG, ygfF, mtlR, fadM, flhA, ybgD, fepG, wcaE, yzgL, essD, ydaG, rsxA, bglF, yccM, entS, wcaF, wzyE, eptB, yrdB, valW, yfeD, ldtC, bluR, ybcJ, sokB, nepI, ftsX, ndh, rhtC, ygbE, ydhI, rmuC, fliO, omrB, sdiA, ydgI, yecJ, tomB, yohO, rseC, raiA, deaD, ybhS, ynaJ, leuE, yjcB, yadE, rem, wcaB, rcnR, alx, ubiX, ycaL, fecR, uhpC, yieE, ydfZ, flhE, tusE, zur, wcaA, ycfJ, yhcG, lnt, mdtG, yfiM, yegE, pphA, yedA, fliP, racR, yagJ, mzrA, yccA, yfjV, aspT, yfiR, hsrA, ilvM, yadI, aspV, rimP, yhcA, rdlD, aspU, yegP, holB, lgoT, ydgK, fliG, yciQ, ftsI, gntR, mdtI, fucR, ybaV, cspA, hofB, npr, ychO, argZ, yeeO, queE, yajR, wzxC, yhbE, yifN, argY, ydfU, rnlB, ykfJ, argV, rpoH, nlpI, dsbB, tfaQ,*	*plaP, argG, cysP, yqeI, ybhC, yqeJ, yeaD, potF, pyrI, pstS, copA, upp, purH, yfdC, appY, csgF, stpA, fadE, pyrB, artI, agp, csgE, argD, metN, mdaB, artJ, hemL, flhD, yccJ, yfhH, ybjP, pntA, yahK, yddG, ppsA, moaC, yghG, mtlA, talA, yiiS, yniC, tauA, moaB, ushA, yhjC, pyrC, ygfZ, fixX, cfa, pta, rtcR, atpF, purB, argS, yffR, pflB, artQ, tktA, yfcE, yihI, metJ, atpE, metC, nlpC, gcvP, guaD, yfcD, yagU, metF, hlyE, groL, murQ, cysN, moaD, yiaJ, ilvB, potD, argC, yeeE, cysD, glpF, grxB, putA, ycgR, dnaK, queC, ybbN, nanM, purC, csgD, gltA, thiB, mppA, mprA, ompT, yedL, mtfA, yfeH, yqhD, yqhC, feoB, yebK, feoA, ppsR, metE, pstC, matP, bioB, eutB, hisJ, ycaK, slmA, tufB, ydaT, dcuA, cysH, tufA, dkgA, panD, yhaH, ydhS, deoA, yiiQ, guaA, fabB, ilvC, rimJ, malP, aceE, yfbM, hemB, deoC, ltaE, tktB, dnaQ, yfdT, pspA, rnb, yddW, yggX, dfp, poxB, dut, hemN, nadA, codB, panB, carA, lpoA, ldtB, pfo, rplF, nanS, pdxJ, mobA, nuoB, degQ, hycG, narL, moaE, aroD, ymgA, ybjS, uxuB, speA, puuB, ansP, ldhA, trpE, prs, pepA, prlC, manX, msyB, ackA, potG, ybjD, pepD, gabP, fbp, fruB, casC, proB, galU, opgG, ybiU, rpoA, pykF, solA, gatY, yhdZ, bcsE, dmlA, uxuA, icd, hycH, nfsA, proC, glyA, yhdW, yedE, yajC, cyoB, dacA, gcvR, rbsD, norR, ogrK, rimK, carB,*

		yaaX, gltL, araJ, acrZ, ydhK, bcr, ecnB, argQ, yqjA, ydjM, yhdP, ydbL, pspG, polB, yeiE, tfaD, fliK, priA, yoeF, hofC, queG, fepB, gspO, ycdZ, pqiA, smrB, yjeT, yafK, narX, yiiX, yqhA, gsiA, asmA, glpG, fimD, mscM, gsiD, pgpA	*kefF, ygjP, frmA, infC, moeA, artP, glmM, oppA, tsx, yqeF, aldA, trpS, dapA, gsk, ydcF, cbpA, ymfI, ybjC, yciW, rplX, nuoJ, rpoD, katG, thrS, xylB, gph, yhhX, rplE, gcvT, valS, ybgF, pfkA, dcyD, metB, ygiF, bglX, gltI, zwf, ansA, serA,*
	16◦C 30 min	*ynfN, cspI, lpxP, ydfK, ynaE, pinR, pinQ, cspH, cspG, ydfR, cpxP, cspB, mdtI, rrrQ, cspF, yjaA, ycgX, rzpQ, yebE, ymcE, bluF, arpA, dgcZ, nsrR, gfcB, rhlE, opgE, insJ, gnsA, trpT, eptB, mdtJ, essQ, gfcA, ldtC, bssS, hofB, insK, setB, maa, ychQ, degP, deaD, yaiA, puuP, bluR, prmC, sugE, aroM, ansB, essD, torY, puuR, rydB, yhcD, iraM, rseC, dinG, ppdD, yhcA, mzrA, tomB, ldrC, yejG, puuD, dkgB, puuA, alx, dinD, nlpI, aslB, ldrA, yabI, fepD, yebO, rlmB, raiA, yedQ, rnr, yjfJ, hofC, yebS, tfaR, bglJ, brnQ, dtpA, yneF, serT, yfhR, tfaQ, ldrB, rrrD, yieE, proP, fepG, yjcB, argU, fau, yihF, yafU, gspK, iclR, yddM, yehR, nepI, sokB, yqjA, rsxA, lgoT, ydgK, srkA, proY, galS, yegP, recF, tpr, ydfZ, ppdA, ypfG, valV, bglF, yeaL, yhjG, yagJ, valW, yjfK, prfH, fliQ, yccA, rttR, yfcU, yhbE, chrR, ydhI, ybfG, ecnB, gltW, gltV, cdaR, ybfB, rem, ypeC, waaH, yfiR, ybaN, fliR, gltT, yecJ, ebgA, lgoD, yfgJ, spf, rpoH, yrhA, tqsA, gltU, ybgQ, ebgC, fepC, yadE, yadI, yafT, yaiY, entS, ssrS, ypdI, yedA, pqiA, tsgA, yfaH, mdtG, yaiI, puuC, opgC, ndh, ytfK, rlmG, rprA, ybfP, ybaA, pspG, uidA, yccM, tusE, ilvM, rseB, ydhK, wzyE, ygaY, ybgO, ileU, yhcF, yeiB, ykfJ, uxaB, ydgI, yeeO, yfeS, osmB, ileV, ileT, cspA, yrdB, rmuC, proX, ycbU, yciQ, yzgL, yqiK, yebQ, yjcZ, rimP, uhpC, fimD, yaaX, gspJ, narX, ydiU, rpoE, proV, ftsX, lexA, hsrA, obgE, yaiE, dinF, ydfU, yhcG, yhjJ, rybB, higA, rnlB, tusB, yjfI, yggN, fdoI, alaE, ygfF, chbC, yjeO, yfeK, gltL, ycfJ, fdoH, bcr, rhmR, yeiW, yfgI, yejH, rhtC*	*cysJ, argG, ilvC, artJ, metR, argD, cysP, metN, argA, plaP, pstS, pyrI, ppsA, yeeE, ycaC, ybhC, cysD, potF, pyrB, yeaD, argC, metF, cysN, tauA, upp, mtlA, guaB, uspD, pntA, ydjN, moaC, ppsR, flhD, yiiS, yfdC, moaD, potG, nmpC, pstC, yahK, yqeI, yciW, yhaH, ilvB, yagU, cfa, metJ, ridA, cueO, fabB, carB, rimJ, cysH, moaE, gatY, ybjP, mtfA, yddG, wrbA, artI, argI, metB, ompT, metC, codB, moaB, glmS, bioB, rbsD, artQ, yccJ, fadE, purH, mepH, minD, ygfZ, pfo, pta, argB, ycgR, aldA, copA, ybdL, ydcP, thiB, yghG, trpE, ltaE, hemL, mdaB, nuoB, gdhA, ybjS, cysK, malP, yfbM, katG, yihI, norR, groS, ptsH, dmlA, yceH, osmF, dcp, ybaE, nupC, katE, cysI, deoA, yiaJ, purD, pgi, tktA, iaaA, guaD, yqeJ, hipA, yjiT, lysC, adhE, serA, solA, yqhD, argT, rnb, dcuA, yahA, glpK, yffR, nlpC, pyrD, carA, ycaK, mglA, ushA, nanS, proB, modF, yagE, lysA, argH, hisJ, asnA, trpD, flu, feoB, arnC, pyrC, slmA, bglX, nuoC, nlpA, dapB, arnB, frdA, tpiA, ldhA, pspA, pflB, glmU, guaA, glpF, yedL, argS, metE, trpS, grcA, yghA, cohE, hemN, dut, yddW, yfhH, feoA, ydcK, ybhG, potD, glpT, kdgK, murQ, yniC, glnA, yghX, rbsA, iraP, lgoR, gcvP, srlQ, aroD, metI, gapA, minE, ptsI, queC, htpG, yiiQ, yeeD, mlc, flgA, glpA, nfsA, tsx, icd, putA, mglB, yfcD, dkgA, eutB, dmlR, panD, ybiH, ybjD, ydiP, fbp, recE, ygiF, uxuB, yfcE, ybhF, yceM, dhaL, pbpG, yehT, pdxJ, asnB, gabD, tpx, uraA, proA, panB, kefC, trxB, pepA, ybdH, yfbT, ptsG, aroF, fucU, mprA, dfp, mobA, artP, csiD, gatZ, sanA, pykF, ybiU, rimK, cysQ,*
	16◦C 90 min	*cspI, ynfN, lpxP, ynaE, ydfK, pinQ, pinR, cspB, bluF, puuA, puuC, puuR, puuD, bssS, bdm, cspG, puuP, puuE, deaD, rydB, mdtI, proV, proX, rrrQ, proW, cspA, sibB, rzpQ, dtpA, nlpI, ycgX, cpxP, eptB, ydfR, puuB, mdtJ, yebE, fepG, osmB, rhlE, yaiE, raiA, ypfG, rlmB, yaiY, ypeC, dinD, aroM, mtr, yeiE, fepC, ycfJ, gfcB, nsrR, yaiA, hisG, lgoT, yeaE, proP, yehR, yneF, pgpB, sugE, yaaY, nrdE, dinG, mdtK, ykfB, bluR, crfC, ykiA, entS, spf, brnQ, nrdH, mzrA, yagJ, rnr, gfcA, yccA, sra, uvrA, shiA, serS, gltP, ydhC, essQ, yqjA, yeeY, nepI, nrdI, yieP, yedA, fkpA, yecA, yciN, ydiU, ansB, hisL, yjcB, yejG,*	*metF, ilvC, cysN, nmpC, metK, cysH, plaP, cysJ, codB, upp, pyrB, pyrI, cysD, metR, yeeE, argD, guaB, argC, artJ, carB, argG, ompT, gatY, cysI, ydjN, ybhC, pstS, cysC, metN, flhD, purH, purL, carA, appY, xanP, fabB, ridA, yciW, yagU, aldA, ycaC, pyrD, ilvB, argB, purM, csgF, potF, borD, argA, cysP, cysA, csgE, cysW, yahK, ybjS, purN, yccJ, purE, flhC, gatZ, yeaH, poxB, purF, gatA, yghG, pstC, argH, yeeR, thrA, tktA, potG, mtlA, ptsG, nuoJ, pyrC, glyT, thrB, nuoI, yqeI, talA, uraA, gatB, malP, gcvP, nuoH, metE, ycaK, wrbA, glyA, ilvN, suhB, pntA, rnb, livJ, pstA, purT, glmS, treA, thiB, cysU, clsB,*

		cspE, yeeX, kefB, maa, nadB, ychQ, alx, yhcN, greA, rlmE, tomB, flgK, yobA, zur, rseC, iclR, dgcZ, recF, nudK, ampE, yabI, fdoI, yjaA, ygaY, ymcE, rcnR, ymjA, sulA, yrdA, hisD, ydhI, yibF, fdoG, cnu, rpoE, mgrB, rcnB, truB, dmsD, fdoH, sdiA, ycjG, ytfJ, yfiR, yedQ, tusB, sibA, alkA, yfeC, galP, uxaB, ppdD, dinF, yaeH, rnd, kefG, yjcZ, infA, ldtC, smrB, ldrA, ycjX, pck, flgL, dadA, yhhW, psiE, ldrC, yfeD, mlaF, fepD, rbfA, essD, tqsA, ydiE, yeiG, aphA, zntA, ndh, yfaY, fadR, rem, ybbC, ybaN, ryfA, rseA, yniD, sibD, yjtD, rmuC, lexA, sokB, mntH, rsxA, galR, aroG, ampD, feaB, yifN, glcD, pnp, ebgC, yrbL, ydgK, ebgR, yebZ, ilvY, srkA, rdlB, ybbA, rna, ybeD, yeiH, rof, dcrB, bamD, smpB, rnk, ygaC, yeeZ, rpoH, yddM, waaH, yegP, hisC, fadD, rcnA, pepQ, gntK, ydcZ, yqiK, yebG, chbB, yebS, yfbV, yfgJ, ycjF, tsr, mnaT, tldD, yieE, obgE, nrdF, ssb, sbmA, yadI, hisB, yigM, csrD	*nuoK, tsx, hchA, nuoF, yeeD, glnK, artQ, gabP, nuoG, gmr, rbsD, yeaD, ghxP, pfo, dusC, flu, argI, purB, yciG, prs, livF, grcA, leuQ, eutC, csgD, tktB, thrC, metA, ycaD, leuT, lysC, thrT, leuV, gatD, ybaE, gatC, yddG, artI, leuP, tauA, katG, moaC, pntB, agp, arnC, eutK, yjgL, nuoE, yagI, uhpA, ais, ftnA, yobD, yehT, cvpA, moaD, yciF, ptsI, gsiC, eutL, sdhA, glpT, gabT, argS, dcuA, uspD, metB, yahA, ompC, purC, yohF, livG, yiiS, ydhS, ybcH, potD, rlmI, metI, thrU, gcvT, trxB, recG, intS, gsiB, gabD, livM, yiaJ, gsiD, tyrA, cfa, feoB, purK, narZ, msyB, narY, ycgB, ltaE, yfbM, osmF, nuoL, aroF, folK, nanS, yiiF, bioB, sdhB, thiQ, lhgO, opgB, moaE, cysK, arnB, manX, fis, mtfA, gtrS, glmU, gdhA, thiH, emtA, pbpG, dsdC, fadL, yqeJ, feoC, amtB, sucB, metL, nuoB, eutR, pstB, bioD, yddW, sdhC, potH, frdB, manY, ptsH, sucA, sdhD, yraQ, bioF, ybjT, nlpA, marA, glgA, patD, bioC, metC*

Table S4: Top 250 up or downregulated genes in each study/sample in nitrosative stress

GEO study accession	Subtype	Top 250 upregulated	Top 250 downregulated
GSE60522	DPTA 10 min (No releasing compound)	*norV, norW, ytfE, adiY, alaE, hmp, adiC, yohJ, hcp, yohK, ybiJ, yhaK, bhsA, hcr, ldhA, grcA, ybfA, ndh, cydA, ygbA, yhhA, soxS, mntH, uspG, cydB, qmcA, feoC, mqsR, tehA, feoA, bssS, sufD, yfdY, sufS, ypfM, yjjZ, feoB, ychH, yjgX, copA, sufA, nrdD, sufE, grxA, sufC, dps, sufB, psiE, yohC, tomB, yhcN, hslJ, cdd, mntS, ybbJ, yhfG, ychE, garP, mdtH, zntA, yneM, ynfM, bfd, ybgE, ygiD, nirB, ybjM, soxR, eco, sstT, ybhG, yfdT, uspB, yidK, ybjQ, torI, yccT, yeeY, nsrR, yccM, ybcW, ynfD, asnA, casE, tehB, appY, yedK, yrbL, ilvL, ybiH, fic, dinJ, lafU, blc, ykgE, yecH, zur, bssR, iraD, ygfS, yhhW, ahpF, uidB, mokC, yqaE, yibI, ymgE, ybhL, fruA, mqsA, ykfI, ilvX, emrD, ycfJ, yjfN, eutR, csiE, yjcB, smf, ydaW, yjaB, nemR, ybhF, rnr, nrdG, yfhM, ytfL, gadX, csrB, nrdI, ydcK, ryjB, ascF, rof, rayT, mscL, yfiR, yeeP, ygbN, frdC, nrdH, fes, dtpB, yodD, yacL, smg, ilvM, clpS, pdhR, fepD, chaB, ypfH, yrhC, gltP, flgM, sseA, mgrB, ydhX, yafY, yfcI, artJ, dkgA, yjiY, yedR, ybaV, bolA, hofB, ybdF, xisD, ygiN, slp, gspO, narH, ompX, yqiJ, ydcY, amiD, yedY, ilvG, yffL, yibT, ydiE, eutH, ybdM, yqjI, yqhD, fhuF, pmrD, yahC, clpA, yddH, yecA, yifB, qorA, yaiT, rimJ, osmB, ybgS, focA, panE, yddM, cbpA, focB, yhcH, mhpF, tauD, fsaB, yhiI, rhtB, dtpC, fecI, yhcG, arcB, cbpM, malG, uspE, yiaT, yeaH, pgpC, yeaG, narG, hha, sbmC, sulA, ygbF, ydgD, exuT, ybeM, ydjA, ybdZ, leuO, yaeP, ycgB, aidB, argI, yfiL, mhpB, yafE, proP, hspQ, yaeH, ybhS, exuR, dgoK, yejO, yjcZ, yoeA, amiC, insM, xylH*	*metF, metA, purE, mglA, metR, metB, rnpA, mmuP, purM, metN, sdhC, metK, xanP, flhD, ybdL, pyrD, fadB, cyoA, purK, fliE, mepS, metL, plaP, fliR, hisR, fliQ, fadI, dusB, ampG, thiP, ydiY, purH, rpsT, yibQ, valZ, pyrC, yciH, yliE, lldP, yigI, codB, argQ, ydcI, purF, hflD, ybjE, fis, rpsL, fadL, rlmA, ndk, yiiX, yceA, prfC, ygiQ, pstS, purT, ibaG, mmuM, mglB, uraA, yncD, recQ, pyrF, suhB, rimO, rsxA, ybdH, leuP, gtrA, queD, efeO, sdhD, yoeG, aceB, yecJ, yeiP, cvpA, upp, apt, queA, metI, yeiB, purD, lysZ, flhC, lpxH, mrdA, lysA, kgtP, potA, alx, tsgA, yqeF, nepI, rpsG, speC, mipA, csgD, purR, eptC, rsxC, rplM, fklB, rbsA, puuA, fiu, mreC, purB, puuP, tsaA, rph, gcvT, argV, ybhC, purL, evgS, thiQ, bioD, adk, metC, hsdS, glyA, efeU, mltF, carA, yqhA, stpA, fliJ, rpsU, sbp, codA, waaH, yneE, potB, rluC, gtrB, yidC, rsxB, ycaO, purN, yhdT, potC, ybiT, ymdA, gcd, rplC, rnb, trmN, metJ, tsx, yecE, yahA, tig, yibB, rpsJ, yhgF, metQ, valT, rpsA, prs, thiB, lysP, typA, pcnB, mhpR, ygiM, lipB, frmB, aceK, can, pheP, yedV, waaC, tsaB, cysJ, potD, rpsI, mqo, mnmA, rarD, glyT, gtrS, recR, aroA, emrA, speD, yadS, rpmH, murA, arnC, nuoC, yoeH, tolQ, bluF, cmk, hsdM, glpF, ydcP, fliP, trxB, pstC, oppB, waaL, tyrP, uhpB, tdk, argX, gatA, wecB, fliI, aldA, serC, mglC, uhpC, ycgZ, rlmN, cyoB, rplD, prc, accC, thiI, dacB, yegQ, pth, tgt, ydiJ, ubiX, nuoA, yccF, argZ, tolR, miaB, ynjE, tsr, recG, yciA, fumA, ivbL, efp, purC, ispA, rlmH, fabA, pqqL, gluQ, yddB, eptA, dacA, gpt*

Table S5: Top 250 up or downregulated genes in each study/sample in antibiotic stress

GEO study accession	Subtype	Top 250 upregulated	Top 250 downregulated
(1) GSE56133	Amp 1hr	*uhpT, rprA, ycfJ, cysH, ymgD, hslJ, osmB, yciA, ymgG, ygaC, ycgZ, gnsB, yeeE, yeeD, ypfG, yhdU, rttR, cnu, yedN, ydiY, ycgX, ygiM, emrE, sixA, yojI, cysJ, rsxA, efeU, opgC, marR, yceA, micF, yncJ, stpA, ydgK, leuE, ydgI, trmN, yddA, ybfE, nhaA, murJ, ydeS, rsxB, yaiY, ariR, mdtJ, gtrA, lpxT, queD, tpr, holD, cysI, mliC, rluC, cspF, dgcZ, yliE, dusB, ybgC, mgtA, rph, infA, dusC, ydeT, apt, yfhL, ygbE, tsaB, ibpB, plaP, elyC, ygdQ, opgE, yggI, ydiE, ycdZ, wcaE, ybjE, gpt, tolQ, ybjG, adeP, pyrF, rlmA, yihG, secG, ldtB, nudE, ivy, pabA, rfaH, yjaA, holE, mreD, ubiX, rnpA, yigI, iap, cspI, yegS, pheP, marB, omrA, yeiH, epmA, degP, rseC, ampG, iraM, yhbY, fis, bluF, ybjO, yafK, folA, glpE, essD, tyrP, yhjJ, ibpA, thrL, fliR, rlmG, yncI, mdfA, yecT, queA, rcsA, mntP, pspG, tdk, rimI, ydjN, cysD, arcZ, nrdF, nrdI, rlmH, mdtI, ymgA, suhB, lipB, pth, ydcX, yedJ, mioC, yjjA, yfeY, ymjA, rpsU, aroH, rnhB, xseA, renD, yebO, yfeS, gluQ, pyrD, glsB, srkA, nrdH, ppiA, ibaG, ydhJ, mltA, mreC, fliE, yhbE, yhdT, wzzB, tsaA, ybcJ, rnd, srlD, yehH, purE, ydeJ, mltF, cusR, cspB, cdh, ybiV, yneE, fecC, greA, ecnA, entD, ycaD, fxsA, ytfP, atpI, cysP, ytjC, yeaY, mepS, rlmB, mutY, ratB, rpsT, pheM, yegD, ftsB, emtA, mltD, menA, maa, fhuD, bcr, yahA, rimO, ryhB, thiB, yncD, yiaD, udk, yahM, yjgM, omrB, aroK, proY, yagI, acpT, emrD, ydeR, tusE, glpG, ttcA, recQ, fadD, trmI, trmA, yfcL, potA, ydgC, yghG, acrZ, ygdI, dbpA, tcdA, ldtC, cutC, rsmF, rep, yqhA, ycgN, tsaC, wcaA, hcaR, yfaZ,*	*tdcA, ansB, ghoS, ycbJ, bssR, gudP, ydhY, nrfA, ysaA, napF, garP, napD, abrB, ompW, yjjI, narK, lgoR, yehD, hypB, tdcB, yniA, hybO, melR, gldA, garD, nikB, dcuC, yjiM, dtpB, cdaR, ygjR, mocA, napA, yhbU, yahN, yqeC, ybaE, frwA, wrbA, ynfE, ynfK, narG, frdA, yjjP, ychH, ravA, atoS, yqgA, ttdR, yecH, hypC, gadE, nirD, yqfA, yadI, yehC, gpr, ybeL, phnE, ydbC, pka, pepT, bioF, uspE, sgcX, dinD, ccp, sbmC, feoA, yfbS, ycgB, uspC, hcp, fruB, uxaB, zraS, preT, yfbM, yaaJ, frwB, pyrI, melA, bioD, chpS, yccM, gpmM, csiE, narH, frwC, ygdH, bioA, ydjY, yaiV, pyrB, yahC, grcA, uacT, srlR, ydiL, araC, narJ, uspG, putA, dmsB, hybA, ybgA, chpB, caiF, dcuB, yccJ, yhbV, garL, yagU, adiY, cstA, dhaK, nrfB, uxaC, fucP, yidE, fsaA, murQ, ybiA, glk, frdB, hybB, nikA, aldB, carB, alaE, araF, napB, rayT, rcdA, glgS, napG, sulA, ybjD, yjhI, ftnB, qorA, mmuM, lgoD, rhmR, malI, mrr, dmlR, ycaC, ydcH, yieP, yidL, galS, mtfA, manX, yidF, ldtE, acuI, hcr, ydhV, yjfN, frdC, focA, hybC, ynjE, ttdA, yhbS, feoB, smg, malX, fucI, dmlA, yejG, ykgE, glvC, yhhY, yaeH, blc, galE, recN, ampD, ykfB, yhbT, fucA, yfjX, yjgZ, mlc, btuB, yfdY, ybaP, ydeN, yfaH, napH, yagN, torT, yafZ, yjhH, allC, dgoK, allD, fucK, cydA, yjhQ, gudX, gspO, ilvC, ybdF, yfeC, yqeB, yhhX, smf, fsaB, srlA, hypD, purD, tdcR, araB, dctR, yjcZ, tdcC, yahB, arnF, idnK, ygeV, yeaG, mglA, nrfC, cbpA, yfcC, dld, rihA, croE, ppc, fetA, pspF, pfkA, ygcW, lsrA, ygeK, talA, frsA, xylF, yfdN, ytfQ, yacL, insH25, mdtE, ydiM, yfeO, cydB, glpB*
	GENT 1hr	*ibpB, ibpA, mgtA, puuD, pspD, ydeS, fxsA, ydeT, mdtJ, yjhQ, bssS, marR, yigI, pspC, puuC, ycgZ, ybeD, pspB, pstS, yjhP, zntR, soxS, rttR, phoB, mdtI, ycjX, hslV, ycjF, soxR, ynaE, puuR, nhaA, marB, ymgA, astC, glcC, hslR, zntA, hslO, allA, cnu, ybjG, pspA, gfcA, mqsA, yncJ, yahM, yjhD, trxC, ybfE, ybeQ, yhdN, dnaJ, ygeQ, bhsA, hcaR, mutM, cspG, yiiX, tpr, dgcZ, hspQ, ilvM, gcl, clpB, ykiA, lipB, ilvG, ydfC, fadD, fadE, cspF, mhpR, ybbN, fadB, cycA, ygbJ, corA, yqaE, hyi, ldhA, puuB, yqiJ, yjeJ, ydjM, sixA, marA, ydgK, ydbA, yfdK, phoR,*	*narK, napF, gudP, yhbU, malK, nikB, bssR, ghoS, ynjE, malE, garP, yjjI, lamB, malM, tdcA, nrfA, ansB, gtrS, nuoM, ydeN, ysaA, carB, malF, abrB, napD, malG, narG, hsdS, atoS, flhE, yecR, feoA, uraA, ycbJ, flgI, fliJ, nuoN, ribB, feoB, yddB, fliK, flgF, tdcB, garD, fliI, ymdA, rsxG, nrdA, glcB, ravA, yehD, nuoH, codB, hypB, adiY, frwA, nuoG, cydC, pyrB, nuoL, flgJ, pyrI, nth, potC, nuoK, purL, nikA, carA, fliG, fhuA, ygdH, waaL, yjiM, oppF, focA, ycaO, recB, lgoR, cdaR, glnD, recD, napA, bioF, yfjW, eptC, nuoI, nuoJ, ybaE, hypC, rsxE, flgH, potD, oppD,*

		ybfG, ydaG, gntP, ubiX, maa, rlmE, mqsR, ecnA, yjhE, higB, rsxA, uxuA, sfsB, yjaH, stpA, yhdU, htpX, yhbE, holD, ydeR, yjgX, htpG, yebO, yciE, nanC, erpA, ydfA, yciG, yodB, yciM, yciF, nhaR, iraM, argT, ygeN, intF, mdfA, aroM, symE, ydhI, rhsJ, smrA, ybcW, ybfC, ygaV, hslU, insO, betI, thrL, asnC, xapR, ybiJ, yhfY, renD, bdcA, rsd, ariR, alpA, phnC, rutR, dicB, yidB, rfaH, yciS, tomB, yadC, rhsO, cmtB, ygaP, yhcN, uxuR, gcvA, pgaD, yjaA, rnpA, yabP, folA, tusA, ytfP, yffS, yedY, betB, ykgJ, grpE, yhbP, mdtQ, rpoE, queD, yadM, yhcF, glpE, ygiM, yffB, ydaS, ydjF, yfeS, yfhL, dnaK, mioC, mntP, emrE, yabI, asnA, dinD, yhaO, uxuB, ybfD, fadI, yafE, yfcI, fadM, leuA, rpoH, gfcB, leuE, rybB, pfkB, yciA, ybgC, yagM, rcnR, sstT, ychE, acs, mlaF, dnaT, ydhJ, ylbG, mntR, tfaS, iscR, insJ, yciW, phoA, cspH, rimI, pabA, ydiZ, yegZ, chbB, yjiR, trmN, bamE, degP, yjcB, yagK, yjgH, tqsA, mngR, gpt, hchA, yejG, yehH, yccX, ydcX, atpI, yagI, pth, afuB	*speD, pqqL, murF, napG, sapD, yqgA, glcG, menE, ynfK, purD, flhA, ydhY, dcuC, sapF, ydiJ, nrdD, grcA, hybB, atoC, rsxD, oppC, fecE, gcvP, fucK, codA, bioA, glcD, malS, glcA, flhB, rffH, oppB, nrdB, flgA, hsdR, yehT, nuoF, yfaE, mraY, ccmE, melA, malT, yfbS, murD, gltB, fliF, kup, purB, ygjR, cheZ, garL, fhuF, yjcZ, cydA, rihA, mutS, aceE, mukB, fliQ, hybA, ydjY, mcrC, napB, flgG, fliN, malQ, nrdG, narH, rnb, ynfE, mrdB, yedF, gltD, efeO, yegE, narI, uhpT, fliP, rffG, glnG, ygfF, fliO, fliM, napH, efeB, narJ, trkA, ydjX, potB, menC, sapB, nirD, rluA, frdC, yhhJ, menD, frdA, fliL, cyoE, evgS, cvrA, glpC, melR, frdB, cheB, yidF, wecC, gldA, fecD, cyoC, ybgA, gadE, fecC, menH, rcsD, sdaB, cydD, ccmD, artQ, frdD, ybgE, yccM, opgH, flgB, clsC, dxs, cyoD, fepC, spoT, yedE, fliT, prmA, glmS, emrB, hybC, ycaC, ebgC, ygiS, yehC, fliS, flgC, nuoE, accC, cheR, cydB, yheT, yfjK, uvrC, rsxC, fecB, ttdR, artM, cheY, thiI, pheT, thiQ, ydbC*
	Kanamycin 1hr	*ibpB, ibpA, ycgZ, ydeS, fxsA, ydeT, mgtA, ariR, mdtJ, ycjF, iraM, ybjG, ycjX, ymgA, puuD, ydeR, yeeE, bssS, nhaA, ymgC, ybeD, hslV, pstS, ykgH, yeeD, cnu, leuA, marR, gnsB, yigI, dgcZ, mutM, yhdU, cysP, yciA, mdtI, emrE, fadD, micF, dnaJ, ydeQ, clpB, ygiM, astC, fadE, yncJ, leuE, hslR, ybbN, hslO, soxS, rttR, rsxA, ybfE, yebO, nudE, yjiR, ygbE, marB, yehH, ycgX, hcaR, phoB, sixA, degP, elyC, rlmE, yahM, hslU, lipB, astD, trmN, ykiA, ybiV, hyi, ydgK, htpG, bluF, ybgC, puuR, yfdK, thrL, cspF, ydhJ, mdfA, fadB, yciM, renD, betB, phoR, yafK, nhaR, tpr, cysA, hspQ, holD, cspG, ygeQ, cysH, htpX, yjhQ, intF, marA, lon, yedW, yciS, betI, ydbA, ubiX, tolQ, leuB, soxR, gtrA, cspB, yggI, cycA, rsxB, ynaE, cysU, prlC, rstA, lpxT, yceA, yabP, hisG, zntR, ilvM, cysJ, chaA, yjhP, tqsA, yhfL, yedJ, yhdN, ybjJ, hslJ, ydhI, glpE, ppiA, mltA, cysD, hisD, cysW, grpE, gcl, yjhD, ybjX, pspD, dnaK, ykgJ, yfeY, stpA, rsmJ, queD, lnt, gfcA, ilvG, ldtB, rmf, rstB, tfaP, puuC, murJ, srkA, ydjN, trxC, folA, argT, ybeQ, mioC, ydeP, pfkB, rluC, allA, mgrB, ibaG, hisC, yneE, gadW, aroM, ybeZ, infA, pabA, deoR, ratB, leuC, ymjA, yafE, pgrR, yfgH, ydgC, yedV, pth, ybeY, bcr, ybeX, yjhE, ilvN, mepS, mntP, yojI, gltP, rimI, gcvA, cspI, ybjH, sdaA, mhpR, xseA, leuD, ybbC, yedA, betA, pspB, yhjJ, yhcN, ybjO, yfhL, ytfP, yjeJ, gmr, yfeS, ycbK, efeU, ycjW, ybfG, fadA, cysI, glcC, yncI, pspG, yjgM, ydiV, gfcB, mlaE,*	*garP, tdcA, gudP, malK, lamB, napF, malM, ansB, bssR, napD, malF, narK, garL, garD, malE, ycbJ, nikB, ysaA, ghoS, ompW, yjjI, nrfA, malG, yhbU, hypB, cdaR, yecR, napA, abrB, tdcB, hybO, ymdA, gldA, yehD, narG, fliJ, ribB, dcuC, lgoR, frdA, carB, flgI, garR, fliK, pyrB, pyrI, frwA, ydhY, fliI, atoS, melR, hypC, feoA, ravA, flgA, ynjE, yjiM, nirD, flgJ, flhE, hsdS, uraA, putA, flgH, bioF, napG, melA, flhA, frwC, fliG, ynfE, grcA, nikA, yqgA, adiY, ygjR, ompF, frwB, flgF, ynfK, ybaE, feoB, ydeN, yahN, flhB, gtrS, nuoM, bioA, hybA, fliZ, yidF, fliF, gadE, frdC, frdB, mocA, nrdA, yecH, ttdR, narH, hybB, fliT, napB, yfbM, napH, ydbC, malQ, yadI, frdD, dcuB, pqqL, yniA, zraS, pka, yhbV, yjjP, wzc, waaL, yddB, carA, gudX, fliN, yaaJ, fliM, yjcZ, fliS, gltF, yccM, ydjY, codB, fliL, yehC, cydA, flgG, yfbS, dmsB, wrbA, malS, glcB, bioD, hcp, narJ, flgD, yqiC, ydjX, ccp, fliD, rihA, narI, yfcZ, fliO, menE, fliQ, focA, flgC, nuoG, ynjH, recB, srlR, pepE, rcsA, ptsG, rihC, ykfB, ygdH, flgB, fucK, fliP, cydB, hypD, sgcX, yqeC, ymdB, recD, nuoH, ydiL, fliA, nuoL, tdcR, hybC, feoC, ldrD, fecE, evgS, purD, codA, nrdB, araF, amyA, clsC, pfo, nrfB, flgE, yedF, yihM, elaB, dtpB, malT, yihN, fimF, ychH, pepT, btuB, nuoK, glnL, tsr, purL, speD, pyrL, yiaL, blc, ycgB, ygeV, fliR, eptC, hcr, rhaR, gpr, glcA, uspC, nuoI, nuoJ, cheB, fimG, ycaO, ygeW, fucI, tdcE, kup, katE, mcrC, entE, yfaH, araB, dmlA, yhfK, flgN, melB, cheR, yfaE, ycfP, yfcC, dctA, nrdD, glpB, idnT, srlA, fecB, fdnH, iraP*

		tmpR, arsR, cutC, ybjE, yebE, osmB, gpt, yagI, pspA, omrA, mlaF, rhsJ, cysN, ldhA, feaR	
	NOR 1hr	*ymfM, ymfJ, ymfL, jayE, ymfR, ymfQ, ariR, recN, ymgA, ymgC, ycgZ, cysJ, cysI, yfaE, yebF, yeeA, yiaG, polB, yebG, entC, iraM, entE, ydeM, fes, nrdB, yicG, nrdA, fepA, entF, yegD, holD, yibB, ymfD, paaD, yeiH, paaB, paaC, tusB, paaZ, cysP, ppiC, yliE, rnpA, lipB, paaA, paaE, yciF, yfcE, ydjM, rimI, yoaA, greA, fecI, mutM, paaF, ogrK, glmU, yifK, nrdH, entB, fhuE, fhuA, nrdI, yqjH, ydjN, lexA, yidB, yfcD, fecR, nhaA, yigL, ecnA, tsaA, entH, entD, yigZ, ldtB, fepB, yjbJ, fhuC, yidZ, tusC, efeU, entA, fadB, pfkB, dut, poxB, yciE, recF, yidC, ycfJ, yceA, gpp, yqjI, pfo, ymfE, yigI, elfA, nrdD, nrdE, nrdG, yraH, yciG, nfi, yggE, ubiB, fadL, fadA, yhaK, can, groS, glmS, acpT, fldA, yieP, groL, truA, yqhD, fecA, yhhA, yqgB, pncC, tsaB, yrfG, ilvM, pldB, yffQ, rpoD, ydhC, entS, yhhM, fepD, osmB, kdpA, fepC, yhbE, yncE, cysU, argE, yffR, mutY, otsA, yidR, yeeN, caiB, guaC, fhuD, rpmH, trxB, epmA, nupG, rnhB, paaH, fbaB, ahr, hemX, ynaI, yfeH, endA, lldP, yicC, nrdF, yjdI, yibN, cysW, rpoH, yhjK, fimE, glmZ, glyA, yghG, yhgN, yghA, nusB, gntX, yhgF, yigB, eutK, rlmJ, fepG, yjgA, yeaY, yicR, grpE, mreC, fabH, fadD, yjbG, ygcU, yhbO, hemG, pepQ, nanM, yjdJ, hldD, ygiW, trxA, dacA, ghoS, mscS, otsB, eutC, yihA, ecnB, ydeN, mreB, ubiG, ytfL, mutL, eutL, fecB, fis, kdpC, rhlB, pdxB, murI, fabF, mscM, yciW, gpt, tusD, zraS, dacB, adk, yhjJ, yecM, eutB, ychF, dxr, exuT, ackA, ribE, murA, fabA, yjaG, yeiI, ycgH, glpG, yifE, exbD, yggX, zapE, exbB, yjeT, nudC, argF, yfiF, amiB, gltX*	*zapC, yecR, ycaM, yfeS, ydhY, yeaR, feoB, flhE, ymdA, fruK, feoC, intD, ynfC, yoeA, fliJ, murQ, abgR, ydiV, yqeC, ycbJ, ydiL, caiF, yffB, ccp, ydbA, fliR, yiaB, hisL, ycaN, yhcF, elfC, flgI, ykgF, fliP, ydcL, yfjU, rnlA, ydjY, gatD, narG, narI, yjcZ, ompT, nhaB, ymdB, flgJ, yciT, yeeP, yhcG, eptC, ghxQ, yfiR, glcA, yfjK, preT, flhA, ygeV, yeeL, ykgG, yjhE, ydjZ, yqiJ, flgH, mtfA, ydjH, ydgC, ydhV, yfeW, ydcZ, insQ, essD, ydiP, ykiA, yciQ, eamA, ygbF, gspO, ykgE, fimH, ygbT, ebgC, ycaK, ydjX, yecT, nlpC, fruA, ygfT, fliG, pinR, casD, yfjL, ydbL, yegE, perR, leuE, phoP, gspJ, yfdK, gatR, feoA, feaR, fimG, nrfA, ygiS, iraP, dapE, yqeB, fliN, chaB, opgD, ydaN, rmf, evgA, ynbE, yoaG, hokE, flhB, yoaF, yfcQ, yccM, yfcP, nirD, yniA, yegZ, yohJ, ydbD, yqjA, yfbS, yhbU, ynfK, fimF, yphA, yphF, yhaV, aegA, yecC, abrB, yfiN, nuoN, yehI, yfiE, yciK, ydiJ, yebK, pmbA, yeaX, yfgF, gmr, ygjH, yhjB, fliT, gdhA, yegW, fliE, kch, ydcC, ycbK, insN, ydcS, tyrR, yegT, ynbD, ydbC, ynjH, ynfE, aldA, yejA, ynfA, flhD, yhbV, pheM, yfbN, yceH, yehT, flgF, ansB, hns, ydfV, yceM, nanC, aphA, yfdT, ydaW, yihM, fimD, yfiB, arnD, pntA, yfdE, ldrD, ttdA, yebW, ycjP, livM, envY, yfjM, ghrA, ycdU, gspF, ydcA, yfcU, fliZ, narU, ycjD, dctR, fadR, yhhJ, yjaZ, ygiZ, yegI, hcp, aaeX, ycgI, yeeW, fetB, pgrR, yneO, yiaL, lamB, fdx, ydhI, ytfJ, yecS, nohD, emrB, yjhD, lgoD, fliL, flhC, flgC, flgA, ydeT, napD, ais, ydeQ, ynfB, yqeF, oppF, mug, frwB, yobB, yeaW, yeaM, chbA, ydjG, yihN, murP, yjcO, ygbE, ykfA*
(2) GSE57084	Enorfloxacin	*ymfJ, xisE, ymfM, ymgA, umuD, beeE, iraM, intE, gadE, ymfL, ycgZ, sulA, umuC, gadA, ibpB, ibpA, gadB, yhiM, ariR, stfP, recN, yiaG, nrdA, dinI, yfaE, ymfD, yebG, ymgC, dinD, nrdB, stfE, yibB, yciE, jayE, gadC, hdeD, ymfQ, recX, hdeB, recA, cpxP, yeeA, ppiC, ymfR, sbp, yhiD, rmuC, zinT, yebF, hdeA, tfaE, tnaC, udp, glmU, yciG, tpr, sbmC, mdtE, yifE, ymfE, glmS, dinB, yciF, slp, mnmE, bhsA, atpI, tnaA, ycaC, ykgM, atpC, chrR, slmA, ycgX, rpmH, yibA, rnpA, tfaP, trxA, yieP, yafO, yidI, ydcH, tauA, ssuE, osmB, yidZ, yibF, ghrB, lexA, osmC, yafN, uvrD, tdh, aidB, fadB, dut, ycfJ, mdtF, asnC, ivbL, rhsA, iraD, bdm, yihA, dinF, polB, cho, yifN, hsrA, ysgA, gadX, yidB, yegD, yieE, gyrB, yebE, rph, kbl, yifK, ilvN, atpD, ndh, rsmG, glpF, yidC, atpB, mutM, uvrB, yjbJ, tatC, yafT, dcrB, hslU, rep,*	*feoA, zapC, ydjX, yoeA, pyrI, pyrB, gdhA, ompW, stpA, ymfI, yjjP, nikA, minC, entC, ompT, carB, codB, codA, manX, fimC, bssR, nhaB, yjjI, fiu, thrC, yagU, livG, nmpC, grcA, hypA, hiuH, yegH, manY, thrA, yohJ, feoB, yfiR, cybB, srlR, yfiN, yfiB, yceI, manZ, carA, proV, idnR, thrB, modF, minE, livH, proW, ttdR, nuoN, nuoM, entE, hisG, gltB, idi, minD, napF, rtn, nuoK, proX, hisB, pdxA, trpB, focA, uraA, livF, gltD, recO, creA, tabA, yfjL, efeB, gltF, rcsF, proB, nuoJ, ebgR, hybO, nuoI, ynfK, gcvP, ydaM, blr, hsdR, evgS, ybaQ, hisM, galR, cspG, hypB, livM, aldA, ybjE, hisI, nuoL, aphA, pyrL, folP, nuoG, ridA, pntA, epd, ydfZ, eptC, rnlA, hns, trpA, fimA, ddlB, azoR, rimJ, iraP, murF, yeaD, yjiT, mokB, argP, ytjC, ydiE, ynfC, murC, acnB, rne, hha, nuoF, yhbT, frdA, fes, mraY, era, yceM, cyoC, argG, yeeE, serA, proA, recD, smg,*

		hslV, yrhB, atpH, hemX, hldD, yjaA, bamE, glpK, rpmG, hlyE, yncI, atpA, ubiJ, cyaY, dsbA, ymgD, rfaH, ilvB, ilvM, yieH, rbsB, ubiE, yhhH, dsrB, tauB, rhlB, atpG, ydcD, rbsK, atpF, hemY, ogrK, puuA, yigI, yidQ, ydjM, yicC, bssS, aldB, cspF, ydaG, fadA, ppsA, ydfK, pstS, tauD, glvG, ahr, srkA, yhhA, yigM, ybhB, tauC, pepQ, ydfO, yghA, dgcZ, ynaE, yigZ, yhbO, yebO, yidR, cspH, gpp, phoH, rsmJ, ssb, nepI, ubiB, secB, ilvE, yidG, puuC, ybfE, uvrA, yibL, yafP, spy, soxS, yoaC, yqfA, yihD, ssuA, tatB, yigL, adeD, hemD, ugpB, corA, aslA, grxC, groL, ruvA, gltP, ygeI, eptB, tusB, yccJ, waaF, cohE, xylA, trkH, gfcA, curA, dfp, yidA, yiaD, phoU, atpE, yiaU, rbsR, ruvB, htpG, appY, prs	*caiF, yfhH, pbpG, hisF, nuoE, hisC, rng, ykiA, yhjE, ftsI, mpaA, dld, hicB, cyoD, ghrA, argA, aas, gcd, yfdY, dcuS, yagB, ftnA, flhD, gatD, nuoH, yeaP, livK, insI1, cyoB, pntB, lpoA, yejF, hisH, yrbL, ppk, yjiX, ydcR, yebK, purE, ppc, yahM, alaA, insI4, nanR, yqgE, yhaK, pgrR, yafE, yfcL, gatY, yrdD, rlmD, mppA, gcvA, rluF, borD, ylbG, pdhR, kefF, fecI, cysC, ppx, cysD, yobA, treR, mepS, sbcB, yaiL, nirB, fetA, xseA, modB, murE, valS, lrp, yciU, ybiJ, ycbJ, cydC, yhcF, mraZ, fsr, hypT, purK, ftsZ, ftsQ, ydiH, cysN, cobT, fimD, nuoC, entB, ghxP, ileS, epmC, fruB, yejA, yccS, psiE, pyrD, ycbK, cysP, speC, zur, cyoE, ompF, insQ, yeeD, holC*
	TET	*glnK, gadE, nac, gadC, gadA, gadB, yedL, slp, hdeD, glsA, hdeB, yciG, yjhQ, yjfY, psiF, hdeA, yhdW, ybgS, yeaH, yohC, yhiM, ddpX, gadW, mdtE, sra, mdtF, ytfK, ydhR, ybaY, yjbJ, yjdN, rmf, ydeI, ygdI, glnL, glgS, yeaG, cspD, ycgB, yodD, ldtE, phoH, mcbA, sodC, yebV, yhfG, ydiZ, relB, yjhP, dctR, relE, patA, yciN, yccJ, rpoS, yegP, yedP, ddpA, glnG, yqjE, uspG, bolA, osmC, fic, yjdJ, yniA, hns, ybaT, yqjD, ybaA, amtB, yffR, aidB, yhcO, pgaD, narU, psiE, otsB, ygaU, yhiD, otsA, csiE, hokD, yeeD, wrbA, cbpA, ivy, dgcZ, yehE, ompX, yjbR, yhhA, yiaG, yoaC, yjbQ, ybeL, leuD, yqjC, narZ, yhbO, mgtA, leuC, fucU, csiD, cfa, gstA, phr, uspA, yjiN, ycfJ, yeeE, frdB, ihfA, elaB, uspB, yphA, ybgA, glnA, yodC, ydcK, rcnB, msrA, lhgO, astC, yhjG, slyX, cbpM, pagP, cueR, ynaI, clpA, kdpB, yqhC, artI, dps, ybcM, ybhL, gabP, treF, gabD, cbdA, gabT, qorA, sfsA, glpD, hspQ, ldtD, yhjY, ydcS, yaeH, ybgF, ydiU, fruA, paaY, ycgM, clpP, yddH, sdaA, mlrA, eutL, glcE, rraB, glcF, yiaU, ykfB, grxA, osmB, poxB, paoA, xapR, yhjD, bax, yaiA, kdpD, yccU, gltL, artQ, yjdC, yqaE, fadE, curA, clpX, crr, yciI, pal, ybhB, mazE, yfcG, uspD, ychH, pspG, nlpE, betB, tdh, yrbL, narY, bdm, ybjP, glpK, kbl, arnA, msyB, potF, mazG, ybbJ, nhaA, araC, nhaR, idi, osmY, ycbK, yfjU, ydiH, yedR, ydcY, frdD, yjjU, clsB, uvrB, paaX, sdiA, ogrK, ycjG, flc, fadM, fbaB, cysQ, ygaC, mscL, yohF, mqsA, yecD, yggE, ynjH, dcuA, amyA, yfcI, dsrB, yedQ, appA, paaK, fimH, zapB, ompR, yhhQ, glcB, yidH, yoaD, rssB, nrdH*	*yceI, ompF, ndk, malE, mipA, lysP, flhD, yeeN, nmpC, livJ, yhjE, hiuH, ygiM, treB, icd, livG, livH, dapB, livK, livM, livF, lamB, uraA, degP, malK, yghG, argG, treC, asd, mdtJ, ybhC, azoR, fadL, pntA, ydeN, mqo, hisJ, flhC, znuA, potA, gdhA, gnd, flgC, rihC, pntB, yhjV, artJ, ppsA, yifK, emrA, metC, dctA, potB, metE, speB, potD, cutC, sslE, hmp, pppA, rbsD, yliF, gltF, dapE, argI, rstA, cirA, flgB, rpoE, yncE, ilvC, yffB, fliA, pmrD, pdxK, ybiU, sodA, argD, ycaO, epmA, aroA, katG, emrB, purA, hsdM, essD, yceJ, lysC, pitA, ymfA, sodB, cysM, aspC, yedE, ubiG, yehU, alx, tig, glyQ, yqgB, ygcN, ybiC, yfeK, rseA, plsX, flgD, yahK, yghF, wecA, kup, mdtI, entS, nfsA, gcvT, panF, ompC, spy, agp, kdgK, dusC, eptB, yhdT, yjaA, tufB, rtn, bamD, flgA, yjjJ, thiP, tpiA, yfeY, secD, tufA, ruvC, ttcA, ydfG, tgt, gspD, cstA, yhbE, fbp, rplQ, nadB, ptsG, ydgH, glyS, fliM, thrC, aroF, fliL, yegX, ulaG, fliN, yhjJ, fhuB, iadA, prmA, yeaR, yebC, fhuC, puuA, yagU, gapA, tktA, ascG, ecpA, tsx, yhiN, yahB, yeaL, yehT, tyrA, yfdQ, adeD, ybjL, ybfG, yjiG, thiD, pstA, lpd, yfiF, crfC, ygiL, ydjI, cysC, fabH, arsR, bglX, yqcC, mpl, opgH, srlB, flgF, wecB, dsdA, yagI, pstB, glnS, galS, thiM, nikR, cra, hemB, flgG, nanA, murQ, gspE, gcvH, rpmC, atpB, insO, modA, uvrD, yfaE, idnO, ygfF, gspF, yqjA, mlaE, fliK, ecpB, radA, umuC, btuC, yhfZ, xylG, phoA, yjiK, rsmD, yjeJ, yhhI, tmcA, mrr, eda, emrY, flgJ, ygcW, ydhK, csrD, yihT, yggW, xylF, gfcC, ygcB, yfbP, modB, hemN, yfcA, yraJ, fliS*
(3)	Amox		

GSE47221		*bdm, ymgD, murR, hisG, gmd, degP, rseB, ypeC, wcaG, yaiY, argC, dacD, murQ, yfeW, xerD, pyrB, yagI, sslE, gltB, thrB, ypfG, narZ, wza, thiC, puuC, ydiE, glnG, acrD, phoA, ptrA, insQ, smtA, fdnH, puuE, minC, afuB, bglB, ninE, pqiA, phnM, fliQ, sgbH, cynT, ygfM, ybhN, ebgA, abgB, nfrB, rlmD, ykfC, mpl, mdtN, gfcE, rhaS, ygjJ, ygjI, yiaM*	*xisE, tnaA, ymfJ, umuD, ydjZ, ompF, umuC, waaZ, lamB, recA, fimB, dinD, beeE, yhiM, ymfL, yjcH, recX, ymfM, cstA, stfE, csgD, yhhM, fadE, fadA, acs, glmU, glcB, glmS, yjcC, actP, glcF, yrhB, malT, ssb, yihA, fadJ, jayE, cydD, ybaQ, artI, ivbL, srlD, stfP, tfaE, gor, tatA, fabR, mdtF, ecpA, clpX, hemD, yceO, nrdB, yfgI, cpdB, atpE, ubiJ, def, grxC, yfeD, fhuC, yhhS, yiaT, yadM, yqeK*
(4) GSE37026	Colicin 30 min	*gsiA, gshA, rpmJ, dedD, ynbE, insQ, ychF, pdxK, lptA, panC, truB, yeiI, ftsX, lptD, rpoZ, yfdV, nagC, mcrB, waaP, yejM, trmA, pliG, potD, yheO, rplY, ydfC, frlR, rpoN, rho, htrE, valS, waaQ, dcuC, pheT, pitB, rhlB, yahM, lpxD, prkB, ykgM, murB, guaC, nuoF, nfo, thrC, fdhE, rimP, metG, ytfP, macA, nrfC, gpsA, yahN, eutP, pyrC, cheR, yadL, sgbE, aroP, yidC, brnQ, nrdB, araE, hrpB, yobA, yabP, yhaO, iclR, yqjI, dhaL, secB, ribF, uidR, birA, slmA, yfcD, menA, yggS, hcaC, eutJ, cspA, glyA, ybiT, ligB, ypdF, yneJ, csgB, ybjT, lsrG, sgcA, ynjB, mcrA, ycaL, yehT, ndk, yjcZ, ymfE, atpC, ydhJ, yjiH, yicI, ybiX, recE, ydiP, thiG, insJ, gntT, smf, araD, sdaB, tdcA, yagH, metH, pka, thiB, malT, secG, speD, yfdK, ycaO, ompF, gfcA, maa, yjhH, ycjV, thiH, sdaC, mdtL, moaA, ugpA, fliJ, xdhA, proX, ygeW, ydfE, ryjA, dsdC, insZ, thiF, cysB, ftsP, cysE, ydhZ, yphD, yegU, znuC, ydaG, adeQ, rutF, fsr, zraP, ytfQ, bioD, yciW, xylR, yibQ, cheW, mltA, insO, xylG, php, yiiE, trpE, nrdD, ryeA, symE, fabA, ygcE, ycdU, pgaB, ycjT, exuT, plsY, ygfK, sibC*	*csgE, csgF, flhD, flhC, essD, murP, csgD, csgG, ydeM, yagU, nagE, ebgA, frdD, cstA, gntP, nanS, cyaR, hokB, yafK, ompW, ymgC, ydeE, cmtB, frwC, sgbU, yjfZ, bioF, ytfT, sibB, fliE, fliZ, yeaW, ytfI, agaB, idnT, fliD, chbF, ybcW, ydfO, yihS, ygcW, sibC, ygfK, plsY, exuT, ycjT, pgaB, ycdU, ygcE, fabA, symE, ryeA, nrdD, trpE, yiiE, php, xylG, insO, mltA, cheW, yibQ, xylR, yciW, bioD, ytfQ, zraP, fsr, rutF, adeQ, ydaG, znuC, yegU, yphD, ydhZ, cysE, ftsP, cysB, thiF, insZ, dsdC, ryjA, ydfE, ygeW, proX, xdhA, fliJ, ugpA, moaA, mdtL, sdaC, thiH, ycjV, yjhH, maa, gfcA, ompF, ycaO, yfdK, speD, secG, malT, thiB, pka, metH, yagH, tdcA, sdaB, araD, smf, gntT, insJ, thiG, ydiP, recE, ybiX, yicI, yjiH, ydhJ, atpC, ymfE, yjcZ, ndk, yehT, ycaL, mcrA, ynjB, sgcA, lsrG, ybjT, csgB, yneJ, ypdF, ligB, ybiT, glyA, cspA, eutJ, hcaC, yggS, menA, yfcD, slmA, birA, uidR, ribF, secB, dhaL, yqjI, iclR, yhaO, yabP, yobA, hrpB, araE, nrdB, brnQ, yidC, aroP, sgbE, yadL, cheR, pyrC, eutP, yahN, gpsA, nrfC, macA, ytfP, metG, rimP, fdhE, thrC, nfo, nuoF, guaC, murB, ykgM, prkB, lpxD, yahM, rhlB, pitB, pheT, dcuC, waaQ, valS, htrE, rho, rpoN, frlR, ydfC, rplY, yheO, potD, pliG, trmA, yejM, waaP, mcrB, nagC, yfdV, rpoZ, lptD, ftsX, yeiI, truB, panC, lptA, pdxK, ychF, insQ, ynbE, dedD, rpmJ, gshA, gsiA*
	Colicin 60min	*wcaE, wcaD, wcaF, wza, wzc, gmd, wcaG, ycfT, wcaH, rcsA, wcaI, ycfJ, yjbE, wcaB, wcaA, wcaC, yjbH, yaiY, cpsB, creD, wcaJ, rprA, wcaK, uhpT, wzxC, ugd, yjbG, cbrB, yiaB, yaaX, ydeI, mliC, ymgD, wcaL, ymgG, ygaC, cbrA, hslJ, dppB, ypfG, spy, osmB, ibpB, ygdI, cbrC, yncJ, wcaM, ydeJ, ybdR, yhbO, yiaD, galP, dppF, dppC, yfdC, ygdR, ykiA, yigG, malZ, dppD, ldtC, yciE, yecT, lolA, yciG, treR, rstB, iap, opgC, ybdK, loiP, ydcF, yghA, ydhS, paoA, ibpA, omrA, ychO, pspD, yciF, omrB, ybhN, tqsA, dgcZ, argC, yegS, yeeE, yfbR, rarA, nupG, iraM, yidF, ycjF, lysA, yegL, yaeR, ascB, cysH, pspC, ybhP, pspB, ulaE, rstA, ygiZ, artJ, yebE, glcA, srlA, rhsB, galU, ycjX, cspI, yeeD, ybhS, yhhH, mlaB, ydiL, nanA, fxsA, yhhA, nanT, opgB, rsmJ, ivy, yaiW, ybdG, epmC, yagF, elyC, ygbE, fliR, yceB,*	*yjiY, flhC, flhD, dctR, flgB, slp, ypjF, flgE, gadA, flgD, gadE, caiB, caiC, flxA, glnG, glnQ, nac, flgM, gadB, hdeB, glnP, yafK, flgN, yhiD, mdtE, yfjM, yhjY, fliE, yiaG, fliG, purT, glsA, fliZ, hdeD, yccT, purM, yncE, yeaL, hdeA, yjhP, narP, xanP, codB, csgE, mnmC, yffL, nrdG, mdtF, yjhQ, mdtK, yieP, yahE, pyrC, ycgH, gadC, elfA, glnH, mscS, yfcD, yceK, cbl, yfiB, yeiH, mdtG, moaB, gcd, pppA, fliF, psiF, evgA, csgD, yahF, guaC, ybgA, phoA, insQ, ybfF, codA, glcC, gapC, hchA, yagU, phr, holD, ycaC, slyX, yjiV, hsdR, yahK, ymfI, ypjA, dtpB, nrdD, yhcD, dnaT, frdA, yjgH, yfcE, gmk, mprA, priC, gstA, purN, rutR, ydiV, trmL, yjbD, yahG, cfa, moaA, kdpC, bolA, hybO, ybfE, yoaC, yigI, yjdJ, ydjF, pdxK, casD, yiaJ, fliM, inaA, holE, purH, yicG, mntH, yffM, yjgX, dnaA, stpA, gss, gadX, yraR, flk, artI, hemA, yejG, casE,*

		pheL, pagP, chaA, yjbJ, chbR, ydeS, nanC, pspA, mlaC, yeaO, mdtB, anmK, sfmA, osmC, ycaP, yehX, dppA, garP, marB, ybhR, btuR, ybjH, ybeD, yidL, leuL, mscK, cdd, yjcE, yciK, yaiA, manA, ybgS, yahA, cysP, yeaE, srlD, yagE, clsC, wzxE, prlC, yidI, iceT, htpX, mrcB, ybiH, srlB, eco, fadE, dxr, cysJ, mdtA, glpF, otsA, clpB, mltC, yhhN, katE, cysA, cysI, yceJ, degP, argD, hslU, htpG, ytjC, dsbA, osmF, oxc, ybhG, hslV, galK, wecF, frc, wzzE, yqaE, argB, argI, mraY, wecB, yfcL, rffG, ygiM, wecD, ydjM, argF, nudK, yjeM, mdtC, mutY, yceI, yhgF, cysD, galT, cysU, bax, wzyE, rcsC, wecE, glk, galE, treF, otsB, mlc, opgD, dosC, setA, rffH, tilS, ptsG, wecC, nupC, ldtB, truA, waaZ, cvrA, bglX, degQ, yihF	*rimI, cueR, uspG, yeaR, isrC, purD, ydiH, insH25, yeaK, yjdI, yjiT, ybaM, alr, yhcE, purC, fumC, ybcJ, narL, ubiI, lpxH, alkB, ogrK, priA, fucU, yegW, rspR, dnaC, moeB, frdB, yidR, slmA, cyaA, aidB, hybA, citB, phnL, purL, mngB, amyA, mutH, uvrY, yffP, fliI, yfcU, nudE, uvrB, moaC, uvrC, yhgN, ppiC, tatD, ydeN, ybaL, insB1, moaE, ycgJ, proX, flgJ, yfcF, pnuC, intB, folD, pgaB, chrR, hybC, mntR, yfgO, insF1, fliS, ecpC, yghF, msyB, ggt, frmB, eutR, sgcE, yjtD, pepP, ybhL, ygaH, yhiI, rlmF, prkB, ybiX, ssuB, yfjU, mazG, pyrL, metJ, lipB, gadW, mnmA, yhaH, yeeA, phoB, frdC, yigL, rimK, yggN, flgK, proW, ampD, ybdH, moaD, yggL, tadA, yegP, ycgZ, yieE, artQ, ycbF*
(5)GSE1010 (GSE10158)	Cefsulodin 10 ug/ml 5min	*pinH, yfbN, ygfK, yddK, argW, rhaM, ygcB, motA, yehI, tnaB, tam, hycA, racC, paoA, ykfI, yehP, prpD, hiuH, yahL, ylbF, gcl, rhaA, fucO, gspG, abgA, rtcR, torD, hyfR, ymdB, yhiL, yfgH, rutA, uhpT, frvA, ypdF, essQ, elfD, ybgQ, yfeO, ydhT, phnM, aegA, yafD, clsC, abgT, mdtE, motB, yfdQ, ppdD, fbaB, holD, mhpB, ysgA, yhjG, pgl, arnB, yahB, narW, yjjQ, lsrA, lhgO, tauA, aspS, iadA, fiu, gsiD, eutQ, frlA, arpA, ssnA, atoA, phnK, potH, tauC, ybhP, purH, vsr, hyuA, yahE, yniA, ykfA, stfQ, yegV, elaB, yibF, mnaT, lsrC, helD, ada, yfbL, lhr, ebgC, fimD, ybcF, dfp, yjbE, rutG, hemF, puuR, yghQ, ygcO, hyfD, serU, allA, yjbF, murE, sxy, gsiC, ypfG, lgoT, rclB, yagB, frvR, entD, atpF, fhuE, torA, hsdM, ssuC, torS, eutH, ybdM, rrlG*	*rpoZ, ycgX, gnsB, ftnA, cohE, ytfK, yeeX, yeaJ, secG, mqsR, pmrD, appY, nhaA, apaG, sseB, ymcE, dicA, rnlA, insA8, emrE, intB, yihG, ynaJ, rcnB, glyU, yggC, yidB, yciB, yqjA, yoaB, rnc, ppiB, ycgH, prc, yebO, mzrA, ygjP, yrhA, nlpC, ydfD, rutR, gltT, yqiG, uspF, ampG, yoaC, rssA, ptsI, pabB, sapB, dtpC, slt, yidI, aroF, recB, yhaH, yeiP, cysS, yghU, mnmH, glgP*
	Cefsulodin 10 ug/ml 20min	*nuoK, ubiJ, hybB, phnN, yfcE, eutM, tfaD, sbcD, puuC, bcsB, rhsC, ddpX, ssuE, abgT, fimG, hisG, idnD, yfcU, yehM, ycbJ, agaC, astE, ycaC, yecS, rhaM, puuR, sfmD, yedK, hyfR, leuD, nirC, hycE, wzc, ydcT, cbtA, ydcU, ccmD, ydhT, torD, yedY, uidA, ydiF, yigM, insO, rhaA, frlR, cusB, bioF, pyrG, insA6, araJ, ycjY, lsrA, ydhW, yecC, agaD, bdcA, rhaB, fabG, yeeJ, eutH, ydcV, yhiD, lgoD, umpG, gltJ, idnO, yccJ, cysI, agaA, yedS*	*bamE, racR, yeiS, yqhA, yliE, ftsX, yzgL, yjgN, galU, ybfP, bamD, nudE, yghF, ybcY, yacG, gspB, glpG, yjiN, yehE, cspI, ybjS*
	Cefsulodin 10 ug/ml 40min	*galM, nuoG, nuoI, ompA, ibpB, pykF, psd, chaA, fdhE, alx, proB, oppD, ettA, proP, lptB, pepP, ppc, ygiF, gshB, glyA, mlaC, pgm, mlaA, ftsY, hemE, rob, glyS, yecD, surA, adiC, pnp, murE, ptsN, manA, ytjC, pcnB, ftsE, proQ, fecA, pfkB, insF2, yhjD, glnS, frc, wzzE, usg, yegW, sapC, fkpB, yfjV, selD, serC, rimI, eptC, lgt, basR, sapA, yheS, mdtO, oxyR, lptF, ubiH, glnD, hemY, acrB, rffH, tusC, ygaH, mnmA, metG, xerC, eutL, baeR, thiH, parC, sdaB, lplA, tmk, ynjA, tsaE, murA, ydhJ, mscM,*	*yhdU, ydaS, yfeD, ymgA, rbsD, yibB, pmrD, mcrA, ydfO, rhsE, ydcD, folB, mepS, ygaQ, cspH, csgE, kch, ygeF, lsrK, djlC, ycgH, elfA, intB, mioC, uidR, rem, yncJ, ycdT, ydfI, yabP, fabR, yhgA, ydiB, clpS, ygcB, tfaE, yecC, asr, ssuB, sieB, feaB, mltF, ydfZ, rnlA, rrrD, yhcO, ycaI, pyrE, yjiJ, djlA, ygcR, yciT, tktB, yqgC, chbF, allS, ycbL, ybdH, yghW, priC, yeeJ*

		insD8, alaC, leuS, selA, rsgA, spoT, rodZ, trmJ, mepA, yfcD, ygiQ, ispD, gpmM, mraY, hldD, sgrR, qseB, rnt, ybgL, menA, cysQ, srkA, hyfI, aroB, dsbB, ycfL, nagC, nemR, dacA, mukB, tatB, alr, rluE, wecD, tyrR, yfiP, nikR, ghxQ, secE, menB, pgpC, yfgM, yfbV, yggI, fabG, ftsX, yeiG, murG	
	Mecillinam 0.03 ug/ml 5min	*glyW, pinH, yjfY, gnsB, serW, glyY, ytfA, aspU, glpF, dicA, yfbN, yoaF, ygcB, panD, prlF, entB, torD, rutA, yadD, ybcL, glpB, ycgZ, yciN, dicF, yagN, ogrK, recE, yqiA, nagA, abgA, ysgA, frlA, yeeX, yhcO, gspG, yqeB, elaB, clcA, yjbQ, zapA, ypeC, glnG, paoA, mdtE, yfgH, mog, ydcJ, yihD, narG, rhaA, ecpB, mnaT, prpD, ybgJ, yehP, acnA, lsrA, abgT, iraD, ypjA, yahN, yfeS, ypfG, iadA, ybcF, fucR, rzpR, yhdP, cusB, yafD, yibA, ylbE, yeaM, ydhT, phnH, rhaS, rrlG, yddH*	*gcvR, ynfC, ykgA, lpxT, yedZ, yjfL, emrE, rrrQ, yedV, cedA, yfbU, ydiP, sseB, yibG, slt, rluC, rnc, ypdB, yfjL, yihY, pgaA, ygiC, yfdE, ghrA, iscS, yeaY, ygjJ, yejK, yjhV, pyrE, ybjE, yggU, tdh, sapB, intD, ldtB, rnr, yhiD, pheP, yfbV, yrbL, yeaE, yhbV, secE, ydaY, yqgA, holA, mdtF, zinT, yhjK, ydfD, uup, yphF, rmf, ynfG, ligA, gsk, gspD, eutH, yfjS, yghB, yggT, srlR, pabB, dcm, yniC, iscA, ydgJ, yoaC, hscB, yijD, dtpC, pbpC, eptB, malZ, yfjP, bamB, yeeL*
	Mecillinam 0.03 ug/ml 20min	*cydA, nuoK, fecB, glyQ, fecC, nuoM, cyoA, fecA, sspA, rne, mreB, ubiJ, sspB, leuS, pepB, ybjJ, hypB, mreC, pfo, secD, glnS, ftsE, metG, opgH, accD, napF, maeA, lptD, hybB, pyrG, cydD, ftsY, valS, ilvI, yciB, ycjO, acrA, cheA, dfp, fmt, mqo, ubiE, parC, thiL, yfjZ, arfB, iadA, murA, psd, fnr, abgT, yheT, rhaM, phnN, ydcU, hycE, ypeC, cusB, ugpA, ybjD, lpxL, proY, sfmD, ispD, ydcT, fimG, tdcD, rho, sapB, agaD, yhiD, dmlA, idnD, wecA, priA, ybjL, yehM, trmL, sbcD, purM, rsgA, ccmE, agaA, gltJ, yecM, fadA, yedK, leuD, ybjS, ynfG, cynX, mreD, recQ, yecC, btuF*	*zinT, ybbD, yibA, yqjC, mscL, rcsD, yhjR, ydaS, yfbP, rraA, yjgX, rhsA, bhsA, yggX, dkgB, phoE, gspB, yfjH, yfdK, yliF, yjhD, yfaD, sohB, citA, phr, yehE, yahI, mug, emrY, yehY, yeeJ, ycjV, ycdT, yqiG, asr, csgA, yeaN*
	Mecillinam 0.03 ug/mll 40min	*ibpB, ygiM, hupA, ygdR, ygeN, pykF, treC, rseB, hybF, hisM, fabB, proP, cysQ, yfcO, ravA, yhfY, nikR, idnO, xdhB, phnP, ulaD, kdgK, lexA, ynjF, yjfK, ppiD, ybeL*	*mscS, araD, tdk, trmI, emrE, hrpB, yceO, phoE, nlpI, sieB, araC, allS, ybcL, gcl, mrcB, mdtB, yecH, yccF, mscK, rsmA, xylH, abgR, yeeP, gnd, gspK, fepC, pyrE, yjjI, rutD, ygbK, purT, psiF, yccJ, proW, rpsS, yhaI, yjdC, ghxQ, arfB, puuR, kdgT, ybhJ, moaD, yhbJ, lolD, proV, rimM, purN, mlaE*
(5) GSE1010 (GSE10159)	Cefsulodin 60 ug/ml (MIC)10min	*rprA, osmB, murQ, yqiJ, murR, yabP, ydjH, ybcM, arpA, yrhA, cysH, yiaL, rclB, alaC, ygdR, yidX, ytfI, ydjO, rhtB, ykgA, yedN, mazF, eco, yjeN, yeaM, ybeM, glpD, alkA, yedS, yadC, ypfG, yneL, yebY, phoQ, uspA, ybbD, ydcX, yceI, nagA, gcvR, ybcO, adeQ, feaB, gapC, yjiG, yibQ, yjeH, evgA, fecI, ygeN, yfiB, cybB, crl, ydiH, mutY, ppx, agaB, yheO, ybjN, yiaO, yddK, ibaG, map, rplE*	*sfmC, ybgS, yjbG, ddpD, araB, astE, hyaF, yehL, ileS, stfE, eutN, tfaD, nrfE, glmU, puuB, hyfC, yehX, glsB, purF, dacA, degP, proA, ygfK, mnmG, cysN, yfjR, truB, ybiI, ydjJ, skp, ydhU, dcd, gfcA, lptD, rrsG*
	Mecillinam 0.3	*agaB, ydaT, gnsB, micA, ydjO, ygeN, uacT, flgI, yedV, eaeH, yncI, yadV, yhdY, elaD, ydiM, intE, ynbB, ybfC, rydB, stfE, atoB, ydaC, hofM, gspF, ymdA, yihQ, tdcG, pbl, ykgM, leuD,*	*accB, csrC, atpF, atpB, rplM, glyS, gpmA, rpmB, pnp, glf, groL, yebC, mltD, ruvC, crp, treB, rpmE, tatC, speA, hpt, rpsU, iclR, acpP, ygfZ, atpA, hflX, rapA, ppiB, aceE, slyB, iscA, wbbI, nsrR,*

	ug/ml(MIC) 60 min	*entD, lsrB, ygbA, yeeD, ompL, yedS, bluF, yecC, rhsC, tmpR, rhsJ, yciQ, cbrA, gspE, ais, bhsA, rspB, thrL, mngA, rem, hyfF, mdtH, essD, yifN, cadA, rzpQ, yeaX, eutD, abgA, yqfE, yddM, ypjC, ynjC, hyfC, gspI, bglB, yiaV, rhsE, yehH, gmd, bglF, rzpD, eutR, flhC, dmsC, ybgD, ecpR, livH, gspO, gudP, micC, yffM, fadE, feaB, wcaH, ycjV, ymfL, ynaE, ycfJ, flgL, ygeY, rtcA, yfcU, ykfF, fliQ, yncG, emrE, uvrA, ygcW, ybdL, lyxK, yegE, yhiS, ydeJ, ygjK, yeaW, yfdE, cheZ, dsrB, cusF, sokC, kdgT, cheA, ybdJ, fsaB, kdpD, argH, yqiJ, yddK, csgG, yghA, hydN, yhhY, rdlD, yhbU, ygaY, yaiO, mdtL, ymfM, dosC, ydcT, kbaZ, yjhF, gltJ, hokB, yghO, ycjO, ypjK, yhjJ, gabT, yfcO, yhbX, mhpB, fes, bcsB, ygeO, arpB, stfP, tdcB, psiF, yfjM, pagP, yjdJ, sapA, ydcA, rpiB, ybbD, norR, acrS, ybcV, yijF, fliG, ygfM, yfdY, pflC, bcsC, yfjD, ybjI, yedN, ydcF, ascF, yafZ, lysC, yabP, hypF, yibG, xdhB, yihU, yehQ, ybhP, potF, wzyE, yfdF, ygeI, arpA, ybfD, ytfT, yehC, tfaX, ybbC, artM, yqeB, yedE, napG, yahM, yeaQ, alkA, ygeQ, fucI, citD, insF1, rsxD, yfaS, araF, yjcS, ydjZ, yieP, ydjX, hisM, katE, mdtF, yebF, betB, loiP, rsmB, sufB, araA, fucO, wcaG, yiaT, tdcF, ydaG, yidG, cysG, atoA, ycjP, hokE, yffL, idnT, araH, lsrA, ymgD, zinT, yaiV, gspK, yjhB, yiiQ, torZ, yjiR, yraH, rhaD, thiH, ycjF, ubiX, tfaS, dgoK, csgF, betT, yecR, ymgG*	*rplJ, dsbA, yobF, hslU, flgE, trxA, iscU, secG, mscS, gcvT, coaE, elbB, tusB, folE, uvrY, tdk, gatC, rraB, ratA, fabZ, aspA, ygiB, adk, mprA, rpmI, csrB, gmhA, hscB, serB, pheS, ettA, yafK, ydhQ, yeeX, lptA, fabB, mreB, oppC, zapB, apt, slyD, fimA, tnaA, metK, alaS, ydgA, yceI, hisQ, fur, nuoK, glnB, yeeN, dapA, lysU, ryeA, yqfB, fldA, dacA, gtrB, rhlE, artI, yibL, waaR, rpoB, yfeY, fdx, proS, rpoZ, waaC, stpA, lepA, prs, pgi, cvpA, gph, mlaF, mreD, nuoF, ribE, flgD, glpD, dxs, yaiL, ileS, yibN, grxD, mazE, tesA, ftsP, ompT, iscS, yhbY, lon, fetA, pyrH, rsmH, pcm, gyrA, yciN, yfcE, yggT, rnb, elaB, mnmE, yeaD, tyrB, radA, der, ribC, fliY, ygdG, rhlB, yihI, pal, phoP, relA, cyoE, plsC, psrD, djlA, spf, fabR, cysK, tolQ, ytfP, mqsA, ftsW, waaQ, ebgR, cysS, ydiH, cpxR, yrdB, fis, tpx, wbbH, minE, rlpA, recJ, lpp, iscX, mscL, fdoG, pitA, yciI, glnS, ratB, ldtD, yidR, ygiC, wzzB, clpB, dgkA, ybeY, rpsO, miaA, glgB, fecC, allR, roxA, polA, prlF, wzzE, rna, rpoE, lpxA, yqhA, rplU, yjfN, yobA, dicA, umpH, mog, psd, mutS, ycbL, ypjD, hisF, gltS, yagF, artP, yjiX, chaA, yqiA, ibaG, yigA, yciB, yjgA, manZ, ftsZ, murI, ispB, cspC, metG, yggU, ygbL, sseB, ccmC, bglA, ubiG, nmpC, proX, dnaA, pck, dinJ, glmM, holE, sohB, fliF, bcr, epd, ndh, yqhD, mgtA, clpS, mukF, fbaA*

Table S6: Combined list of upregulated in all samples of each stress

Stressor	Upregulated genes
**Heat**	*osmB, htpG, mqsA, rpoH, bolA, relE, alaE, ybfA, yhaV, ibpB, ibpA, yhhA, ycjF, fxsA, yncJ, ygaC, bhsA, ycjX, clpB, pspB, pspA, pspD, zntR, dnaK, marA, phoB, pgaD, pspC, ybeL, ybbN, yfdY, ychH, ydeP, uspB, wrbA, hchA, uspD, yqhC, relB, chaB, higA, narP, dacC, mdtK, raiA, pepT, yjjJ, metR, ycfJ, ymgA, ymgD, degP, mgtA, mutM, yjbJ, hslV, ogrK, bssS, yaiY, cnu, hslJ, pspG, ybeD, grpE, htpX, yhcN, ldhA, iraD, nrdD, soxR, yafE, yhfY, yjfY, yibA, prlC, spy, yafD, hslR, hslO, phoR, sdaA, yciM, dnaJ, yhdN, yffR, uspG, psiE, yiiX, appY, mqsR, clpA, ytfK, glgS, araC, mlc, cpxP, yqjE, ygiW, uxuA, mngR, lon, rpoD, uxuB, ybdL, prlF, yneL, fucU, smrA, slyX, yhjY, ybeZ, yfdT, yjfN, grcA, yecH, bssR, ymgE, clpS, yibT, sugE, torR, yffO, ynfK, yeaJ, ybdH, gltS, yphH, metB, uspF, ilvY, yiiS, nfuA, mmuP, chpS, metJ, creA, malX, potE, metN, mmuM, csgA, ycgZ, dgcZ, ariR, ilvM, ydeS, ytfP, nrdH, yebE, hspQ, ydeT, ydeR, ygdI, yccJ, otsB, ymgC, tqsA, ivy, yjdJ, rhsJ, yfdK, ygdR, yqjI, nrdI, gltP, ybgS, corA, yliE, tfaP, argF, yahA, ldtC, rlmE, intF, yciS, xapR, argC, bdcA, groL, afuB, emrD, yddM, yjcB, pgpC, gadX, yhfG, tomB, yodD, mgrB, yqfA, ybcW, fruA, artJ, sbmC, argI, dmsC, yphD, yhhQ, gntP, csgB, hokD, arsR, cspD, ycbJ, norR, rpoS, yodC, rcnB, rem, ybjX, groS, yadM, clpP, udp, yffM, ydeQ, gntT, frdD, higB, yjdI, yadL, rpiB, yffQ, argB, ghoS, nirB, hcr, casE, sufA, rof, ygbF, yhfX, torY, gntK, wecH, yneM, ybjQ, torI, pmrD, yeaR, nirD, gspB, dmsA, rusA, mcaS, phoP, argA, eamB, ccp, flxA, yqjK, yhfW, yicH, uxaC, lacY, fruB, ytfH, yffN, pgaC, yhfZ, ydfZ, metF, yrdA, yqjA, ypdI, melB, malI, bsmA, yoaG, hdhA, dadX, aceK, miaA, ftsH, csiR, fruK, gcvB, ompW, arfA, ydhQ, holC, frmR, creB, frmA, ykgF, chpB, yffP, ysaB, ftnB, yadK, matP, yfcZ, yhbS, yjfM, zupT, frdA, yafS, yoeB, dmsB, rhaT, ycgI, mrcA, ydaC, yibG, ypfG, yabP, ynaE, yehH, yhbO, yfeS, marB, pfkB, hslU, yqaE, bluF, fbaB, leuE, soxS, stpA, ymgG, yidB, yieP, yjaA, sixA, ygeN, ygeQ, elaB, otsA, ydeJ, yiaG, ykiA, cysQ, yfhL, fecI, gadW, nrdF, arpA, ybfG, ycaC, rmf, treF, tusB, eco, ilvG, fimG, ybbC, bglB, recN, yojI, yjhE, yfbN, tfaS, frc, cusB, ydbA, yedV, ymfD, yifE, gadB, gadA, ypeC, yhjD, yifK, yheO, atpC, ycbK, polB, yhcO, nanC, betI, ydeI, ynaI, ykgJ, yahN, kbl, trxC, yjeJ, yfcE, yhhH, srlD, fucO, symE, yeaM, ybfD, ybjH, ydfC, cbrA, grxA, eutR, ycgB, yeaG, nrdE, sra, yihF, bglF, recF, cadA, essQ, rlmB, dsdC, yidX, flgL, yedR, cdd, cbpA, cbpM, ygbA, yjgX, ychE, csiE, yffL, qorA, fsaB, pyrF, rclB, yghO, creD, yjhF, osmY, yehX, puuA, yegP, ydfK, yncD, appA, yadD, umuD, yffB, tdcG, dusC, yaiO, sgbE, ytfI, ypjA, sxy, eutQ, yfjD, yhdW, ryhB, waaZ, eutD, yqeB, ydjF, argH, srlA, ynbE, ycgH, yehQ, wzzB, elfA, yhfL, entF, torZ, araF, nfrB, kdgT, yagN, acnA, yqjC, ybaY, msyB, amyA, wcaD, yfcG, thiC, yidL, yidQ, csgF, yfdV, fucR, yhcF, tfaD, crl, fimD, yfbV, cfa, can, ygaU, gfcE, ykgH, yiiE, kdpC, ynbB, rhsA, ydiP, waaP, xylG, yhbU, yagK, mscS, nanM, ydfF, yiaB, pgrR, atoB, cho, waaQ, hlyE, ydjH, ycdU, ygiZ, xylR, yjeH, ais, tfaE, ompF, yqjH, fimE, yagM, yccX, yfcO, htrE, aegA, agaC, ycgM, ydhZ, clpX, setA, ybeX, zapB, ecpR, lysC, yfaZ, ybgJ, galM, frvR, ybeY, ydeM, ryjA, oxc, yhdY, ihfA, cohE, cbrC, yfjU, mnmE, bioF, yoaF, yffS, treC, fabB, hemF, yagH, ydcA, wzxE, nlpE, insF1, ompL, cysE, ybjP, blc, tehA, ybaV, yjaB, yohJ, xylH, mntH, nsrR, yacL, yfbP, yicJ, yfaH, frvB, waaH, dadA, yjiJ, phoE, garD, yhiJ, glvC, gltA, glgA, elaA, ilvC, alsA, yiiD, ytfJ, ftsA, rclR, metA, yqiK, sgcQ, ydbL, gudD, ydgD, norV, yohK, feoA, ypfM, copA, bfd, ybjM, dinJ, ykgE, mokC, yrhC, ybdF, rimJ, yhiI, amiC, emrK, ygeF, yqeK, yegJ, yliF, yhhZ, yigF, apbE, yqiH, glgP, ydfR, narJ, fdnI, sad, cadB, yhjX, ypjD, gfcC, ydjG, araB, ydfD, xapB, yiaA, pppA, intK, fucK, yfcQ, mtfA, aldA, maeB, yghT, sfmH, yfaQ, yehB, aes, ushA, yhaI, yfcP, ydaY, frwB, yggF, borD, tag, ylcG, uidC, yaiP, aaeX, ralR, preT, yjgN, fucP, ydcC, yfdL, fimF, kilR, ydiQ, yfdS, yjfL, yqeH, xylF, caiE, ydeO, yijE, ydjE, frwD, yjgL, yqiI, allB, yghF, hflC, ygiV, yhdZ, nudL, yeeW, ygaM, nlpD, ydbC, tktB, ghrA, mdh, yehW, btuB, talA, waaO, rpsO, gnsA, ftsW, yeaD, sibB, katG, pnuC, dkgB, xylB, yafU, yjfJ, yfeA, valV, creC, ldrC, iraP, yhcA, valW, waaA, cdaR, bglG, ybiC, pphA, glgB, treB, aphA, yqjF, ftsI, insG, yjiV, emrY, ybbA, narX, yraK, murC, yfgI, narI, rfbA, aceB, deoB, aceA, lldD, napB, wbbL, bfr, ddlB, tamB, ycdT, yfeN, insL1, grxB, ulaG, psuG, acuI, mtn, waaS, wbbH, fepE, rutB, quuD, add, ybcH, idnR, metL, yfhH, ybhM, waaB, yneO, ydbD, yhjC, dsdA, yphB, pphB, ftsQ, mtlD, ccmA, glpQ, ykgG, ydaU, ydfJ, waaR, rhsD, yjhR, yciH, insH11, allR, wzxB, yfbK, pspE, wbbI, yqiG, yebW, yjbI, yphF, glf, yggP, rfbC, waaU, yihO, ridA, eutT, yjiT, sieB, ydaE, yeeL, yefM, yihV, rbsD, yhaC, ydaQ, ispH, csgD, dcp, idnK, ycfZ, yehD, kdgR, tdcC, yibS, mdaB, fimC, hicB, nlpC, dgt, ycgJ, yhcE, fimA, bepA, rfbB, yphE, caiF, hokC, ydfX, insL2, yfdX, pptA, ppsR, yfcV, yjhI, cobU, yhcC, yhjB, hcaD, ynaK, mrr, secA, insL3, ompT, rdgB, ygbT, ybcN, yjcF, hicA, trkG, ycaK, setC, yjhG, hscC, pfkA, djlC, yehA, insH8, ysaA, glgX, ygeV, glgC, yhbT, hflX, yaiX, yihN, ftnA, yjdP, aqpZ, mglC, ydhV, yccE, mobA, ftsZ, plsB, rhsH, ydjJ, nudF, hflK, bdcR, gldA, coaD, intB, yfbU, yfiE, blr, uhpA, cobS, ydhY, yhaH, ttdT, lrhA, yciZ, yciU, rpmE, rbsC, yjhC, yoeG, fucA, lgoR, yibW, yjbT, insB1, csgE, fdhF, yaiS*

**Cold**	*puuE, arpA, cspF, osmY, sra, rnr, ybfG, mdtJ, puuA, gadW, kptA, puuP, ykfB, glgP, ansB, yihF, crfC, pgpB, bglF, ychQ, yehX, yibF, yqfA, shiA, yzgL, yecA, recF, ynaJ, ybbC, pnp, srkA, pepQ, ygaY, proV, rpoE, ebgC, alaE, ytfK, aroG, yieP, nrdE, lexA, yaeH, yddM, feaB, waaH, yadE, yeeO, ycjG, yoeF, mlaF, nrdF, yrbL, ydcZ, gcd, obgE, gsiA, csrD, cspI, ydfK, ynaE, cspB, pinQ, lpxP, cspG, pinR, ydfR, bluF, dinD, eptB, dgcZ, bluR, sugE, yjaA, nepI, proP, yejG, yjcB, yedA, yebS, rmuC, yieE, yegP, rpsC, ynfN, nhaR, rplA, safA, rplV, ydeJ, trmD, cspH, yfbP, mazG, yeaG, thiC, ycaC, glmS, rpoB, hemG, yiaG, pgi, rlmM, phoH, panM, hokA, waaO, bdm, gadE, aceE, dctR, ppiC, cpxP, yfjP, amyA, glpK, ycgZ, tdcD, ybaY, ascB, ymgA, yidL, opgE, rpsO, melR, ygaM, ymgC, gnsA, glpD, ycgX, proW, leuS, yebE, ycgB, gspD, ydbC, zntR, glsA, bssS, rhlE, ygcB, patA, insJ, fbaB, ftsW, trpT, gpp, nsrR, yeaH, puuR, rzpQ, adhP, puuB, yeaQ, fusA, astE, yeaD, gfcB, trpC, ybgA, sibB, puuD, cadC, acnB, ydiZ, gadX, purH, degP, rrrQ, tktB, rydB, infB, blc, sfmD, garD, yceI, trkA, ydaM, prmC, fxsA, elaB, rmf, mtr, setB, phr, katG, gfcA, hofM, pnuC, torY, yidK, gadB, yifK, ymcE, malS, rplF, yhcD, serT, yaiA, iraM, hisG, dinG, trpA, nlpA, dtpA, ubiB, ydcJ, yeaE, fliI, essQ, katE, polA, aslB, rlmB, glnD, aroM, yniA, yhfK, mqo, hemD, yecT, yehW, yijO, betT, htpG, yaaY, yeeY, insK, fiu, bamA, yfhR, deaD, panF, mdtK, proM, yehR, yhiJ, ybiX, dkgB, poxB, brnQ, maa, acnA, xylB, yebO, ykiA, yhfG, degS, yceJ, kefC, thrV, zraR, wcaD, mdtI, nrdH, nfi, ggt, yneF, galS, corA, tpr, astA, yafU, tfaR, clsC, rrrD, sbcC, fepG, secD, nadA, ppdD, yjfJ, narZ, ppx, ypfG, yfcG, fliQ, uvrA, otsA, yfeA, yejE, sslE, serS, metF, bglJ, glvC, rttR, gltP, yidQ, pheU, raiA, gabD, ydhC, argU, fliR, btuB, iclR, yfjW, pheV, fepD, dsdC, flc, uspF, yadG, gltA, lpoA, nrdI, yodC, yabI, lldR, ldrB, spf, gspC, arnT, puuC, fliH, yicJ, yaiT, ybgS, ypeC, ftsE, fkpA, ypdA, ytfL, gmr, glgA, recQ, yciN, waaC, rsxG, gadC, fsr, fau, ymdB, mgrB, yghB, hisL, mtlR, yfaH, dosC, fadM, allS, gspK, yhfU, flhA, dld, ybgD, gdhA, mzrA, cspE, metI, tomB, rseC, elaA, yeeX, zraS, nlpI, yaiY, wcaE, rpoS, kefB, ldtC, yedQ, fepC, fic, yidH, valV, nadB, deoC, ppdA, ydaG, ykfC, alx, rsd, dcuA, yhcN, bcsE, ilvA, greA, ykgA, ydcI, essD, potF, rlmE, dcuR, proY, wcaF, chaB, yfiH, flgK, rcsA, hsdR, ydiE, proX, creC, yobA, tas, casB, hofB, entS, ldrC, iraP, rpmB, lgoT, astC, yhgE, ldrA, lhgO, osmB, tatC, sdhC, ybfP, yhcA, csgF, ybcJ, nudK, mnmH, valW, yafT, ampE, aaeB, yohD, tehA, ilvC, yeaL, alsA, yhjG, hcaF, yagJ, gabT, glpX, yajG, ybaN, yjiJ, yhdU, ygbE, yjfK, yccA, yehY, prfH, accC, infA, yidX, ymjA, opgC, sulA, fliO, dcuS, ydhP, yqeJ, yrdA, omrB, osmF, ybaA, yfcU, ydhI, metQ, hisD, waaA, yeiB, yiiD, curA, chrR, yaiE, frvB, fdoG, cnu, phoA, ydfZ, yohO, zur, yjjQ, rimL, sdiA, rcnR, rsxA, nanT, ytfT, gltW, dbpA, rcnB, ycbF, gltV, yeiE, yqjA, slp, ybhS, cdaR, truB, fumB, dmsD, yiaJ, mdh, ybfB, sokB, cspA, yecJ, leuE, sufE, ugpB, yghU, eutG, ygiD, yfeD, ytfJ, yfdV, yedI, wcaB, lyxK, trpB, yccM, yibG, gltT, ydeE, yqjI, ubiX, sibA, alkA, sufA, bglG, ebgA, ycaL, lgoD, fecR, ulaE, yfeC, wzyE, ycfJ, ybiC, galP, acrF, tfaQ, mtgA, nlpD, acs, nrfA, yrhA, yccJ, yheO, yfiR, flhE, gltD, rnd, rem, gltU, hofC, wcaA, ybgQ, kefG, yidA, sucD, ydiU, yhfX, yeiW, bcsG, yrdB, yqjC, oxyR, ndh, lnt, prlC, yacL, yigM, yfiM, glxK, ycjX, yegE, yidD, yegS, ydgI, atpC, pphA, pck, flgL, ftsA, tqsA, mpaA, ssrS, dadA, ypdI, glgB, fliP, mdtG, ptrB, ydgK, rclR, yhbE, yhhW, racR, yjbJ, psiE, tsaE, ygfF, ydjN, rssA, tsgA, tusE, yliE, bolA, yfjV, allD, yahO, cysN, ftsX, aspT, yaiI, treB, rbfA, uxaB, murE, msyB, pepB, hyfR, argT, rlmG, ygbF, yeiG, tehB, aspV, ecnB, adeP, aphA, rprA, dppB, zntA, serA, cbdB, fdoI, rdlD, ilvM, melB, trmN, aspU, yfaY, dnaE, uhpC, fadR, uidA, ybdJ, yihM, fdhE, holB, yhaV, yjcZ, dps, cpdB, ryfA, pepD, yqjF, rseB, fliG, rseA, envC, rhtC, yihG, ftsI, yniD, acpH, gntR, yjbM, sibD, tusB, dinF, ybgO, insG, gltB, yjtD, wrbA, fucR, yjiV, ileU, mdtO, ybaV, yhcF, ybdK, fdoH, ybdZ, gadA, yhcG, glmM, mntH, yfgJ, rpoH, yadI, metA, ydiH, rimP, npr, ychO, galR, citT, cadA, yqjE, yciQ, argZ, yfeS, emrY, opgB, ampD, hsrA, pgm, rtn, queE, smrB, rarD, yjbD, ileV, yajR, ykfJ, wzxC, ydfV, glcD, yiiG, ileT, yifN, ydhK, talA, crr, pqiA, pspG, otsB, livH, mlaE, yodD, argY, alaC, ahr, ycbU, sdhA, mglA, ebgR, yebZ, ubiA, ilvY, malI, yaiW, argV, ygeX, malZ, yebQ, yhaM, ghrA, rdlB, bisC, ybbA, dsbB, arnA, dacC, ygdI, rna, ybeD, yqjD, mnmG, yeiH, yaaX, glk, yceK, araJ, pgpC, rof, yhjK, gspJ, dcrB, acrZ, ydfU, bcsF, yadH, viaA, ycbK, bamD, yjgH, smpB, ybhD, yggI, rnk, cof, rnlB, ygaC, lsrG, argQ, yqiK, sgcQ, yfgO, yeeZ, ydjM, hcaR, sdhD, yhdP, ydbL, yoaC, yjcC, msrA, insZ, polB, yheS, hisC, pgsA, fadD, yhfS, yhcO, rcnA, tfaD, gltL, zapC, yhjH, yhjJ, narX, fliK, barA, gntK, yidC, rybB, crl, priA, fimD, ydeP, higA, bcr, yehE, endA, xylE, yebG, queG, yraK, fepB, wecH, yjfI, chbB, gspO, yggN, slyA, adiA, ycdZ, yfbV, ydjZ, ylaB, gudD, yhbO, yjfF, ycjF, chbC, bsmA, yjeT, tsr, thrA, yafK, yjeO, ysgA, mnaT, sthA, tldD, cycA, yfeK, btuE, yiiX, acrA, yfcF, yhjD, yqhA, ppdB, potG, phoE, ssb, marC, asmA, glpG, rhmR, ychH, yddB, sbmA, ydiV, murC, mscM, pepT, yfgI, hisB, yrbG, caiT, yejH, gsiD, allE, treF, pgpA*
**Anticiotics**	*osmB, ibpB, ycfJ, ycgZ, dgcZ, iraM, ymgA, ymgD, degP, ariR, ypfG, ibpA, yigI, yeeD, cysI, holD, rimI, ygiM, ydhJ, puuC, yabP, yciG, nhaA, fadE, yahM, yhhA, ilvM, ycjF, htpG, fxsA, mgtA, mutM, yjbJ, cspF, srkA, ynaE, tpr, ubiX, ydjM, fadD, yhjJ, yehH, yhbO, yfeS, yncJ, hslV, ogrK, mdtE, cysH, ldtB, ydeS, ytfP, rnpA, gpt, yeeE, cysP, cysJ, emrE, ybfE, lipB, mutY, entD, yncI, yagI, fadB, gfcA, yebO, ydaG, marB, pfkB, hslU, yhiD, yciF, leuD, betB, yciE, paoA, thrL, yghA, fadA, nrdH, bssS, yqaE, ygaC, yaiY, cnu, bluF, yebE, fbaB, leuE, bhsA, hslJ, hspQ, yqiJ, soxS, ycjX, stpA, mdtJ, ydeT, pspG, ybeD, ycgX, mdtI, ymgG, yidB, lexA, ydeR, yieP, yjaA, ygdI, yccJ, otsB, ymgC, tqsA, yhbE, maa, rttR, yhdU, ygbE, rsxA, ydgK, ydjN, trmN, yggI, hcaR, yidC, sixA, ygeN, ygeQ, elaB, otsA, ydeJ, yiaG, ykiA, clpB, pspB, pspA, pspD, grpE, htpX, yceA, cysD, mdfA, yedN, yciA, cspI, ivy, yjdJ, rhsJ, yfdK, ygdR, ybgC, mioC, mntP, ymfM, efeU, cysU, yciW, yebF, yiaD, elyC, gnsB, thiH, pabA, folA, ybjG, queD, yjiR, ymfL, uhpT, pth, ibaG, rfaH, ytjC, mltA, wcaG, fecA, atpI, mreC,*

	glpE, menA, bdm, nhaR, puuR, hisG, astC, rprA, ysgA, cysQ, yfhL, marR, osmC, gmd, yjhQ, ybhP, nrdB, glnG, ykgM, metG, ubiJ, yjhP, pstS, gcl, yfcD, ymfE, yfgH, chaA, glyA, glpF, allA, mdtF, insO, yegD, ecnA, murA, dfp, renD, rhlB, pagP, omrA, ydcX, hldD, eutL, rsmJ, dsrB, yqjI, yhcN, nrdI, mqsA, ldhA, iraD, gltP, ybgS, slp, ydiE, fecI, zntR, corA, yliE, gadW, nrdF, arpA, ybfG, ycaC, rmf, treF, tusB, fes, nrdD, soxR, yafE, proP, yedK, yfcI, yddH, mhpB, eco, ilvG, rpoH, yhfY, bcr, yjfY, yibA, prlC, yfcU, yifN, fimG, dinD, cspG, tfaP, argF, yahA, ybbC, ldtC, rlmE, ydiH, potF, wcaA, glmS, gpp, sfmD, aroM, yecT, clsC, uvrA, fliR, recQ, dosC, wcaE, greA, essD, proY, infA, ymjA, opgC, ydhI, phoA, alkA, wzyE, yegS, yeiH, yebG, yafK, glpG, mscM, bglB, recN, yojI, yjhE, yfbN, tfaS, frc, cusB, ydbA, yedV, ymfD, yifE, gadB, gadA, ypeC, yhjD, yifK, yheO, atpC, ycbK, polB, yhcO, yedY, exuT, aidB, dnaK, marA, phoB, pgaD, pspC, ybeL, ybbN, xseA, thiB, fhuE, spy, yafD, hslR, hslO, phoR, sdaA, yciM, dnaJ, yhdN, yffR, pfo, yidZ, cysA, gspG, murJ, ygfK, ydhT, zinT, yghG, tsaA, rep, rhsC, trmA, nudE, acpT, hybB, yibF, pepQ, feaB, mlaF, cspB, intF, yciS, xapR, argC, bdcA, groL, afuB, fis, ybhB, rstA, yhiM, ybiV, agaB, ymfR, jayE, cysW, manA, stfP, lpxT, rluC, ybjE, asnC, yehP, hyi, yjgM, yneE, rph, agaD, gtrA, tolQ, cutC, rsxB, ydcF, rhaM, tsaB, yibQ, yidI, fhuD, nagA, epmA, atoA, ybjO, ybeQ, fecC, mreD, wcaH, yddK, yedJ, rnhB, ybjJ, prpD, entB, yedS, mepS, nuoK, secG, nikR, idnO, idnD, ilvN, tmpR, yadC, mhpR, yeaY, yjhD, yehM, yfaE, ydgC, ycjV, ychF, ratB, hdeD, yecM, rhaS, gadC, phoH, gadE, ydiZ, katE, poxB, leuS, rcsA, lhgO, gabT, curA, ahr, cycA, cspH, ppiC, glpK, glpD, ygcB, insJ, gfcB, puuD, yceI, yaiA, ubiB, yniA, narZ, fliQ, ftsE, yciN, fadM, yhjG, omrB, yigM, ftsX, argT, fdhE, rseB, yoaC, mnaT, nanC, betI, ydeI, ynaI, ykgJ, yahN, kbl, trxC, yjeJ, yfcE, yhhH, srlD, fucO, symE, yeaM, ybfD, ybjH, ydfC, cbrA, hdeB, hdeA, rho, rpmH, iap, mreB, uvrB, dxr, wza, wzc, ybhN, guaC, ugpA, atpF, dsbA, ymfJ, pinH, murR, pykF, murQ, ydjO, intE, ddpX, insQ, lptD, ymfQ, nrdA, rutA, stfE, torD, psd, ftsY, yeeA, phnN, valS, glnS, yibB, micF, frlR, abgA, rhaA, pyrG, phnM, abgT, mliC, sbcD, hycE, ydcU, ssuE, frlA, yjbE, glmU, iadA, parC, ypdF, yecC, nagC, recE, yjbQ, uspA, ybcM, tauA, slmA, tusC, agaA, ybfC, ydcT, bcsB, ybcF, ispD, mlaC, psiF, rsgA, dut, sdaB, gltJ, adeQ, ybbD, ubiE, rrlG, lsrA, ygfM, sapA, fecB, yjbG, rstB, leuA, hemY, nupG, yeiI, wzzE, mdtL, yecD, tauC, yhaO, glcC, yihD, yigZ, secB, trxA, loiP, leuC, tdh, kdpD, yigL, hemX, yfeY, ppiA, rffH, mraY, yihA, dacA, wecD, tatB, yicC, yggE, yidR, yraH, truA, gcvA, bamE, bax, yhgF, fabA, yiaU, ygeI, yidG, yfcL, yfdY, bolA, emrD, ychH, uspG, yddM, yjcB, psiE, ytfL, fepD, pgpC, gadX, yhfG, tomB, yodD, mgrB, gspO, ndh, zntA, sulA, ybhS, grxA, eutR, ycgB, yeaG, ydeP, nrdE, yqfA, yiiX, sra, yihF, bglF, recF, cadA, essQ, rlmB, dsdC, yidX, flgL, uspB, appY, mqsR, clpA, ascF, yrbL, yaeH, ybcW, fruA, artJ, sbmC, argI, adiC, garP, asnA, ybhL, nemR, nrdG, ydcY, dgoK, dps, fic, yeaH, yjcZ, yedR, cdd, cbpA, cbpM, ygbA, yjgX, ychE, csiE, yffL, qorA, fsaB, ytfK, wrbA, entS, gspK, rlmG, mdtO, fepB, pyrF, ykfB, dmsC, yphD, glgS, araC, mlc, cpxP, yqjE, wzxC, opgE, rseC, lgoD, mlaE, rclB, yghO, creD, yjhF, ygaY, ebgC, nepI, yhhQ, gntP, csgB, hokD, arsR, cspD, ycbJ, norR, ydaC, osmY, yehX, puuA, yegP, ydfK, rpoS, yodC, rcnB, rem, yibG, mqo, dctR, proX, betT, wcaF, acs, tdcD, rzpQ, puuB, phr, hofM, fiu, fepG, ppdD, sslE, ymdB, fepC, alx, ykgA, lgoT, yjfK, sdiA, nanT, ytfT, dbpA, lyxK, fecR, yrhA, rnd, ybgQ, ydiU, lnt, ydgI, tusE, cysN, hyfR, adeP, rdlD, yihG, dinF, hsrA, pgm, alaC, araJ, msrA, insZ, rybB, ycdZ, ydiV, yncD, appA, yadD, umuD, yffB, tdcG, dusC, yaiO, sgbE, ytfI, ypjA, sxy, eutQ, yfjD, yhdW, ryhB, waaZ, eutD, yqeB, ydjF, argH, srlA, ynbE, ycgH, yehQ, wzzB, elfA, yhfL, entF, torZ, araF, nfrB, kdgT, yagN, acnA, yqjC, ybaY, msyB, amyA, wcaD, yfcG, thiC, yidL, yidQ, csgF, yfdV, fucR, yhcF, tfaD, crl, fimD, yfbV, narG, ybiJ, ompX, sufB, mscL, yhaK, cydA, yohC, ybbJ, mdtH, sstT, ybhG, ybiH, ykfI, smf, ydcK, eutH, ybdM, yqhD, tauD, rhtB, yiaT, ybeM, relE, hchA, uspD, yqhC, relB, entE, yhbP, yicG, ygiW, uxuA, mngR, lon, rpoD, uxuB, ybdL, prlF, yneL, fucU, smrA, slyX, yhjY, ybeZ, ycaD, suhB, phnK, ydfO, tnaB, pyrD, ycjY, rlmA, glvG, apt, ghxQ, paaH, ygjJ, lptB, paaX, cdh, tdcB, wcaK, emtA, ygjI, arpB, racC, xylA, lldP, gluQ, yjeM, cusR, ygbJ, rsmF, hydN, ndk, yeeJ, mdtC, gspF, puuE, pnp, rpoE, ycjG, gsiA, eptB, yejG, yedA, rmuC, yieE, ybjX, groS, yadM, clpP, udp, yffM, ydeQ, gntT, frdD, higB, yjdI, yadL, rpiB, yffQ, argB, ghoS, fdnH, cbdA, dusB, potA, queA, mnmA, ygdQ, fldA, fabF, yaeR, entC, ccmD, ybjS, rpsU, aroK, atpD, purE, yjiH, hokE, rutG, aldB, yjbF, xerD, ssuA, dinB, beeE, kdpB, umuC, ybaT, basR, zapE, recA, rhsB, gpmM, cheR, yfjM, yddA, argD, sgbH, lplA, frdB, phnC, insA6, yejM, ydiY, eaeH, yafN, hyfC, leuB, pitB, torA, csgG, paaE, ylbF, ybiT, wcaJ, paaB, rhaB, panC, elaD, paaA, yfiP, plaP, recX, araD, yhiL, yjdN, iceT, flgI, yjcS, ydaT, sapB, idnT, ampG, gshA, yafO, ydcV, yicR, adeD, ulaD, purM, eutM, paaF, yrhB, menB, cusF, ycjO, yhiS, yfbR, tyrP, feaR, holE, glsB, ygeW, gabP, citD, yfbL, yciK, araH, exbB, pheP, maeA, selA, php, wecE, speD, ydjX, yhhN, aroH, araA, ygaP, ygeO, ttcA, yfiB, tdk, ynjH, yjjA, ycjP, xdhB, pdxB, gspI, udk, ettA, acrD, lsrC, yegL, hyfI, sbp, aslA, trmI, yehT, rlmH, treR, rhsE, ssuC, yohF, sgrR, ydfA, ykfA, mltD, yhdT, fliE, entH, hiuH, ccmE, tsaC, ygiQ, glcA, kbaZ, glnK, paaZ, ybjI, rimO, tam, nanA, yeaO, ftsB, entA, pflC, yhbY, yigG, yfdQ, rsmB, cheA, yeaX, paaD, tdcA, rnt, mlaA, mltF, tnaA, dicF, rsmG, dicB, mdtB, fimH, cynX, dcuC, birA, lgt, cysG, ytfA, eutP, panD, gshB, yfiF, tcdA, gudP, pyrC, atpB, yiaM, yicI, yeaW, ptsN, ada, qseB, cysB, ydiL, yhbX, yiaV, ddpA, fepA, phnH, nrfC, amiB, yfeO, iscR, yqfE, yedW, yigB, gspE, rcsC, caiB, hypF, ygcU, yggX, ycaO, hyuA, cspA, rimP, flc, glsA, yeaQ, ybdK, yqjD, ybgA, gabD, gmr, osmF, dppB, yehE, mazG, hemG, ascB, patA, astE, purH, rydB, ydcJ, yeaE, glnD, hemD, ybiX, brnQ, yceJ, nfi, secD, ppx, ydhC, iclR, yabI, fsr, ybgD, yeeX, zraS, yedQ, yidH, ykfC, rsd, dcuA, yobA, tatC, ybcJ, nudK, yafT, ybaA, hisD, chrR, yjjQ, rcnR, truB, ugpB, wcaB, ebgA, ycaL, ulaE, galP, yidA, oxyR, yegE, tsaE, yfjV, murE, pepB, yeiG, ecnB, aspU, uidA, ybdJ, fliG,

	gltB, ychO, yciQ, opgB, crr, pqiA, livH, yaiW, malZ, dsbB, arnA, yaaX, glk, yhjK, dcrB, acrZ, yjgH, lsrG, yhdP, yheS, hisC, gltL, priA, endA, chbB, ydjZ, yjeT, acrA, yqhA, ssb, gsiD, cfa, can, ygaU, gfcE, ykgH, yiiE, kdpC, ynbB, rhsA, ydiP, waaP, xylG, yhbU, yagK, mscS, nanM, yfdF, yiaB, pgrR, atoB, cho, waaQ, hlyE, ydjH, ycdU, ygiZ, xylR, yjeH, ais, tfaE, ompF, yqjH, fimE, yagM, yccX, yfcO, htrE, aegA, agaC, ycgM, ydhZ, clpX, setA, ybeX, zapB, ecpR, lysC, yfaZ, ybgJ, galM, frvR, ybeY, ydeM, ryjA, oxc, yhdY, ihfA, cohE, cbrC, yfjU, mnmE, bioF, yoaF, yffS, treC, fabB, hemF, yagH, ydcA, wzxE, nlpE, insF1, ompL, cysE, ybjP, yncE, tusA, proQ, gltX, dacD, ugd, exbD, sspA, mntR, ybjN, fabH, rplE, rpmJ, cyoA, ompA, gapC, pheM, rsxD, nupC, dppA, ilvB, ycfT, wcaI, wcaC, cpsB, cynT, ybdR, macA, aroP, ygeY, hyfD, csiD, sfsA, acrS, betA, dnaT, clsB, yoaD, glyW, nuoG, xisE, nuoI, nac, hupA, dedD, glyQ, micA, argW, serW, nuoM, glyY, uacT, yedL, pdxK, motA, yehI, lptA, hybF, rne, dicA, proB, yiaL, oppD, hycA, yadV, sspB, yfeW, ydiM, rpoZ, ravA, pepP, pyrB, ppc, hypB, yjbH, mcrB, ygiF, yahL, thrB, ybcL, phnP, glpB, pliG, kdgK, mazF, potD, ynjF, yecS, opgH, dinI, yjeN, rplY, ymdA, accD, hemE, rtcR, napF, yihQ, ppiD, rob, glyS, pbl, yqiA, rpoN, ydhR, cbrB, surA, ptrA, cydD, nirC, lsrB, smtA, yebY, pheT, phoQ, wcaL, ilvI, frvA, glnL, yciB, minC, cbtA, pcnB, paaC, elfD, lpxD, ninE, clcA, prkB, gcvR, fmt, zapA, insF2, murB, ldtE, ybcO, ydiF, nuoF, mcbA, nfo, sodC, thiL, yjiG, thrC, yebV, yfjZ, mog, arfB, usg, yegW, rspB, abgB, wcaM, motB, evgA, sapC, mngA, rlmD, fkpB, fnr, ecpB, mpl, hyfF, selD, yoaA, yheT, gpsA, cybB, mdtN, serC, tnaC, dppF, dppC, eptC, ydhW, yfdC, pgl, arnB, yahB, yedP, narW, ybjD, dppD, yiaO, lptF, lpxL, fhuA, ypjC, ubiH, araE, ynjC, map, aspS, hrpB, lolA, acrB, umpG, hns, rzpR, ygaH, dhaL, ssnA, amtB, xerC, astD, dmlA, ribF, ylbE, rzpD, uidR, potH, baeR, wecA, flhC, ydhS, narU, ybjL, vsr, yggS, trmL, tmk, hcaC, yahE, ynjA, eutJ, fhuC, micC, stfQ, ydcH, yegV, ligB, insD8, yneJ, ybjT, helD, spoT, ghrB, rarA, sgcA, ynfG, rodZ, ynjB, lhr, trmJ, yjbR, yidF, mcrA, mepA, rtcA, uvrD, fabG, ykfF, lysA, btuF, yncG, ygcW, yghQ, ivbL, ygcO, ybgL, serU, sfsB, thiG, yjaH, ygjK, gstA, yfdE, aroB, cheZ, fadL, gsiC, yjiN, ycfL, hisM, galU, sokC, yagB, mukB, metH, pka, alr, yphA, malT, rluE, mlaB, gyrB, erpA, tyrR, hsdM, glnA, torS, yodB, yqgB, secE, yhhY, pncC, yjhH, yrfG, yfgM, ybdG, epmC, sdaC, pldB, yagF, cueR, moaA, fliJ, murG, xdhA, yhhM, yceB, pheL, artI, ydfE, hokB, kdpA, arcZ, ygaV, chbR, ypjK, atpH, thiF, argE, ftsP, rpmG, anmK, yegU, yeeN, sfmA, znuC, ldtD, atpA, ycaP, ydcS, rutF, cyaY, alpA, trxB, rutR, zraP, ybgF, ytfQ, ybhR, bioD, btuR, paaY, yieH, rbsB, cheW, leuL, mscK, rhsO, cmtB, mlrA, yfeH, ybcV, yjcE, tauB, glcE, yijF, rraB, atpG, uxuR, trpE, glcF, ydcD, rbsK, yibN, ryeA, bcsC, ygcE, yagE, pgaB, glmZ, ycjT, yafZ, yhgN, plsY, yccU, sibC, nusB, mrcB, gntX, artQ, mdtQ, srlB, ppsA, yjdC, yihU, eutK, rlmJ, yjgA, mdtA, yciI, deoR, pal, ydaS, mazE, mltC, yehC, tfaX, artM, yedE, napG, narY, fucI, fadI, rpsT, galK, yfaS, eutC, wecF, ydeN, ilvE, ubiG, idi, mutL, wecB, yibL, yafP, rffG, murI, yjjU, ylbG, tdcF, tusD, dacB, adk, galT, ycjW, grxC, eutB, ruvA, yegZ, galE, ackA, ribE, yaiV, waaF, opgD, yjhB, yjaG, yiiQ, trkH, tilS, rhaD, ptsG, paaK, wecC, phoU, atpE, ompR, glcB, nudC, rbsR, ycgN, cvrA, ruvB, bglX, yecR, rssB, degQ, prs
**Nitrosative**	*norV, norW, ytfE, adiY, alaE, hmp, adiC, yohJ, hcp, yohK, ybiJ, yhaK, bhsA, hcr, ldhA, grcA, ybfA, ndh, cydA, ygbA, yhhA, soxS, mntH, uspG, cydB, qmcA, feoC, mqsR, tehA, feoA, bssS, sufD, yfdY, sufS, ypfM, yjjZ, feoB, ychH, yjgX, copA, sufA, nrdD, sufE, grxA, sufC, dps, sufB, psiE, yohC, tomB, yhcN, hslJ, cdd, mntS, ybbJ, yhfG, ychE, garP, mdtH, zntA, yneM, ynfM, bfd, ybgE, ygiD, nirB, ybjM, soxR, eco, sstT, ybhG, yfdT, uspB, yidK, ybjQ, torI, yccT, yeeY, nsrR, yccM, ybcW, ynfD, asnA, casE, tehB, appY, yedK, yrbL, ilvL, ybiH, fic, dinJ, lafU, blc, ykgE, yecH, zur, bssR, iraD, ygfS, yhhW, ahpF, uidB, mokC, yqaE, yibI, ymgE, ybhL, fruA, mqsA, ykfI, ilvX, emrD, ycfJ, yjfN, eutR, csiE, yjcB, smf, ydaW, yjaB, nemR, ybhF, rnr, nrdG, yfhM, ytfL, gadX, csrB, nrdI, ydcK, ryjB, ascF, rof, rayT, mscL, yfiR, yeeP, ygbN, frdC, nrdH, fes, dtpB, yodD, yacL, smg, ilvM, clpS, pdhR, fepD, chaB, ypfH, yrhC, gltP, flgM, sseA, mgrB, ydhX, yafY, yfcI, artJ, dkgA, yjiY, yedR, ybaV, bolA, hofB, ybdF, xisD, ygiN, slp, gspO, narH, ompX, yqiJ, ydcY, amiD, yedY, ilvG, yffL, yibT, ydiE, eutH, ybdM, yqjI, yqhD, fhuF, pmrD, yahC, clpA, yddH, yecA, yifB, qorA, yaiT, rimJ, osmB, ybgS, focA, panE, yddM, cbpA, focB, yhcH, mhpF, tauD, fsaB, yhiI, rhtB, dtpC, fecI, yhcG, arcB, cbpM, malG, uspE, yiaT, yeaH, pgpC, yeaG, narG, hha, sbmC, sulA, ygbF, ydgD, exuT, ybeM, ydjA, ybdZ, leuO, yaeP, ycgB, aidB, argI, yfiL, mhpB, yafE, proP, hspQ, yaeH, ybhS, exuR, dgoK, yejO, yjcZ, yoeA, amiC, insM, xylH*
**oxidative**	*arsB, emrD, yhfX, stpA, pyrF, emrK, fes, bcr, xseA, thiB, fhuE, entS, gspK, rlmG, mdtO, fepB, entE, yhbP, yicG, fecD, cirA, ygjQ, mhpT, sgcC, osmB, yfdY, ydeP, yhfY, ycgZ, ydeT, yqjI, corA, yliE, dmsC, yphD, nirB, torY, yeaR, nirD, gspB, ymgG, yidB, fimG, nrdE, rclB, yghO, creD, yjhF, yfbP, yicJ, yfaH, frvB, ygeF, yqeK, yegJ, yliF, yhhZ, yigF, apbE, yqiH, yigI, mdtE, cysH, ldtB, mdtJ, ycgX, mdtI, yceA, cysD, mdfA, yedN, yciA, yfcU, yifN, pfo, yidZ, cysA, gspG, murJ, ygfK, ydhT, zinT, yghG, tsaA, rep, rhsC, trmA, nudE, acpT, hybB, ytfL, fepD, ascF, ykfB, wzxC, opgE, rseC, lgoD, mlaE, ycaD, suhB, phnK, ydfO, tnaB, pyrD, ycjY, rlmA, glvG, apt, ghxQ, paaH, ygjJ, lptB, paaX, cdh, tdcB, wcaK, emtA, ygjI, arpB, racC, xylA, lldP, gluQ, yjeM, cusR, ygbJ, rsmF, hydN, ndk, yeeJ, mdtC, gspF, yidK, proV, yadE, pinR, yehY, setB, gspC, arnT, uhpC, ycbU, bisC, gspJ, viaA, xylE, yjfF, caiT, ycfQ, cusA, rutD, ybgP, yhgA, frlC, hycC, ygjV, hyaB, yiaY, frlB, yieL, yegX, nudI, yeeS, chiP, ygaQ, cmtA, yaaJ, cmoA, phnE, ycgL, yiaN, ulaF, gspL, tatD, yqcE, yeaP, glxR, mutH, ydiK, arnE, rhmT, yiaW, cusS, kdpE, phnF, yajI, satP, mhpE, rhtA, ompN, yfcC, gmhB, yfgF, mqsA, alaE, yhhA, ycjF, ygaC, zntR, higA, narP, ycfJ, ymgA, ymgD, mgtA, mutM, yaiY, cnu, yhcN, ldhA, iraD, yjfY, yibA, uspG, yiiX, glgS, araC, mlc, yfdT, yjfN, torR, yffO, ynfK, yeaJ, ybdH, gltS, yphH, metB,*

	dgcZ, ariR, nrdH, yebE, ydeR, nrdI, tfaP, argF, yahA, yqfA, yhhQ, gntP, csgB, hokD, arsR, cspD, ycbJ, norR, hcr, casE, gntK, wecH, dmsA, rusA, mcaS, phoP, argA, eamB, ccp, flxA, yqjK, yhfW, yicH, uxaC, lacY, fruB, ytfH, yffN, pgaC, yhfZ, ydaC, yehH, yqaE, bluF, fbaB, leuE, sixA, ygeN, ygeQ, fecI, gadW, nrdF, arpA, ybfG, ycaC, rmf, treF, tusB, bglB, recN, yojI, yjhE, yfbN, tfaS, frc, cusB, ydbA, yedV, ymfD, yifE, grxA, eutR, sra, yihF, bglF, recF, cadA, essQ, rlmB, dsdC, yidX, flgL, yncD, appA, yadD, umuD, yffB, tdcG, dusC, yaiO, sgbE, ytfI, ypjA, sxy, eutQ, yfjD, yhdW, ryhB, waaZ, eutD, yqeB, ydjF, argH, srlA, ynbE, ycgH, yehQ, wzzB, elfA, yhfL, entF, torZ, araF, nfrB, kdgT, yagN, blc, tehA, ybaV, yjaB, yohJ, xylH, waaH, dadA, yjiJ, phoE, garD, yhiJ, glvC, gltA, glgA, elaA, ilvC, alsA, yiiD, ytfJ, ftsA, rclR, metA, yqiK, sgcQ, ydbL, gudD, narJ, fdnI, sad, cadB, yhjX, ypjD, gfcC, ydjG, araB, ydfD, xapB, yiaA, pppA, intK, fucK, yfcQ, mtfA, aldA, maeB, yghT, sfmH, yfaQ, yehB, aes, ushA, yhaI, yfcP, ydaY, frwB, yggF, borD, tag, ylcG, uidC, yaiP, aaeX, ralR, preT, yjgN, fucP, ydcC, yfdL, fimF, kilR, ydiQ, yfdS, yjfL, yqeH, xylF, caiE, ydeO, yijE, ydjE, frwD, yjgL, yqiI, allB, yghF, hflC, ygiV, yhdZ, nudL, yeeW, iraM, yeeD, cysI, holD, rimI, ygiM, ydhJ, cspF, srkA, tpr, ubiX, ydjM, fadD, yhjJ, rnpA, gpt, yeeE, cysP, cysJ, emrE, ybfE, lipB, mutY, entD, yncI, yagI, fadB, yqiJ, lexA, yhbE, maa, rttR, yhdU, ygbE, rsxA, ydgK, ydjN, trmN, yggI, hcaR, yidC, ybgC, mioC, mntP, ymfM, efeU, cysU, yciW, yebF, yiaD, elyC, gnsB, thiH, pabA, folA, ybjG, queD, yjiR, ymfL, uhpT, pth, ibaG, rfaH, ytjC, mltA, wcaG, fecA, atpI, mreC, glpE, menA, slp, ydiE, yedK, yfcI, yddH, mhpB, dinD, cspG, ydiH, potF, wcaA, glmS, gpp, sfmD, aroM, yecT, clsC, uvrA, fliR, recQ, dosC, wcaE, greA, essD, proY, infA, ymjA, opgC, ydhI, phoA, alkA, wzyE, yegS, yeiH, yebG, yafK, glpG, mscM, fis, ybhB, rstA, yhiM, ybiV, agaB, ymfR, jayE, cysW, manA, stfP, lpxT, rluC, ybjE, asnC, yehP, hyi, yjgM, yneE, rph, agaD, gtrA, tolQ, cutC, rsxB, ydcF, rhaM, tsaB, yibQ, yidI, fhuD, nagA, epmA, atoA, ybjO, ybeQ, fecC, mreD, wcaH, yddK, yedJ, rnhB, ybjJ, prpD, entB, yedS, mepS, nuoK, secG, nikR, idnO, idnD, ilvN, tmpR, yadC, mhpR, yeaY, yjhD, yehM, yfaE, ydgC, ycjV, ychF, ratB, hdeD, yecM, rhaS, gspO, ndh, zntA, sulA, ybhS, adiC, garP, asnA, ybhL, nemR, nrdG, ydcY, dgoK, ygaY, ebgC, nepI, mqo, dctR, proX, betT, wcaF, acs, tdcD, rzpQ, puuB, phr, hofM, fiu, fepG, ppdD, sslE, ymdB, fepC, alx, ykgA, lgoT, yjfK, sdiA, nanT, ytfT, dbpA, lyxK, fecR, yrhA, rnd, ybgQ, ydiU, lnt, ydgI, tusE, cysN, hyfR, adeP, rdlD, yihG, dinF, hsrA, pgm, alaC, araJ, msrA, insZ, rybB, ycdZ, ydiV, fdnH, cbdA, dusB, potA, queA, mnmA, ygdQ, fldA, fabF, yaeR, entC, ccmD, ybjS, rpsU, aroK, atpD, purE, yjiH, hokE, rutG, aldB, yjbF, xerD, ssuA, dinB, beeE, kdpB, umuC, ybaT, basR, zapE, recA, rhsB, gpmM, cheR, yfjM, yddA, argD, sgbH, lplA, frdB, phnC, insA6, yejM, ydiY, eaeH, yafN, hyfC, leuB, pitB, torA, csgG, paaE, ylbF, ybiT, wcaJ, paaB, rhaB, panC, elaD, paaA, yfiP, plaP, recX, araD, yhiL, yjdN, iceT, flgI, yjcS, ydaT, sapB, idnT, ampG, gshA, yafO, ydcV, yicR, adeD, ulaD, purM, eutM, paaF, yrhB, menB, cusF, ycjO, yhiS, yfbR, tyrP, feaR, holE, glsB, ygeW, gabP, citD, yfbL, yciK, araH, exbB, pheP, maeA, selA, php, wecE, speD, ydjX, yhhN, aroH, araA, ygaP, ygeO, ttcA, yfiB, tdk, ynjH, yjjA, ycjP, xdhB, pdxB, gspI, udk, ettA, acrD, lsrC, yegL, hyfI, sbp, aslA, trmI, yehT, rlmH, treR, rhsE, ssuC, yohF, sgrR, ydfA, ykfA, mltD, yhdT, fliE, entH, hiuH, ccmE, tsaC, ygiQ, glcA, kbaZ, glnK, paaZ, ybjI, rimO, tam, nanA, yeaO, ftsB, entA, pflC, yhbY, yigG, yfdQ, rsmB, cheA, yeaX, paaD, tdcA, rnt, mlaA, mltF, tnaA, dicF, rsmG, dicB, mdtB, fimH, cynX, dcuC, birA, lgt, cysG, ytfA, eutP, panD, gshB, yfiF, tcdA, gudP, pyrC, atpB, yiaM, yicI, yeaW, ptsN, ada, qseB, cysB, ydiL, yhbX, yiaV, ddpA, fepA, phnH, nrfC, amiB, yfeO, iscR, yqfE, yedW, yigB, gspE, rcsC, caiB, hypF, ygcU, yggX, ycaO, hyuA, sufE, yccM, yhcG, ybdZ, adiY, dtpC, hcp, ynfM, yccT, ygfS, yibI, yeeP, frdC, dtpB, focB, yhcH, malG, leuO, insM, obgE, bluR, deaD, acnB, rplA, proW, ynfN, melR, gspD, rhlE, dinG, trpA, dtpA, aslB, panF, yehR, zraR, rrrD, serS, yhfU, ppdA, flgK, ampE, aaeB, prfH, yqeJ, metQ, dmsD, yiaJ, mtgA, kefG, yrdB, glxK, rssA, uxaB, serA, fadR, ryfA, pepD, rseA, rhtC, acpH, ybgO, glmM, rtn, rarD, ykfJ, ydhK, ygeX, yebQ, yhaM, rnk, yfgO, yhfS, rcnA, queG, yjfI, chbC, yjeO, tldD, rhmR, yejH, yegQ, narK, gatD, metE, efeO, lysP, gatY, cyoD, dauA, rnc, ybaK, pdxH, ycfH, napC, gph, chiA, ldcC, yaaU, ydiS, cynR, pdxY, nrfE, opgG, yafF, hyfB, uhpB, oppB, yjiK, yfjQ, pflB, oxyS, nuoJ, yagG, ybiR, flgC, ynbD, pqqL, ygcS, ynjI, ycjD, fsaA, yfbT, nanE, prpR, speC, rhmD, ygeH, ghoT, nikC, yciY, aas, coaA, yiiR, rnlA, sgcB, fdnG, uxaA, ribA, dgoR, ybiB, cysC, talB, mdtM, rlmL, ydiR, ybbW, fryB, eutA, fadH, yhaB, yjjW, yiaK, iscA, yajL, purU, yceM, elfG, flgB, sgbU, nusA, hisQ, modB, yidE, fliD, lpp, ypdE, yjhU, yhfT, mhpA, hyaC, ydjI, hycI, yeeR, prpE, yjfC, yacH, prpB, hofN, rclC, mokB, napD, flhD, yegT, nupX, oweS, atoD, cheB, pflD, dgoD, fdhD, ybiA, ydiO, amn, frlD, asnB, agaV, yqhH, ulaC, iscX, yjgZ, rutC, yfbS, ygcQ, cysM, frwA, hycF, hemA, yahI, yoeD, hycB, yfdR, kbaY, yqcA, ascG, ycbX, yafJ, bglH, ymiA, priC, xanQ, torT, phnI, phnJ, yidP, hyaA, ydjL, ydiN, zwf, dapE, ydiT, gspH, ynfA, gntU, rtcB, prpC, ecpC, phnL, gltI, yhbQ, yeaV, intA, wcaN, yjgR, alkB, ypeA, ygeK, pyrE, ydfI, gatR, nohQ, tpiA, ynjE, fpr, lomR, ytjB, yahF, evgS, ccmF, ycgY, potI, yaeF, dcuD, bglA, yjiC, yghX, ybiW, yedF, ynfE, ddpF, nadC, yqeA, nikA, yghS, yegK, xdhD, hypE, paaJ, tus, aroC, ddpB, agaS, putP, intG, arnF, ycaI, cbeA, cpxA, yhhT, yfjX, phnG, cheY, hycD, gsk, frwC, rpsF, lpoB, scpB, fldB, eptA, caiA, hofQ, yjjP, yahJ, csgC, rng, fumA, purK, ecpA, flgH, bcsZ, hybD, hyfA, dgoA, rspA, fetA, ydjY, gpr, argK, mepH, glnB, intQ, rsmC, sppA, serB, narV, aaeA, tdcE, cusC, recJ, yfjJ, paoB, acrE, hybA, yihP, argP, ppk, xdhC, argX, flgG, cbdX, yegR, cysK, ygfT, citG, cspC, yqeG, clcB, scpA, xapA, ldrD, yedZ, yjbL, ybgK, flhB, yddG, yicN, purC, yghD, alsC, insP, yfcJ, yifL, yadS, ybhK, ghxP, ydiB, yoaB, purR, yfeR, hrpA, paaG, cysS, tynA, ldtA, elfC, yebT, yjfZ, pntB, yjfP, rplI, pqiB, yhjV, malF, nth, fhuB, hycG, pepN, thiM, napH, eda, dhaM, hyfJ, argS, yjjG, agaW, dsbD, ygjH, rlmC, hcaE, nrfG, scpC, citB, recT, ygeA,

	yddW, yggM, rsfS, srmB, dapD, nohD, flgN, hyaF, rlmN, ygeG, ygcP, rihB, dusA, rlmF, nuoN, moaB, abgR, msrC, ychN, yfcA, ydjK, yhdX, yqgA, accA, yddL, yeeT, sfmF, yjbB, ybcI, yeiR, ygdG, ycgV, recR, fieF, dnaC, yobD, mdtP, yraI, yjjZ, kdpF, aaaE

Table S7: Combined list of downregulated genes in all samples of each stress

**Stressor**	**Downregulated genes**
**Heat**	*cysP, cysJ, rnpA, cysH, cysI, ydjN, sthA, codB, codA, nuoF, livM, thrC, nuoG, nuoM, rnb, nuoE, gatD, opgH, murF, prs, cyoB, aroA, cysD, tktA, yhbJ, plsX, pdxA, spoT, folE, asd, murE, serC, glnA, guaA, trmH, cyoA, opgG, adeP, dppF, waaF, ompF, purD, uraA, pyrI, pyrB, carB, nuoK, carA, purL, potD, gdhA, nuoJ, nuoI, nuoH, speD, nmpC, nuoL, nuoN, ygdH, cyoE, purN, gcvT, potB, aldA, aceE, livG, ilvC, gcvP, oppC, livF, cysN, cysC, gltD, gltB, cyoC, cyoD, pheT, purB, purM, glyA, nuoC, ybhC, pyrC, pyrD, xanP, purE, oppB, lysP, purK, accC, thrB, thrA, atpF, trpE, lysC, metE, gatC, pnp, livJ, thiF, yciW, atpA, gpsA, thiH, trpB, fbaA, cysK, serA, pal, sodA, thiG, acnB, metH, trpA, cysM, hisC, hisG, glgB, hisH, rho, gatA, plaP, sdhC, sdhD, pcnB, pykF, gatZ, trpD, sucA, sucB, ansA, mreC, pta, hisD, yidD, dppB, sdhA, cysU, thiS, dppD, thiE, nupC, sdhB, nlpA, aceF, secA, wbbJ, speE, maeA, dppA, leuA, hisA, leuB, yigB, ilvH, dppC, leuD, leuC, xerC, flgJ, flgB, fliL, flgH, pntA, malE, hsdR, purT, gtrS, gcd, ydiJ, ndk, oppF, dicA, ppc, pntB, ribB, hisF, metG, gnd, nhaB, bamD, yncE, uvrY, truB, purH, purF, cvpA, purC, tsx, efeO, waaC, rsxC, pfo, borD, alaA, icd, potA, mqo, yidC, ymfA, folD, znuA, oppD, ppsA, glf, ydgA, rne, wbbI, gcvH, ygjP, epmC, ghxP, xseA, katE, hdeB, hdeA, rhlE, slp, rsxG, racR, hdeD, fhuF, purA, lpd, gmk, ybiC, rsxD, rimM, yfcL, rpoB, rplQ, atpC, yqcC, yhbY, tyrB, gsiA, upp, suhB, yhgF, tsaB, guaB, gsiB, ybgF, gatB, rpoA, aceB, ygiQ, ydcI, yceA, sbp, rpsA, fumA, ackA, dosC, exbB, cysA, gltA, cysW, mtlA, yejL, intZ, atpH, rpsK, rpsD, atpG, rplB, lolC, ilvB, sucD, yqgF, trpC, yidA, fabG, nadC, dtpA, atpD, nfi, pheA, menF, deoD, ilvI, aroH, yeiG, rbbA, rpmF, zitB, ychJ, aceA, sucC, thiC, bcsF, nudB, serS, glgX, gntR, secM, bcsG, rpoC, amn, mepA, yjaH, pdxY, ptsP, ynjI, citE, accB, ycaO, eptC, yagU, hypB, fliM, ansB, hybO, flgD, focA, fliN, flgA, hybA, fliG, flgF, flgC, fliP, evgS, ynjE, ravA, fliK, flgG, flhA, flhB, ymdA, fliI, cheR, emrB, ybgA, yjiM, artI, melR, malK, dctR, kup, pka, flhE, flgE, flgI, yfjL, yecR, gltF, yceI, fetA, yobA, sapB, ydiH, pyrL, waaL, yedE, mprA, fruB, ptsG, hypD, ymfI, artQ, fecC, fliE, fliR, mcrA, ruvC, rcsD, yieP, rbsD, livH, argG, yeeN, yebK, cydD, yeaD, sdaB, clsC, uvrC, yjfN, glyS, yjiX, fdx, yhjE, btuB, yedF, ebgR, epd, waaQ, blc, aroF, ampD, rhlB, ymdB, yecC, phr, mdtE, rnc, iclR, yfcU, ftsX, yciT, ebgC, yfjU, kch, cheB, pitA, gspD, gspF, fliO, ycbL, yebC, mraY, livK, ycbK, fadL, gtrB, frmB, thiP, mipA, pheP, ampG, yeiP, metC, thiQ, lpxH, lipB, hsdM, artJ, argD, yqgB, mgtA, speA, lpoA, dapB, murI, artP, gph, potC, yibQ, ibaG, ybiT, wecB, ridA, pbpG, moaB, gsk, menH, ygaH, cspA, yfjW, plsY, gcvR, yjiY, ynfG, rsxE, glnQ, glyQ, yeeE, yjiN, rpsS, yccT, gatY, cheW, hpt, yhbE, ybiU, rluA, yeaK, menD, cheY, yheO, ybcJ, yihI, argA, nagC, csiE, agp, uup, yghB, glnL, ydiB, ubiI, wecA, ypjA, dhaL, nohD, tpx, mreB, zur, moaA, cspC, fdoG, yejM, fetB, torT, ybaP, hemB, iscX, ggt, ybdF, yigL, gspJ, ppx, fecI, hypA, speB, gapC, rstA, lolD, rsmA, nlpI, yifK, zinT, hyaF, yeaY, yehY, ybfP, skp, recO, ydcL, rssA, flgM, fruK, yqiC, ydgH, secD, recX, ygiB, galR, mzrA, insA8, hisM, minC, yidB, rffH, slyD, crp, yqgE, aspC, glsA, yjiG, yghU, hisQ, pdhR, cheZ, eutJ, ubiJ, ycjP, yciI, fhuA, nanR, hisB, rffG, gltT, yjdC, rimP, rspR, hypT, dnaT, fldA, ygjH, alkB, folP, psd, fdhE, era, wecC, gatR, pepE, ykgM, fliY, yrdB, tatD, tadA, fabH, yejF, gadW, yraJ, aroP, yrdD, allD, yagN, cra, artM, yfcA, yebW, yeeA, pspF, yjgA, tsgA, ydcP, yeiB, prfC, recG, dusB, rplD, ubiX, arnC, hycG, fdnG, folK, yeiE, nohQ, clsB, yhjX, yeaH, nuoB, hydN, potF, malP, oppA, yeeR, potG, lhgO, adhE, gsiC, holB, ybiH, pflB, poxB, yjgL, hypE, ygbM, solA, yiiX, yncD, rlmA, fadI, tsaA, ispA, kgtP, nuoA, bluF, rplC, typA, gluQ, sanA, tap, citC, yiiQ, yfbR, pyrG, nemR, ribA, ydeA, ybhG, yijO, potH, fecA, deaD, fadD, bcsQ, cspE, argC, yfdC, yacC, ydcN, proY, sppA, yehP, dauA, cmtA, wzxB, ybiR, lit, opgC, rhtC, rlmG, ppsR, grxB, kdsB, hyaB, citD, yafW, fkpA, rimL, yfcG, fixC, hycE, mdlA, sufA, rssB, hycA, hyaA, wecD, sapA, yegD, phnP, yecN, sufC, rpsP, phnN, yhdW, ilvN, citF, envZ, cheA, yafQ, yigF, yebS, rfbC, ptsH, ydhS, nudJ, trmJ, dhaM, rnlB, fabD, trpL, csiD, osmF, pgaC, ybjX, flu, argH, bcsE, mtlR, galT, hycC, prmC, gabT, xseB, gsiD, mscM, hrpA, tyrT, acrZ, rfbA, basS, yddW, tyrV, fusA, astB, yghX, lpxB, rsmG, arnT, nusA, alsB, yjfP, rnd, aroG, putP, hyfA, yaiI, yfdX, ycbZ, fdoH, folM, murJ, yjjV, yddL, mnaT, nhoA, msbA, yebV, uidA, yaeI, dam, aer, cbeA, mntP, dnaX, ybgC, galM, rseB, yehW, npr, ydiK, tatB, purU, hycB, dnaB, ycfH, yedP, bcsC, yafJ, yddM, rstB, ydgK, aroL, rhsO, bcp, yhhN, cusA, ydhF, apaH, secF, adiC, hyfR, sugE, hemE, lolB, acpT, pgpB, prmB, rsmB, hyfG, ybaB, ispG, ybdK, yidK, map, rluE, ispE, dtpD, pgpC, rodZ, cmoA, rspA, sufD, nudK, nrfD, cutA, sufE, yccA, dbpA, dapD, yafT, lysS, sufB, fnr, bdm, yceF, tsaC, smpB, exbD, cpdA, yhfG, baeS, yciC, yceB, yjaG, tusC, yjjG, glyV, dapF, pepB, pgk, yejB, dosP, ybgP, cysT, pabA, yedK, wbbL, yqaA, tyrU, hycF, infB, ypdA, insA2, tolC, rsuA, bfd, aspS, mgrB, yeiR, ymjA, ftsE, rsgA, viaA, ffh, mrp, alaC, yibF, ygbA, udk, mlrA, yfdR, nadK, fliH, wecG, fau, ybhP, yhbW, argO, yfiM, pdxH, selA, queF, rnk, fdoI, prfA, queG, rtcA, ada, asnC, ygdQ, ybaO, nemA, ytfL, rlmJ, zapA, bluR, fhlA, ylaB, ycfL, lpxP, cbdB, dadA, epmB, ydhL, yjeH, proP, yhdE, tam, tusD, yfgG, phoQ, sufS, yccU, infA, avtA*

**Cold**	*plaP, purT, uraA, ansP, sanA, stpA, nmpC, borD, mutS, guaB, purL, mdaB, cysJ, purB, thrA, pyrD, thrB, pta, asnA, fdnG, yfcD, purD, yagI, yniC, yqhC, purM, yedE, puuB, grcA, ridA, glnA, nuoE, argB, rimO, hipA, eutK, metK, groL, moaB, narY, thrC, eutC, slmA, gatC, recG, feoC, dut, ptsG, ydcP, rlmI, yceM, intS, groS, hycG, htpG, bglX, fbp, nuoL, yiiQ, gdhA, yehT, hycH, pbpG, proB, dcp, hchA, trxB, ackA, ybhG, marA, nagE, yfeH, dfp, ftnA, nuoJ, tsgA, pgi, bioD, gpr, gsk, pykF, ydiP, pepA, rpoD, ygaH, dapA, potH, frmA, pyrI, pyrB, upp, artJ, pstS, ompT, cysN, yghG, purH, cysD, cysH, codB, carB, yahK, carA, yccJ, cfa, pfo, pstC, feoB, katG, tsx, argS, moaE, ltaE, yjfC, murD, dhaR, yhjX, holB, tynA, aaeR, fimI, yejK, argG, yeiE, metR, uvrC, yjiY, yliE, efeO, ugd, sdaB, yliF, iraD, sdaC, gntT, argD, yegQ, ybhC, lsrR, fimA, clsC, yghF, yiaD, abgR, fliM, cysP, fecA, metN, fimC, phnF, wcaL, malE, feaR, alaA, ppsA, rcsB, potF, appY, yfdC, mpaA, sodB, tyrS, lrhA, ydjN, ycaC, cysC, yphH, argA, pqqL, roxA, mhpF, codA, flhD, metF, csgF, rluC, cydA, glpB, otsA, yeaK, fecE, xanP, entF, sgbH, ydaM, mgtA, yeeE, csgE, paaZ, flk, argC, topB, sieB, yqeI, ypfJ, fliK, fecC, ybjP, ilvC, treB, fdnH, nuoN, fadE, yhjC, mtlA, copA, yeaD, purR, yifE, fixX, paaB, cueO, yegE, glnQ, yagU, pntA, rtcR, cysA, pflA, atpF, thiM, tdcE, cysW, pliG, kdpD, metJ, ilvB, hycE, hisI, purN, ccmA, ddpD, purE, galS, hemL, oweS, flhC, hmp, talA, stfP, fdnI, yeaH, araA, ygfZ, atpE, nohD, kdgR, purF, hisD, treC, gatA, emrB, yfcZ, araC, yebE, cysI, paaX, ydcJ, yeeR, cmtA, ppsR, yneO, yhgF, tauA, citF, moaC, hlyE, fabB, rluA, proY, exuT, artI, wrbA, kdpB, glnP, mepH, napC, djlA, minD, glyT, yddG, potE, napB, nuoI, ycjW, artQ, mfd, hybC, yihI, dsdA, nupX, tktA, yfcS, gfcE, yhaH, gatB, atoS, ycgR, grxB, glmS, glnH, yiiS, ybdL, agp, umuC, menD, fecR, nuoH, dnaK, yejL, ypdF, ybbN, nanM, yhaK, yebF, moaD, pck, gatY, argI, rimJ, pyrC, ilvN, hyfE, ushA, suhB, gltA, ydgJ, guaD, yeiB, hsdR, thiB, yffR, ratA, mppA, cho, yaaA, livJ, potG, pppA, pstA, potI, nac, nrdA, flhA, uspD, nlpC, treA, yddA, focA, tyrR, yfjR, cysU, azoR, clsB, nohQ, preA, flgG, malP, poxB, nuoK, yebK, yfhH, yciW, yceH, ygbE, ybjS, ybjO, ydcT, argH, nuoF, yegH, yeeJ, nupC, lolC, matP, fimF, glnK, pflB, katE, csgD, dhaM, emrA, yqgB, yphG, nuoG, ycaK, tdk, gmr, yqhD, yacG, gcvP, ralR, tufB, ulaC, ydaT, ampE, aldA, ghxP, rsmD, yjeM, yjbG, iaaA, dusC, mrdA, deoA, yqeJ, ybjX, tufA, mog, wcaK, mobB, rnb, metE, yedF, yjiT, opgH, yciG, ybaE, yebQ, adhE, ccmB, livF, glpF, ydjE, atoD, mtfA, tolB, srlB, leuQ, yiaJ, murI, macA, argT, metC, lysC, mipA, bacA, murQ, hisJ, mngB, bioB, trpE, yedQ, aceE, rbsD, metA, eutM, glpK, ynbA, purC, yedL, ycaD, tktB, cydB, hemB, hyaB, leuT, flu, deoC, nudI, thrT, kduD, ydhT, napG, glpQ, leuV, gatD, mglA, dnaQ, dsbG, yfdT, glnL, dcuA, rutA, yggM, rzpR, lipB, wcaM, feoA, modF, hpt, yfcE, stfR, yagE, yggX, leuP, zapD, ruvB, queC, lysA, yecE, yfbM, trmH, rssB, nrfA, guaA, glyA, putA, potD, pntB, yihT, gabP, ychA, astD, trpD, hybE, nadA, prs, wcaJ, arnC, yfaE, dmlA, pspA, yjgL, ynfG, sapA, ydhS, yahA, lpoA, phoU, marB, yfhM, ldtB, tdcC, uhpA, rplF, nuoC, osmF, yneJ, ais, yeeD, nikE, gatZ, cysK, phnN, norR, nagZ, dapB, yobD, torZ, rfaH, yfbS, yjhQ, napA, frdA, cvpA, solA, adeD, tpiA, intR, degQ, paaH, hemN, metB, fliN, yciF, nuoB, ttcA, narL, eutB, gsiC, ybaP, trmJ, hda, eutL, sdhA, ymgA, ydfE, ybdR, ldhA, yahJ, yhhL, dkgA, galE, gabT, yebT, nanS, speA, yeiH, recN, panD, ynjE, mprA, psuG, yjiX, prmC, yghA, yejM, ccmC, ptsH, cohE, ompC, glpT, uidC, dicA, prlC, yohF, ycbJ, narH, livG, fetB, ddpF, mdtB, aroD, ybcH, ydcK, grxA, ptsI, ydjJ, cof, metG, cydD, pepD, yehB, frlC, thrU, kdgK, mdtA, tdcD, fruB, narP, selD, gtrB, yddW, rep, casC, yajR, dicC, ypjA, yghX, galU, pykA, arnB, msyB, gsiB, metI, opgG, lysP, rbsA, ydhP, serA, iraP, ybiY, ampC, lgoR, dgt, rpoA, livM, rapA, galT, yeaC, srlQ, gsiD, ynbC, panB, glmU, pdxJ, tyrA, hycI, ykgA, uxuB, ybiB, yhdZ, gapA, bcsE, yihL, lolE, purK, minE, manX, wcaG, narZ, uxuA, hybF, trpS, ygbM, ybjD, ygbK, yjgZ, rcnA, ycgB, ravA, rlmL, uxaB, proC, ddlA, mobA, hypB, mlc, nlpA, umuD, icd, nfsA, flgA, yhdW, ompR, ccmH, ydfI, glpA, afuB, lpxH, yajC, folK, cyoB, fadH, uof, dacA, menA, gcvR, yigF, yegK, yiiF, ygaC, mglB, gabD, sdhB, yjhY, ycfZ, rhmA, ebgR, thiQ, ogrK, lhgO, aspA, entS, dmlR, gcvT, btuB, fadR, opgB, kefF, fabI, ydcU, ybiH, ygjP, nfsB, moeB, asr, ygbN, infC, agaA, fis, moeA, rdgC, yfhG, udp, flgD, recE, gtrS, flhE, ybiU, glmM, ybaA, pepT, oppA, rsmF, yccM, mltA, thiH, aroF, yqeF, ulaB, yfaS, ybhF, emtA, hycC, dhaL, dcuR, ydiB, dsdC, cpxA, fadL, yoaD, ydcF, narX, cbpA, glcD, asnB, yfaL, amtB, ymfI, hyaA, cynT, hslU, sucB, ybjC, tpx, smrA, rimK, metL, lsrC, artP, oppC, rplX, proA, znuC, cdd, eutR, ygiF, pstB, yajQ, glxR, kefC, alsB, yeiI, sbcD, nei, thrS, sdhC, xylB, ybdH, yfbT, gph, arnT, frdB, yhhX, yrfF, rplE, hypE, manY, hydN, fucU, yaiC, hypD, sucA, valS, yhjB, ybgF, sdhD, sucD, prmA, yraQ, flgH, pfkA, ybhM, bioF, dcyD, csiD, ybjT, rhaT, fecI, citB, ydhK, trmA, nth, flhB, gltI, glgA, patD, panE, zwf, ansA, basS, bioC, cysQ, yqiA, nuoM, corA, ung, ttdR, bcsA, trmI, gspJ, pdxB, fdoG, rpmC, hemH, ybjJ, uacT, pyrF, ytfA, rsmJ, ynfL, ylaC, rsmG, yqhA, rplA, kdsD*
**Antibiotics**	*ompF, ycbJ, feoB, flgJ, feoA, frdA, ydeN, yjjI, ynfK, lamB, yjcZ, codB, purD, codA, uraA, ycaO, eptC, pyrI, pyrB, carB, nuoF, yagU, hypB, fliM, flhD, nuoK, yehT, ansB, hybO, nrfA, yfbS, ttdR, yccM, hybC, frdB, flgD, focA, fliN, flgA, hybA, fliJ, narG, gadE, napD, grcA, fliS, flgB, fliL, bssR, tdcA, napF, ompW, ydhY, abrB, yhbU, dcuC, yqgA, nirD, ynfE, srlR, ydjY, ydbC, yhbV, nikA, frdC, bioF, fliF, fliG, flgF, flgC, cstA, ydjX, malT, nrdB, carA, purL, potD, bioD, fliP, evgS, ynjE, gdhA, livM, nuoJ, nuoI, nuoH, flgH, pntA, ravA, fliK, flgG, flhA, flhB, speD, stpA, flhC, ymdA, fliI, thrC, nmpC, nuoG, nuoL, nuoM, nuoN, ygdH, ycaC, napA, lgoR, narH, napG, malE, hsdR, cheR, atoS, ybaE, napB, cydA, murQ, cydB, emrB, ybgA, yjiM, artI, melR, malK, dctR, kup, pka, flhE, garL, napH, cyoE, gldA, ysaA, ompT, yahN, garD, yqeC, ydiL, adiY, melA, fliT, ygcW, ydiP, frdD, gudP, garP, ghoS, narK, nikB, tdcB, yehD, frwA, cdaR, yjjP, yniA, yecH, ccp, yghF, ygjR, hypC, dtpB, caiF, narI, narJ, nrdD, yehC, fabR, hcp, yidF, frwC, hybB, ygeV, fliZ, ileS, recD, glcA, araB, yfaE, iraP, flgE,*

	flgI, yfjL, yecR, gltF, yceI, fetA, yobA, sapB, ydiH, pyrL, ynfC, mdtF, secG, rnlA, essD, glcB, yfdK, yoaC, yfiB, recB, bioA, rpoZ, yeaR, proW, yqjA, ghrA, frwB, proX, insQ, fucK, rihA, purT, rnb, gtrS, purN, gcvT, potB, aldA, gcd, ydiJ, dacA, waaL, nuoE, gatD, opgH, aceE, livG, ilvC, gcvP, oppC, livF, yedE, mprA, fruB, ptsG, hypD, ymfI, artQ, fecC, ndk, ybjE, mepS, csgD, hsdS, pqqL, ybdH, yqhA, fliE, fliR, cysN, yccJ, cysC, gltD, yahK, wrbA, yfbM, ftnA, manX, ycgB, mutS, gpr, oppF, dicA, mcrA, ppc, pntB, ribB, hisF, metG, csgE, araC, treB, yegE, putA, mtfA, proA, pepT, ruvC, rcsD, yieP, rbsD, livH, argG, yeeN, yebK, cydD, yeaD, sdaB, clsC, uvrC, yjfN, glyS, yjiX, fdx, yhjE, btuB, yedF, ebgR, epd, waaQ, blc, aroF, ampD, rhlB, fliQ, yddB, murF, gltB, cyoC, cyoD, pheT, ycdT, emrY, evgA, rihC, proV, ykgE, intD, feoC, gudX, dmsB, idnT, nrfB, hcr, yceM, yahB, gnd, nhaB, bamD, yncE, uvrY, truB, abgR, yliF, sieB, yeeL, mocA, fimA, priC, preT, intB, yeeJ, rutR, pppA, fepC, dinD, nlpC, pyrE, glnG, yadI, nrdA, ydfZ, azoR, djlA, yaaJ, zraS, fecE, sgcX, yabP, yoeA, emrE, fhuC, umuC, cysS, glmS, dld, glmU, ychH, manZ, ycdU, dcuB, hrpB, uspC, yhaH, moaD, araD, galS, ydhV, atpB, asr, yjiT, amyA, yfcE, elfA, yjgX, ycgH, srlA, malF, uspG, fimF, tdcR, glpB, yjhQ, malS, fliA, flgN, yhbT, lgoD, minE, ytfQ, yejG, ygcB, yfaH, smg, mrr, aphA, dmlA, yqeB, casD, holE, glnS, lptD, araF, fimD, ynjH, phoA, ttdA, yfdY, yihM, prmA, valS, ymdB, yecC, phr, mdtE, rnc, iclR, yfcU, ftsX, yciT, ebgC, yfjU, kch, cheB, pitA, gspD, gspF, fliO, ycbL, yebC, mraY, livK, ycbK, zapC, ydaS, malM, stfE, gspB, hiuH, degP, yehE, ydfO, malG, yceO, ppiB, pmrD, yhiD, yfeD, ydjZ, ygfK, slt, mscS, allS, yafK, yeeP, yeeX, yqiG, tnaA, tfaE, ghxQ, yfiR, ydfD, chbF, yffB, psiF, pabB, dtpC, ydiV, ycjV, gfcA, ybaQ, ryeA, yohJ, rmf, uidR, dapE, sseB, yfiN, fliD, yphF, rtn, eptB, nudE, ftsP, nrdG, yeaL, yfdE, ecpA, yrbL, yfjM, ykiA, yfeY, phoP, yjhD, yciB, yhcF, efeB, menE, murP, yfbP, ssuB, malQ, mug, rpoE, mlaE, pdxK, insO, yeaW, lptA, cydC, gspO, clpS, xylG, hns, entE, yjhH, fsr, yfjK, dctA, ygeW, yegW, smf, yaiL, fimG, ynbE, ytfP, ubiG, elaB, dxs, ygiS, ykfB, yidR, radA, yahM, dnaA, ldrD, mcrC, ygfF, insH25, nrfC, yhhJ, fucI, pgrR, yejA, yiaL, yihN, ftsZ, modB, yfcC, xylF, fecB, purB, prs, cyoB, purM, glyA, nuoC, purH, ybhC, pyrC, purF, fadL, cvpA, purC, gtrB, pyrD, xanP, purE, oppB, lysP, purK, tsx, accC, efeO, waaC, frmB, rsxC, yliE, fis, mglA, thiP, mipA, pheP, ampG, yeiP, metC, thiQ, lpxH, lipB, hsdM, pfo, thrB, thrA, atpF, trpE, lysC, metE, borD, artJ, alaA, icd, argD, yqgB, mgtA, speA, lpoA, dapB, murI, artP, gph, aroA, metK, mmuM, prc, potA, mqo, yidC, rluC, yibB, tdk, emrA, fiu, thiB, tig, metJ, tgt, tolQ, yqeF, potC, yibQ, ibaG, ybiT, wecB, cysD, cysP, gatC, tktA, yhbJ, plsX, pdxA, spoT, puuB, pnp, livJ, thiF, ymfA, yciW, atpA, gpsA, thiH, trpB, fbaA, cysK, folD, serA, pal, ydaM, groL, fdnH, ptsI, yagI, pstB, ydhK, znuA, oppD, ppsA, accB, glf, ridA, ydgA, rne, wbbI, pbpG, moaB, gcvH, ygjP, epmC, ghxP, gsk, menH, xseA, katE, ygaH, csgF, nac, stfP, yfjR, ddpD, appY, atpE, gntT, ratA, nanS, flk, talA, ybjS, mpaA, exuT, ypdF, uxaB, yggX, nth, hslU, yfcZ, modF, mppA, manY, kefF, ybjD, dmlR, mlc, eutR, yhaK, yneO, nfsA, menA, yfdT, gapA, moeB, yqhD, galE, tdcC, cbpA, ccmC, yhhX, pfkA, cspA, yfjW, plsY, gcvR, yjiY, ynfG, rsxE, glnQ, glyQ, yeeE, yjiN, rpsS, yccT, gatY, cheW, hpt, yhbE, ybiU, rluA, yeaK, menD, cheY, yheO, ybcJ, yihI, argA, nagC, csiE, agp, uup, yghB, glnL, ydiB, ubiI, wecA, ypjA, dhaL, nohD, tpx, mreB, zur, moaA, cspC, fdoG, yejM, fetB, torT, ybaP, hemB, iscX, ggt, ybdF, yigL, gspJ, ppx, fecI, rplM, rpsU, apt, yigI, mltF, adk, ygiM, yedV, alx, yccF, yhdT, puuA, ivbL, fabA, speC, mnmA, tsr, ycgZ, thiI, folE, asd, murE, yegU, rcsA, sodA, thiG, acnB, metH, trpA, cysM, hisC, hisG, glgB, hisH, rho, yghG, holA, symE, yniC, leuE, phoE, nagE, yfcD, tatC, cfa, opgD, ftnB, katG, fsaA, ydcH, pcm, ybiA, alaE, rayT, proB, hchA, ydbD, ygiZ, rcdA, yeaP, pheS, slmA, dinJ, fbp, pgi, fucP, ylbG, dapA, rhmR, bglX, moaE, yjaZ, gcvA, yafZ, dgoK, yacL, hdeB, hdeA, rhlE, slp, rsxG, racR, hdeD, fhuF, purA, lpd, gmk, ybiC, rsxD, rimM, yfcL, rpoB, rplQ, atpC, yqcC, yhbY, tyrB, gsiA, gadA, yhjR, pgaA, yejK, fimC, ybgS, cedA, nhaA, rutD, waaZ, fadE, yceK, rplJ, yfjH, ygfZ, treC, ycaI, ymfJ, glnP, ygcR, sdaC, yibA, yahF, yfbV, gadB, yiaB, astE, bhsA, sodB, hmp, yjbG, yqfA, feaR, fimH, uspE, yacG, ybeL, yjgH, minD, yegH, glnH, yadM, hflX, fabB, murD, yzgL, tfaD, cirA, caiC, ypjF, hisI, argI, yfjP, yebO, casE, entC, ydeM, ydgJ, sbmC, xdhA, ligA, hisJ, ydcD, ymgA, yaiV, gadX, hisL, yffM, yibN, cohE, yagB, secE, tktB, ldtB, glcG, mdtB, gpmM, ycaK, galU, ykgA, yidE, yjjJ, waaR, yphD, yggC, yibG, mqsR, rhsE, umuD, ribC, rimJ, ydjJ, ymfE, rnr, rapA, cmtB, dhaK, yiaT, ygiC, roxA, narP, mnmG, gyrA, ygbK, eutP, chaB, yrhA, ydeE, yeiH, mdtI, ydfI, aldB, lpp, glgS, dusC, narU, tufB, dcd, chpS, dcuS, ydfE, uxaC, yfhH, yiaJ, pliG, spy, tufA, thiM, chpB, moaC, atoC, yfgO, tyrR, gmr, yjhE, glk, trmI, aspA, yfdV, ydfC, lepA, wzzE, ydaW, yhbS, pstA, malI, dsdA, yceH, mltA, ykfA, tdcE, priA, yneJ, sapF, yjiV, adeQ, yobF, ompC, tpiA, miaA, qorA, ligB, macA, pmbA, ttcA, kdgK, znuC, lysU, dsdC, mngB, yceJ, yecS, creA, hemA, srlB, narL, yhhY, allC, entS, mog, yagF, yidL, pck, panC, treR, adeD, recE, ldtE, yqjI, ftsQ, aegA, acuI, yhaV, rsmD, recN, glcD, yhaO, rluF, ygbE, flgK, tyrA, uacT, ndh, yccS, hicB, ulaG, ogrK, ygeK, ybjT, yfeC, wzzB, yjgZ, yeaG, yihT, ais, lsrA, yhjB, fumC, yegP, msyB, fucA, yeeD, arnF, yaeH, fucU, mntR, psiE, hemN, ybiJ, fadR, citB, ecpC, yheT, lpxD, idnK, prkB, ybaL, hypA, ydiM, yeiI, yjcO, trmA, melB, glmM, speB, yfeO, ycfP, rimK, yqiA, rpmC, yggN, gapC, rstA, lolD, frsA, ycbF, phoB, rsmA, nlpI, yifK, zinT, hyaF, yeaY, yehY, ybfP, skp, recO, ydcL, rssA, flgM, fruK, yqiC, ydgH, secD, recX, ygiB, galR, mzrA, insA8, hisM, minC, yidB, rffH, slyD, crp, yqgE, aspC, glsA, yjiG, yghU, hisQ, pdhR, cheZ, eutJ, ubiJ, ycjP, yciI, fhuA, nanR, hisB, rffG, gltT, yjdC, rimP, rspR, hypT, dnaT, fldA, ygjH, alkB, folP, psd, fdhE, era, wecC, gatR, pepE, ykgM, fliY, yrdB, tatD, tadA, fabH, yejF, gadW, yraJ, aroP, yrdD, allD, yagN, cra, artM, yfcA, yebW, yeeA, pspF, yjgA, elfC, fruA, rpmI, uspF, yehU, yiiE, glpD, yciN, yqgC, birA, yeaM, yibL, yecT, gntP, ycjT, mrcB, mltD, yihG, ybgE, ytfI, mcrB, ygeF, yhcO, holD, serB, sslE, perR, dedD, ettA, zapB, yihY, yjhP, hcaC, yeaJ, yehI, yahI, hemD, ydhU, glgP, fur, ycaL, yhgN, cybB,

	tusB, lrp, eutH, ccmE, cutC, ybhJ, hscB, yggS, ymfM, trkA, ybfE, rseA, crfC, yehL, ydeT, grxC, garR, rraB, mukB, nikR, tatA, ydiE, xylR, glpC, chbA, yahG, yggW, phnL, sgbU, yciK, rsmH, hyfC, cspG, cysE, sapD, mqsA, pbpC, mazG, djlC, ydjF, csrD, pepP, yhjJ, rplY, yfeW, csgG, slyX, lon, caiB, aas, secB, yqiJ, rng, yfcF, tdh, yfbU, argP, arnD, yoaB, elbB, ymfL, puuR, yahE, yfiF, ygaQ, insN, ybjL, ccmD, ygjJ, maa, chaA, ytjC, rcnB, yhhS, brnQ, dgkA, ynaJ, sgbE, ppiC, mokB, ygfT, yhhM, yafE, envY, glcC, yeaE, fadJ, fadA, ydcS, yffL, yihA, tesA, fecD, yigA, fsaB, ybcL, yhjV, malZ, malX, araE, yjaA, yfbN, arsR, yidI, modA, glnB, mutH, eutN, ydjH, yfcP, ydjG, cpdB, ssb, ychF, ykgG, eda, yfaD, tabA, bolA, mscK, cbl, uvrB, yhjY, iadA, ecpB, yjiJ, idi, yieE, ascG, gcl, rrrQ, epmA, fes, feaB, nanA, yicI, frlR, yjeJ, bamE, sfmC, xisE, yhdU, mnmC, csrC, ybbD, ycgX, gnsB, ycaM, yeiS, lpxT, yfeS, yqjC, yedZ, holC, cspI, ytfK, dcm, mdtG, fimB, folB, apaG, dkgB, cysB, cyaR, glpG, glsB, ymgC, ynfA, mioC, ddlB, glyU, rem, yjfZ, yijD, yciQ, ynjB, rpsO, sibB, yiaG, srlD, mdtJ, slyB, glcF, beeE, yrhB, thiD, htrE, yghW, dsbA, rrsG, arfB, trxA, iscU, gstA, sibC, yeaN, iscA, jayE, fabZ, ycjD, gadC, php, ygbT, yhfK, csrB, gmhA, yfjS, sgcA, ydgC, gspK, cueR, bglA, ydcZ, yncJ, iscS, eamA, bamB, rcsF, ybiX, ygbF, ryjA, blr, chrR, csgB, lsrK, pnuC, pinR, guaC, idnO, yfiE, yhcD, yggT, prlF, mdtK, lpxA, zraP, trmL, rlpA, mreD, insJ, rhsA, hokE, yjdJ, yjiH, ymcE, yoaF, rutF, grxD, mazE, yegZ, hha, gfcC, lsrG, yggU, yqfB, ydfG, mnmE, ydhZ, murC, rimI, hokB, der, yhgA, insZ, isrC, yfgF, yfdN, acs, yjdI, yqeK, plsC, yegX, alr, psrD, yhcE, ydaN, spf, ydcC, ygiL, ybcY, uhpT, pheM, aidB, nfo, yhfZ, ppk, proS, ebgA, ydfV, nanC, ratB, clpB, ybeY, ygdG, rpoN, mnmH, allR, rlmF, rrrD, rna, waaP, ycgJ, rplU, yjbD, insF1, yhiN, ypjD, aaeX, agaB, uvrD, rpmJ, rpmB, gshA, yjtD, recJ, ydhI, ispB, yihS, ytfT, yhcG, cobT, yfdQ, ynfB, yhiI, xylH, recA, wzc, bcr, yegI, cspH, mukF, ydbL, ytfJ, relA, pitB, ydcA, pyrH, ypdB, rraA, gss, ybfG, tmcA, mdtL, yicG, yphA, gor, citA, ydhJ, sohB, sbcB, mlaF, yjgN, yhjK, insI1, ugpA, actP, yeaX, yjcH, entB, ydeQ, mntH, yraR, yfcQ, csgA, glnD, dnaC, phnE, rpmE, wbbH, yobB, rhaR, mscL, menC, insI4, nrfE, idnR, yeeW, nirB, yegT, yfeK, flxA, yhiM, ygcN, yfjX, cpxR, nsrR, cvrA, ybiI, inaA, fhuB, ybcW, ydbA, ldtD, acpP, ribE, kdgT, gpmA, sgcE, yjhI, ygbL, clpX, yjiK, umpH, yadL, ydaG, kdpC, nadB, yhaI, ydaY, yehX, yahC, murB, mraZ, panF, yjhV, ybhL, ygcE, glvC, yjcC, ybfF, ydjI, yagH, ykgF, def, yoaG, rlmD, ycaN, ycgI, yffP, ribF, ftsI, yhhI, ydhQ, yfgI, alaS, ybaM, insB1, gspE, ynbD, sulA, yciU, polA, ydcR, mrdB, ftsW, coaE, yggL, cyaA, btuC, pgaB, gltS, croE, mpl, yjfL
**Nitrosative**	*metF, metA, purE, mglA, metR, metB, rnpA, mmuP, purM, metN, sdhC, metK, xanP, flhD, ybdL, pyrD, fadB, cyoA, purK, fliE, mepS, metL, plaP, fliR, hisR, fliQ, fadI, dusB, ampG, thiP, ydiY, purH, rpsT, yibQ, valZ, pyrC, yciH, yliE, lldP, yigI, codB, argQ, ydcI, purF, hflD, ybjE, fis, rpsL, fadL, rlmA, ndk, yiiX, yceA, prfC, ygiQ, pstS, purT, ibaG, mmuM, mglB, uraA, yncD, recQ, pyrF, suhB, rimO, rsxA, ybdH, leuP, gtrA, queD, efeO, sdhD, yoeG, aceB, yecJ, yeiP, cvpA, upp, apt, queA, metI, yeiB, purD, lysZ, flhC, lpxH, mrdA, lysA, kgtP, potA, alx, tsgA, yqeF, nepI, rpsG, speC, mipA, csgD, purR, eptC, rsxC, rplM, fklB, rbsA, puuA, fiu, mreC, purB, puuP, tsaA, rph, gcvT, argV, ybhC, purL, evgS, thiQ, bioD, adk, metC, hsdS, glyA, efeU, mltF, carA, yqhA, stpA, fliJ, rpsU, sbp, codA, waaH, yneE, potB, rluC, gtrB, yidC, rsxB, ycaO, purN, yhdT, potC, ybiT, ymdA, gcd, rplC, rnb, trmN, metJ, tsx, yecE, yahA, tig, yibB, rpsJ, yhgF, metQ, valT, rpsA, prs, thiB, lysP, typA, pcnB, mhpR, ygiM, lipB, frmB, aceK, can, pheP, yedV, waaC, tsaB, cysJ, potD, rpsI, mqo, mnmA, rarD, glyT, gtrS, recR, aroA, emrA, speD, yadS, rpmH, murA, arnC, nuoC, yoeH, tolQ, bluF, cmk, hsdM, glpF, ydcP, fliP, trxB, pstC, oppB, waaL, tyrP, uhpB, tdk, argX, gatA, wecB, fliI, aldA, serC, mglC, uhpC, ycgZ, rlmN, cyoB, rplD, prc, accC, thiI, dacB, yegQ, pth, tgt, ydiJ, ubiX, nuoA, yccF, argZ, tolR, miaB, ynjE, tsr, recG, yciA, fumA, ivbL, efp, purC, ispA, rlmH, fabA, pqqL, gluQ, yddB, eptA, dacA, gpt*
**Oxidative**	*narG, garL, rcsA, yegU, yagU, pfo, napH, gtrS, ycbJ, gldA, purB, yahN, prs, yagE, hycG, ycgB, yccJ, tap, mokC, ydaM, narH, emrY, wrbA, gsiB, glyA, yqeI, dinJ, ygiZ, ybeQ, holA, rpsH, damX, yjjB, manX, yqeC, lgoR, frmB, puuP, ydaT, leuE, gadE, rplF, ymfA, ydiL, ftnB, ydcH, puuB, paoA, gudX, ycaC, fliT, eutK, ysaA, mcrA, ydeN, yfbM, ravA, fliS, cdd, nemR, napA, fadL, ygcW, opgD, evgA, ykgE, symE, ycjR, fdnH, fliP, pcm, garD, fsaA, betI, folK, napD, yfbR, hcr, rihC, alaE, ybiA, yifB, rayT, rpsN, hsrA, yeaP, ansB, ylbG, polB, menH, mhpF, dosC, metF, yodD, hybO, nrfB, idnT, ydbD, mprA, pyrG, dmsB, folD, cheR, lsrD, ybhH, prfC, ycdT, yahB, ftsY, citC, mmuM, ybiW, ybgF, yjaZ, gatD, rcdA, melA, srlQ, eaeH, adiY, purT, gcvA, nuoC, garK, phoE, yiiS, napG, ribA, cusS, emtA, proV, yadK, tatC, fruB, rhsH, hypD, rhmR, yafZ, yeiE, fucP, frdD, ptsI, dgoK, intD, yacL, rnpA, yijO, prc, cyoB, ydhK, pheS, paaF, fucO, ydeA, opgH, pstB, tdcA, talA, gpsA, bcsQ, fryC, fbaB, yahK, metR, rhtC, torC, gudP, flgJ, rsxE, sppA, garP, kdpA, ydiT, waaL, ruvC, yegL, caiA, clsB, oppB, cspA, ydhY, upp, csgF, pspB, alkA, yeaH, nanM, nrfA, ybcJ, intF, ybhK, purN, ghoS, yeeL, yceK, flgL, mepS, pgaA, rutE, otsA, pspA, bssR, dgt, dusB, yhfX, yjjI, rplD, deaD, narK, yeeT, otsB, plsY, napF, gadA, yiiG, metN, yagB, fumB, hycH, flgG, yniA, wza, allA, gtrB, abrB, dld, casE, yciO, pyrI, yraQ, dcuC, yhjR, nadE, atpF, gatB, tdcB, cusC, gltK, yjfF, yfjR, astD, oppC, ygcP, psuT, purH, exbB, yidD, yjeT, frr, nac, rpsK, nikB, acrR, dksA, purC, ydhW, ygeX, yjfJ, ylaC, yfjW, ompW, ybjE, yjdM, gatA, hypB, ycjG, lacA, scpC, suhB, ybaE, gcd, ykfA, leuO, yhhW, purM, yecN, yehD, yncI, gatZ, kup, mocA, asmA, rplW, ynfM, nohQ, fis, rpsP, ycfJ, pnp, yjiM, priC, yjgH, narU, hslO, guaB, dtpB, gadX, cspB, rnb, amtB, fepC, yahF, agaR, gdhA, ibpB, srlA, cedA, fepD,*

	aceE, opgC, frwA, rutD, yhaJ, pyrC, ynfE, rplJ, trmH, epmC, yjjP, yiaV, melR, fadD, cynX, atpH, ubiX, nirD, yibB, mdtH, stfP, rpsD, cdaR, ybgS, ydcN, yffM, pspE, rplB, ycaI, ydjL, rplX, pheP, yqgA, ygcR, fimH, yciX, kefF, ttdR, lit, rpsT, phnG, yehT, carA, yiaD, potE, ccp, rsmC, quuQ, yqgB, ynfK, yiaW, lptE, mhpT, kdpB, atoS, ygaY, ybhC, yecH, sfmA, yfcJ, bglB, fbaA, yidJ, ygjR, yjiP, hflC, lexA, yfgO, cueO, pepT, ybiR, nhaA, miaB, csgE, ppdB, cheA, narI, lplT, yhbU, yhcB, pyrB, narJ, xylE, yfbS, yibN, yafX, znuA, glf, intB, ybeM, fdnG, ymfI, ydbC, waaZ, ftnA, ycaO, yafS, yfjV, ydaV, xapA, artJ, accB, yjfN, lpp, oxyR, ydcJ, evgS, uspE, ribC, nuoF, gsiC, preT, eutP, rlmC, plaP, bioD, yehC, gpr, livG, eutR, kdsB, yphC, yacC, atpE, yqfA, yfdV, cspF, rpsS, sgcX, tdcG, ybdJ, pykF, frdA, kilR, yfjH, yaiS, clcB, acpS, allB, yfbV, yghR, artQ, ybeL, glnG, ylbF, ispH, patD, hypC, caiF, amyA, yjjJ, hinT, nth, pka, cheY, nrdD, fliM, yccM, agaD, sbcD, rplE, yaiV, wzzE, alsE, oppA, uspC, tgt, yadM, ydfC, thrU, glyQ, yidE, betT, yneG, oppD, yaaJ, tnaC, shiA, cspC, xdhA, lacY, plsX, cspE, hcp, yfjP, yagA, yahD, cirA, rsmE, rpoA, dauA, yhcC, cysZ, amiC, yheO, ybjM, tdcR, dcuS, rplN, ydjY, manZ, rph, hcaF, topB, proA, rfbC, rutR, zraS, wzxB, mngA, yadI, yedI, purD, yebF, fhuC, ydaW, yneE, mhpR, murI, yidF, yiaB, ymfE, yecS, yhbQ, wcaL, atoD, lpoA, dppB, fryA, ybjO, ymfJ, srlR, glcG, lysC, afuB, ychH, gadB, leuU, yaiY, cysS, rpsG, hldE, yedA, trxB, cvpA, yehP, yfeZ, ddpD, gcvP, ycdU, sbmC, gyrA, ydiQ, ymfR, nanS, potB, yjiS, manA, fliK, ratA, uxaB, ydfZ, yafL, yafY, fadK, yffN, oppF, yedE, metE, sucA, ygdH, casA, thiP, envZ, rnlB, yhdX, murR, waaC, glgS, yneL, ybdN, rsmI, guaA, pgaC, miaA, yabP, dhaK, yghA, mhpC, araC, rcsD, cdh, nirC, yhbV, waaR, yncG, livM, yibA, bhsA, cysJ, thiF, ilvY, aldB, yfaH, marA, gpmM, parC, idnD, ppiA, galP, serC, ligA, yhbS, yaaX, blc, astE, ftsQ, paaG, nuoE, dcuB, ydcO, ypfN, uspD, waaQ, yjhB, ttdT, nuoI, hybA, yagF, ypjK, ydeJ, ynfD, marB, dctR, argQ, insP, pqiA, gntT, speA, ybiH, hflX, ppdC, yegE, cheW, prpR, recF, tusE, yedL, lhgO, yjiN, lgoD, flgK, ybbY, yjiA, flk, ribB, tig, argD, chaB, mdtI, ftsN, yahL, intZ, ndh, ygiC, priA, kptA, malY, ygcS, rne, dinD, casB, cobC, yaaW, yebO, fdx, panC, yeiW, elyC, appY, yeiB, metI, ybgA, treR, yadC, rimO, mlc, ydgA, allC, mlaB, yfjQ, oweS, malK, hsdS, yebY, pphA, hydN, flgH, yphD, zapE, pheL, uxaC, rlmE, pyrE, argV, ykgL, ybcO, ppc, glyS, phnP, ydhV, ykgH, ykfG, feoA, yccS, gspH, ulaE, trpD, hslU, yeeN, malI, malP, mdfA, uvrA, purL, cho, nuoB, csiE, sucB, gcvH, frdC, spy, eutS, ibpA, ygfI, rpsC, insG, ydhB, tfaD, dmlR, ubiI, sgcC, fliC, ygeK, hflD, ldtC, rarA, rhaM, thiL, norV, yzgL, secE, ycjF, yaiW, yegD, fabR, bdcA, endA, atoE, flhA, glk, fepE, ygeV, caiC, alsR, yfjD, ybaV, yeeR, nagC, hisL, yhbT, ybjH, rzpQ, groL, frdB, hrpB, atpB, tolQ, mutT, lafU, tauD, yhhY, ybfA, apbE, nuoJ, cbpA, nikA, ydaU, yebZ, eptC, sufA, ygbJ, ninE, rlmN, napB, xseA, ypjF, entC, atoC, ykfC, yfbT, wbbI, uspG, yegP, ynaK, bamC, edd, ydcP, argS, ygbM, chpB, ybiV, ypdF, zur, qorA, pmbA, argZ, ydeM, lsrA, rsxB, ytfQ, mrr, zipA, amiB, yfiL, yebQ, yhbE, wecD, livF, mtfA, yiaT, fabI, ydiJ, ggt, malS, rlmB, pepA, aaeR, purF, putA, dmsC, ydcD, ybjD, yghB, wecA, rluF, chpS, ligB, murA, yebS, yejG, argY, kbaZ, mglA, hypE, tyrP, yceQ, tolA, fkpA, yfcG, yeaG, iap, lysR, yieP, ynfF, paaE, rimL, yaiP, narQ, mppA, fimD, lepA, yraN, yhjX, tyrT, psiE, flgN, yagL, cydA, yhjE, rclB, mutY, ygeO, pntA, hybB, modC, clcA, trpE, ybhM, atpA, yfeC, hyaD, mdtM, treB, borD, fusA, yaiX, fliA, smg, relE, feoB, yjjW, dusA, hemY, nrdI, mpaA, rlmG, yifN, chbG, napC, rutG, rplP, yoaA, pagP, yjgR, gcvT, yqgF, ampD, ybaY, holE, ydfU, mutS, tsaB, sufC, yidL, dsrB, ygcB, yggC, gtrA, glpX, rnr, ldtE, phnH, truD, gstB, glnS, sapF, uspA, yigE, torT, araD, citX, yciM, metB, ybjC, manY, hemA, nusB, adhE, dapB, dacA, ygaU, ugd, alaA, yhbJ, arnF, prpD, oxc, dppD, acuI, hrpA, tyrV, emrE, yedY, spoT, acrZ, yeeY, yoeA, yqjI, dgoR, fabG, yeiP, yheT, yedS, ycgR, nuoH, mscM, yfaQ, ybbC, fadM, yfiP, yobD, yibG, araB, yieH, ybeZ, rplV, yfcZ, rsmJ, uup, yjgX, mutL, glnA, mfd, yrhA, abgT, yphG, pfkA, plsB, yagM, yjgL, ydiM, nagB, yciE, gltL, dsbE, yidA, ileS, cmtB, narW, ispF, ligT, mqsR, rhsE, hscA, ispD, fpr, wzzB, yjcO, yghE, yfeX, yjjQ, citD, trpL, yqeB, ybaK, dsbD, ydcV, ccmC, gph, ytfA, lpxB, rnhA, potG, yhhX, yjiV, ybiJ, prpB, yidP, recD, yhaO, mntR, fixB, ycjS, carB, gltD, nudJ, mnmG, yigL, yjhE, yhhA, glcA, moeB, ilvC, mdlA, fliF, yoaE, vsr, ybhD, thrB, ybdL, aegA, rbsB, qseC, yagI, ampE, cydB, thiS, pldB, mgtA, yafW, mdtP, ytfE, rpmG, yafV, aphA, rpsQ, rcsC, yajO, yjiR, codB, ydeE, dcd, idnK, ygfM, ychQ, pal, yhaV, ttdA, ybjS, murQ, yafQ, gltJ, ldcC, rlmM, thrA, fixC, secY, yggP, ansA, pdxA, mtlR, araF, sapC, ynjH, yfeO, yrbG, yniB, lpxC, hisF, ecpC, srlM, accC, dinF, rof, phnK, fucA, hicB, xdhB, ybgD, waaS, trpB, frwC, fliZ, adeQ, uxuR, nadR, ldhA, atpG, lysU, ygbI, ybcI, ompF, uspB, ulaG, pptA, yieL, elfA, melB, pepN, tyrS, tdcC, xseB, ycfP, yfaA, ssuC, poxB, tolB, menA, astB, tsgA, moeA, ppx, hisS, ftsA, yajQ, trpC, yfdY, dicB, ycdY, ampG, mreB, yihO, fiu, yceJ, modE, yqhD, yggX, ycgH, yfjJ, wcaJ, gapA, livH, rclC, yaiE, ddpC, holB, yccT, rfbA, yaeH, luxS, cysH, yliE, hybC, rplR, aes, rcnR, prkB, epd, phoA, ycbF, yicH, yfjY, ybdF, fabD, grpE, exuT, icd, ygdB, yagK, purR, yaaA, iscX, ymgE, potF, thiE, nadC, bfr, ugpQ, rhtA, fecC, ccmH, artP, pcnB, hsdM, exuR, topA, msrA, yneO, thrT, wcaG, rhmA, fumC, yjcE, yhaK, nfsA, yggN, recQ, rpiA, nusA, frsA, setA, ptsG, ybaL, prpC, pflB, solA, rsxC, sra, yihM, abgA, yagJ, hflK, yfhM, flhB, rplA, creA, ybhQ, rhlB, yehH, ilvE, hemX, sokC, galE, narZ, lpxD, citG, ypdI, murG, dnaK, udp, betA, chiQ, frmA, yeiQ, casD, sdiA, rmuC, serT, mhpD, rtcR, moaA, lptD, yobF, ybiO, yddG, yfdT, hycA, malF, codA, modF, phoB, kefB
